# A novel long-tailed myovirus represents a new T4-like cyanophage cluster

**DOI:** 10.3389/fmicb.2023.1293846

**Published:** 2023-11-09

**Authors:** Yuanfang Liu, Xue Meng, Hongrui Zheng, Lanlan Cai, Shuzhen Wei, Minglu He, Jiale He, Yue Hao, Chang Ge, Jihua Liu, Feng Chen, Yongle Xu

**Affiliations:** ^1^Institute of Marine Science and Technology, Shandong University, Qingdao, China; ^2^Department of Ocean Science, The Hong Kong University of Science and Technology, Kowloon, Hong Kong SAR, China; ^3^State Key Laboratory of Marine Environmental Science, Fujian Key Laboratory of Marine Carbon Sequestration, College of Ocean and Earth Sciences, Xiamen University, Xiamen, China; ^4^School of Information Science and Engineering, Shandong University, Qingdao, China; ^5^School of Life Science, Shandong University, Qingdao, China; ^6^Institute of Marine and Environmental Technology, University of Maryland Center for Environmental Science, Baltimore, MD, United States

**Keywords:** T4-like cyanophages, cluster C, longest tail, auxiliary metabolic gene, virion-associated protein, ecological distribution

## Abstract

Cyanophages affect the abundance, diversity, metabolism, and evolution of picocyanobacteria in marine ecosystems. Here we report an estuarine *Synechococcus* phage, S-CREM2, which represents a novel viral genus and leads to the establishment of a new T4-like cyanophage clade named cluster C. S-CREM2 possesses the longest tail (~418 nm) among isolated cyanomyoviruses and encodes six tail-related proteins that are exclusively homologous to those predicted in the cluster C cyanophages. Furthermore, S-CREM2 may carry three regulatory proteins in the virion, which may play a crucial role in optimizing the host intracellular environment for viral replication at the initial stage of infection. The cluster C cyanophages lack auxiliary metabolic genes (AMGs) that are commonly found in cyanophages of the T4-like clusters A and B and encode unique AMGs like an S-type phycobilin lyase gene. A variation in the composition of tRNA and *cis-*regulatory RNA genes was observed between the marine and freshwater phage strains in cluster C, reflecting their different modes of coping with hosts and habitats. The cluster C cyanophages are widespread in estuarine and coastal regions and exhibit equivalent or even higher relative abundance compared to those of clusters A and B cyanophages in certain estuarine regions. The isolation of cyanophage S-CREM2 provides new insights into the phage–host interactions mediated by both newly discovered AMGs and virion-associated proteins and emphasizes the ecological significance of cluster C cyanophages in estuarine environments.

## Introduction

Cyanophages, which infect cyanobacteria, are an important component of marine viruses and play a crucial role in marine ecosystems by influencing the population dynamics, community structure, metabolism, and evolution of cyanobacteria (Mann and Clokie, 2012). Cyanophages isolated from marine ecosystems can be classified into three morphological groups: cyanomyoviruses, cyanopodoviruses, and cyanosiphoviruses (Sullivan et al., 2005, 2009, 2010). Among the three groups of cyanophages, cyanomyoviruses are the most frequently isolated, with the T4-like as the dominant member (Wang et al., 2023). It is estimated that T4-like cyanomyoviruses are the most abundant and widely distributed cyanophages in marine environments (Mann and Clokie, 2012; Huang et al., 2015). About 106 T4-like cyanophage genomes have been sequenced (Jiang et al., 2020; Wang et al., 2023) and are found possessing a collection of core genes associated with DNA replication and structure forming (Sullivan et al., 2010). Marine T4-like cyanophage isolates were previously classified into two lineages, namely clusters A and B, based on phylogenetic analysis of the core genes (Ignacio-Espinoza and Sullivan, 2012). The freshwater cyanophage S-CRM01 exhibits high homology with the marine T4-like cyanophage isolates but is divergent from the previously identified clusters A and B in terms of phylogenetic lineage, which sheds new light on the phylogeny of T4-like cyanophages (Dreher et al., 2011). As more cyanophage isolates are discovered, the classification of T4-like cyanophages necessitates further refinement.

The T4-like cyanophages usually encode auxiliary metabolic genes (AMGs), which play important roles in regulating host metabolisms to increase the phage fitness (Sullivan et al., 2010). The AMG composition varies greatly among T4-like cyanophages. A large number of T4-like cyanophages, such as P-SSM2, S-SCSM1, and S-SZBM1 (Sullivan et al., 2005; Rong et al., 2022; Wang et al., 2022), encode a variety of AMGs, including those involved in photosynthesis (*psbA*, *psbD*, *hli*, *petE*, *petF*, *ptoX*, *ho1*, *pcy*, *pebS*, and *cpeT*), carbon metabolism (*talC*, *gnd*, *zwf*, *cp12*, and MPI), phosphorus acquisition (*pstS* and *phoA*), vitamin B12 synthesis (*cobS*, *cobO*, and *cobA*), and cell wall synthesis and modification (GDP-mannose glycosyl hydrolase, GDP-L-fucose synthase, and GDP-mannose-3,5-epimerase) (Sullivan et al., 2010; Wang et al., 2022). Furthermore, multiple copies of certain genes have been observed in the T4-like cyanophage genomes. For example, three *hli*s were found in the P-SSM2 genome (Sullivan et al., 2010), 24 2OG-Fe(II) oxygenase family genes are predicted in the S-SCSM1 genome (Wang et al., 2022). Additionally, some AMGs are prevalent in the T4-like cyanophage genomes. Over 90% of T4-like cyanophages encode *psbA*, *hli*, *phoH*, *mazG*, *cobS*, and *hsp20*, while more than 60% of T4-like cyanophages encode *psbD*, *talC*, *petE*, *cpeT*, and *cp12* (Jiang et al., 2020). However, some T4-like cyanophages encode a limited number of AMGs. S-CRM01 only contains seven AMGs that are commonly found in T4-like cyanophages. S-H34, S-N03, and S-B68 even lack the most commonly observed photosynthesis-related genes, *psbA* and *hli,* in T4-like cyanophages (Jiang et al., 2020). Different AMG profiles in cyanophage genomes can have diverse influences on host metabolisms. An illustrative instance involves cyanophages S-RSM4 and S-PM2. S-RSM4 has multiple genes related to carbon metabolism (*talC*, *gnd*, *zwf*, and *cp12*) and stops the host’s carbon fixation reaction 2 h earlier than S-PM2, which lacks these carbon metabolism-related genes, resulting in much less carbon fixation in the S-RSM4-infected cells (Puxty et al., 2016).

tRNA genes are widely distributed in the T4-like cyanophage genomes, with numbers ranging from 0 to 33 (Enav et al., 2012). Enav et al. propose that phages carry tRNAs to optimize the codon usage discrepancy between phages and hosts, enabling phage cross-infectivity of hosts with divergent G + C contents (Enav et al., 2012). In addition, cyanophage tRNA genes are predicted to facilitate the expression of specific AMGs (Enav et al., 2012; Xu et al., 2018). Cyanophage genomes also contain non-coding RNA (ncRNA) genes with regulatory functions. Currently, T4-like cyanophages have been found to contain several *cis*-regulatory RNAs, including *glnA, manA*, PhotoRC-II, and *wcaG*, which are thought to play a regulatory role in important host bioprocesses, including photosynthesis, nitrogen metabolism, and exopolysaccharide production (Weinberg et al., 2010; Wang et al., 2022; Zheng et al., 2023).

Generally, virions (i.e., an infectious virus particle) consist of structural proteins. However, certain viral proteomes have been found to encapsulate non-structural proteins related to host metabolism regulations. The most well-known phage non-structural protein is the RNA polymerase in N4 podoviruses (Falco et al., 1980; Kazmierczak et al., 2002). Virion-associated protein kinases (VAPKs) have been identified in various virus families except for dsRNA and ssDNA viruses. Especially in animal and plant viruses, VAPKs are prevalent and play crucial roles in multiple stages of the viral life cycle, including infection, uncoating, transcription, and replication (Hui, 2002). Furthermore, VAPKs have also been detected within the virion of cyanophages. For instance, putative serine/therine protein kinases have been identified in the S-CRM01 and S-TIM5 virions (Dreher et al., 2011; Sabehi et al., 2012). APH, ChoK, and Rio2 kinases were detected in the virions of a non-T4 cyanomyovirus, S-CBWM1, and are predicted to be involved in host bioprocesses like antibiotic resistance, protein binding to phospholipids and choline, and ribosome biogenesis (Xu et al., 2018). In addition, nicotinamide/nicotinate monomucleotide adenylytransferase (NMNAT)-like proteins are found in the virion proteomes of cyanomyoviruses, S-CBWM1, S-SZBM1, and S-SCSM1, which are thought to be involved in NAD^+^ synthesis during infection (Xu et al., 2018; Rong et al., 2022; Wang et al., 2022). These regulatory proteins are thought to create an optimal environment for viral replication upon entry into the host cell.

The discovery of novel cyanophage isolates is always enhancing our comprehension of viral genetic diversity, evolution, phage–host interactions, and potential ecological functions. Here, we characterized a new T4-like cyanophage, S-CREM2, which was isolated from the Changjiang River Estuary. S-CREM2 represents a new viral genus and possesses the longest tail ever found in cyanomyoviruses. The identification of S-CREM2 promoted the establishment of a novel T4-like cyanophage lineage, referred to as cluster C, which is as prevalent as the previously identified clusters A and B in the estuarine environment. The isolation and characterization of S-CREM2 provide new insights into phage–host interactions and the ecological distribution of the newly established cluster C cyanophages.

## Materials and methods

### Cyanophage isolation

*Synechococcus* sp. CRE1902 was isolated from the surface water of Changjiang River Estuary (31.52°N, 122.64°E) in July 2019, and used as a host organism for cyanophage isolation. *Synechococcus* sp. CRE1902 was grown in seawater-based SN medium with a salinity of 25‰ (SN25) (Waterbury and Willey, 1988), and incubated at a temperature of 22°C under a constant cool-white light intensity of 20 μE m^−2^ s^−1^. Cyanophage S-CREM2 was obtained from the surface seawater sample collected in the Changjiang River Estuary (31.31°N, 122.49°E) in July 2019. The viral seawater used for cyanophage isolation was prepared by a 0.22-μm filtration to remove bacterial cells and subsequently stored in the dark at 4°C until further use. Cyanophages were first enriched by adding 20 μl of the above viral seawater to 180 μl of exponentially growing *Synechococcus* sp. CRE1902 cultures (optical density at 750 nm (OD_750_) = 0.5) in a 96-well microtiter plate. After the lysis of *Synechococcus* cells, the lysates were collected and centrifuged at 10,000 × *g*, 4°C for 10 min. The supernatants were filtered through 0.22-μm-pore-size sterile syringe filters (Millipore, Millex®-G, USA) and subsequently used for phage purification. Phage purification was performed using the plaque assay method (Suttle and Chen, 1992) and repeated three times.

### Host range determination

Nine *Synechococcus* strains were used for the host range detection of S-CREM2, which included five estuarine strains, CB0101, CRE1901, CRE1902, CBW1107, CBW1101, and four oceanic strains, CC9311, WH8102, WH7803, WH7805. About 20 μl of S-CREM2 suspensions were added to 180 μl exponentially growing *Synechococcus* cultures in 96-well microtiter plates in triplicate, while the controls received 20 μl of SN25 medium. All plates were incubated under the same condition as described above and observed daily for cell lysis.

### Phage amplification and purification

To amplify S-CREM2 phage, phage suspensions were added into 2 L of exponentially growing *Synechococcus* sp. CRE1902 cultures (OD_750_ = 0.5) at a multiplicity of infection of 0.01. The resulting lysates were treated with DNase І and RNase A both at a concentration of 2 μg mL^−1^ at room temperature for 1 h. Subsequently, the NaCl concentration of the lysates was adjusted to 1 M, and the lysates were ice-bathed for 30 min (Xu et al., 2015). The treated lysates were then centrifuged at 10,000 × *g*, 4°C for 20 min. The resulting supernatants were filtered through 0.45-μm-pore-size polycarbonate membrane filters to remove cell debris. Phage particles in the supernatants were concentrated using 10% (w/v) polyethylene glycol 8,000 at 4°C for 24 h and then precipitated by centrifugation at 12,000 × *g* for 1 h. The resulting S-CREM2 pellet was resuspended in TM buffer (20 mM Tris-Cl and 10 mM MgSO_4_) and subjected to CsCl-gradient centrifugation (200,000 × *g* at 4°C for 6 h) using a SW 41Ti rotor (Beckman Optima L-100XP, Beckman Coulter, CA, USA). The visible phage band was extracted and underwent a 30-kDa centrifugal ultrafiltration to remove CsCl from the phage suspension.

### Transmission electron microscopy observation

Ten microliters of the CsCl-purified phage suspension were absorbed onto a 200-mesh carbon-coated copper film for 1 min. Subsequently, they were negatively stained with 2% (w/v) uranyl acetate for 30 s. The excess dye was gently removed using filter paper, and the staining process was repeated. After drying for 30 min, the prepared sample was observed using a Tecnai G2 Spirit BioTwin transmission electron microscope (FEI Tecnai G2 F20, Thermo Fisher Scientific, Waltham, MA, USA). The Xplore3D image transmission system (USA) was utilized to capture high-quality images of the phage particles.

### Phage DNA extraction and genome sequencing

Phage particles were first treated with a cocktail buffer containing proteinase K (100 mg mL^−1^), SDS (10%, wt/vol), and EDTA (0.5 M). Subsequently, phage DNA was extracted using the phenol-chloroform method as previously described (Chen et al., 2006). A whole-genome shotgun strategy was used to construct the PE150 library. The obtained raw data were subjected to quality filtering, trimming, and *de novo* assembly using IDBA v1.1.3 (Peng et al., 2012) and megahit v1.2.9 (Li et al., 2016). Any remaining gaps in the cyanophage genome were closed using pilon v1.24 and bcftools v1.17 (Narasimhan et al., 2016). The complete genome sequence has been submitted to the GenBank database under accession no. OR473000.

### Genome annotation and comparative genomic analyses

The putative open reading frames (ORFs) of S-CREM2 were predicted using the GeneMarkS^1^ (Besemer and Borodovsky, 2005), the RAST server^2^ (Brettin et al., 2015), and the MetaGene Annotator^3^ (Noguchi et al., 2008). Translated ORFs were annotated by combining the results of homolog search against the NCBI non-redundant (NR) database, conserved domain prediction, and remote homolog search using the HHpred server^4^ (Söding et al., 2005). ORF homolog search against the NR database was conducted using BLASTP with an e-value cutoff of <10^−5^ and a bit core of >40 (Pruitt et al., 2007). Conserved domains within ORFs were predicted by searching against the NCBI Conserved Domain Database (CDD) (Marchler-Bauer et al., 2011), with an e-value cutoff <10^−3^, a bit score of >40, and a coverage of >40%. For ORFs without predicted conserved domains, HHpred search against PDB_mmCIF70_18_Jun, UniProt-SwissProt-viral70_3_NOV_2021, SCOPe70_2.08 structural/domain databases was conducted, with a probability cutoff of >90%, to supplement the ORF annotation. tRNA genes in the S-CREM2 genome were identified using tRNAscan-SE (Chan and Lowe, 2019). Other ncRNA genes were predicted by searching against the Rfam database^5^ (Yao et al., 2007). Comparative genomic analyses of cluster C cyanophages were conducted and visualized by using Easyfig v2.2.3 (Sullivan et al., 2011).

### Phylogenetic analyses

Phylogenetic analyses of the phycobilin lyase and CP12 genes were conducted using the MEGA 7.0 software package (Kumar et al., 2016). The phycobilin lyase phylogenetic tree was constructed based on amino acid sequences, while the CP12 phylogenetic trees utilized nucleotide sequences. The maximum-likelihood method with the Jones-Taylor-Thornton (JTT) model and the neighbor-joining method with the *p*-distance model were both used in the phylogenetic tree construction with 1,000 bootstrap replicates. Phylogenomic analyses of S-CREM2 and 40 T4-like cyanophages were performed based on the amino acid sequences of 31 core genes. The core genes were identified among the 41 cyanophages using OrthoFinder v2.5.2 (Emms and Kelly, 2015), aligned using MAFFT v7.52 (Katoh et al., 2009), and edited using TrimAI v22.9.0 (Capella-Gutiérrez et al., 2009). The phylogenomic tree was constructed by RAxML v8.2.12 (Stamatakis, 2014) using the maximum-likelihood method with the PROTGAMMAJTT model (bootstrap replicates = 100). Five cyanophage representatives in clusters A, B, and C were selected to analyze the intergenomic similarity by VIRIDIC^6^ (Moraru et al., 2020). All five phages in cluster C, S-CREM2, S-CRM01, S-B68, S-H34, and S-N03, were used in the analysis. Cyanophages S-PM2, S-RSM4, S-SM2, S-SSM7, and P-HM1 were chosen to represent cluster A, while S-ShM2, Syn10, S-RIM8, S-IOM18, and S-RIM2 were selected to act for cluster B.

### Virion protein determination by mass spectrometry analysis

The CsCl-purified phage suspensions were used for the virion protein determination. Virion proteins were digested using the FASP methods procedure described by Wiśniewski et al. (2009). The resulting tryptic peptides were analyzed using a Q Exactive mass spectrometer (Thermo Fisher Scientific, Waltham, MA, USA), coupled to an Easy nLC 1,000 system (Thermo Fisher Scientific) (Michalski et al., 2011). Generated mass spectra were searched against the S-CREM2 genome by using the Mascot2.2 software (Matrix Science, London, UK) to retrieve the data.

### Recruitments of reads from metagenomic data

To estimate the relative abundances and distributional patterns of T4-like cyanophage clusters A, B, and C, fragment recruitment was performed using virome datasets from both marine and freshwater environments. Five representative cyanophages in each cluster used in the intergenomic similarity analysis were selected, and core genes shared among clusters A, B, and C were used for the recruitment analyses. Viromes used in this study include Global Ocean Virome 2.0 (GOV 2.0) (Gregory et al., 2019), Delmarva Estuarine Virome (DEV) (Sun et al., 2021), and Pearl River Estuary Virome (PREV) (Xu et al., 2022; Supplementary Table S1). The GOV 2.0 datasets were downloaded from the iMicrobe website^7^. The DEV datasets were obtained from the NCBI SRA database^8^. The PREV was sourced from the National Omics Data Encyclopedia^9^. Core gene homolog recruitment was conducted using BLASTN, with specific thresholds: an e-value of <1e^−5^, a bit score of >40, a nucleotide identity of >95%, an alignment length of >90 bp, and a coverage of >40% (Mizuno et al., 2016; Martinez-Hernandez et al., 2017). The relative abundances of T4-like cyanophage clusters A, B, and C were normalized by the total recruited nucleotides (kb) per kilobase of core genes per gigabase of metagenome (KPKG) (Martinez-Hernandez et al., 2017).

## Results and discussion

### Morphology and host range of S-CREM2

**Figure 1:**
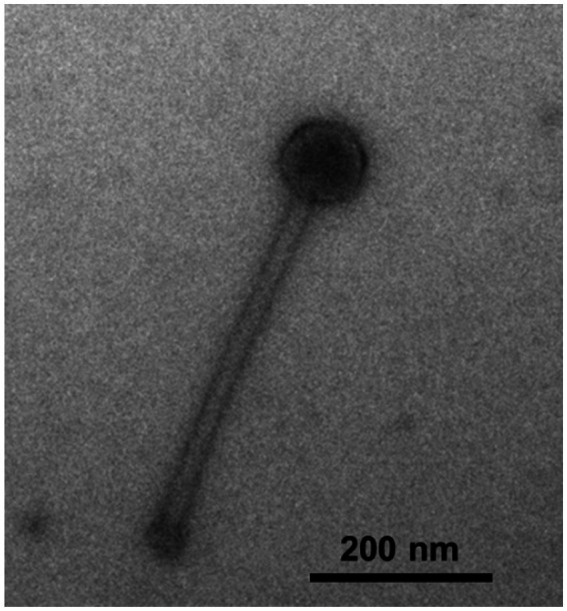
Transmission electron microscopy image of *Synechococcus* phage S-CREM2.

Cyanophage S-CREM2 and its host, *Synechococcus* sp. CRE1902 which is a member of *Synechococcus* subcluster 5.1 clade VI, were both isolated from the surface seawater of the Changjiang River Estuary in July 2019. Transmission electron microscopy observation reveals that S-CREM2 is a myovirus, possessing an isometric capsid (~96 nm in diameter) and an extraordinarily long contractile tail (~418 nm in length) (Figure 1; Supplementary Figure S1). Cyanomyovirus isolates rarely have tails longer than 200 nm (Sullivan et al., 2005; Clokie et al., 2008; Dreher et al., 2011; Sabehi et al., 2012; Xu et al., 2018; Rong et al., 2022; Wang et al., 2022; Zheng et al., 2023). S-CREM2 has the longest tail among the isolated cyanomyoviruses, even the isolated myoviruses, discovered so far. In contrast to the strong cross-infectivity of most cyanomyovirus isolates (Sullivan et al., 2003, 2008; Wang et al., 2022), S-CREM2 exhibits a narrow host range (Table 1). Among the nine *Synechococcus* strains examined in this study, S-CREM2 exclusively infected its original host, failing to cross-infect any other strains, even those in the same phylogenetic clade as the host. Previous studies have also emphasized the limited host range of two cyanomyoviruses, S-CREM1 and S-SZBM1 (Rong et al., 2022; Zheng et al., 2023), which are also isolated from estuarine or coastal environments. More cyanophage isolation would facilitate a better understanding of the picocyanobacteria–phage interactions occurring in the eutrophic marine environment.

**Table 1:** Host range of S-CREM2.

Tested strain	Phylogenetic clade	Isolation source	Result*
CC9311	5.1, clade I	California current	**−**
WH8102	5.1, clade III	Tropical Atlantic	**−**
WH7803	5.1, clade V	Sargasso Sea	**−**
WH7805	5.1, clade VI	Sargasso Sea	**−**
CRE1902	5.1, clade VI	Changjiang River Estuary	**+**
CB0101	5.2, CB4	Chesapeake Bay	**−**
CRE1901	5.2, CB5	Changjiang River Estuary	**−**
CBW1107	Subalpine cluster II	Chesapeake Bay	**−**
CBW1101	Bornholm Sea	Chesapeake Bay	**−**

*: +, infected; −, uninfected.

### Genomic features of S-CREM2

**Figure 2:**
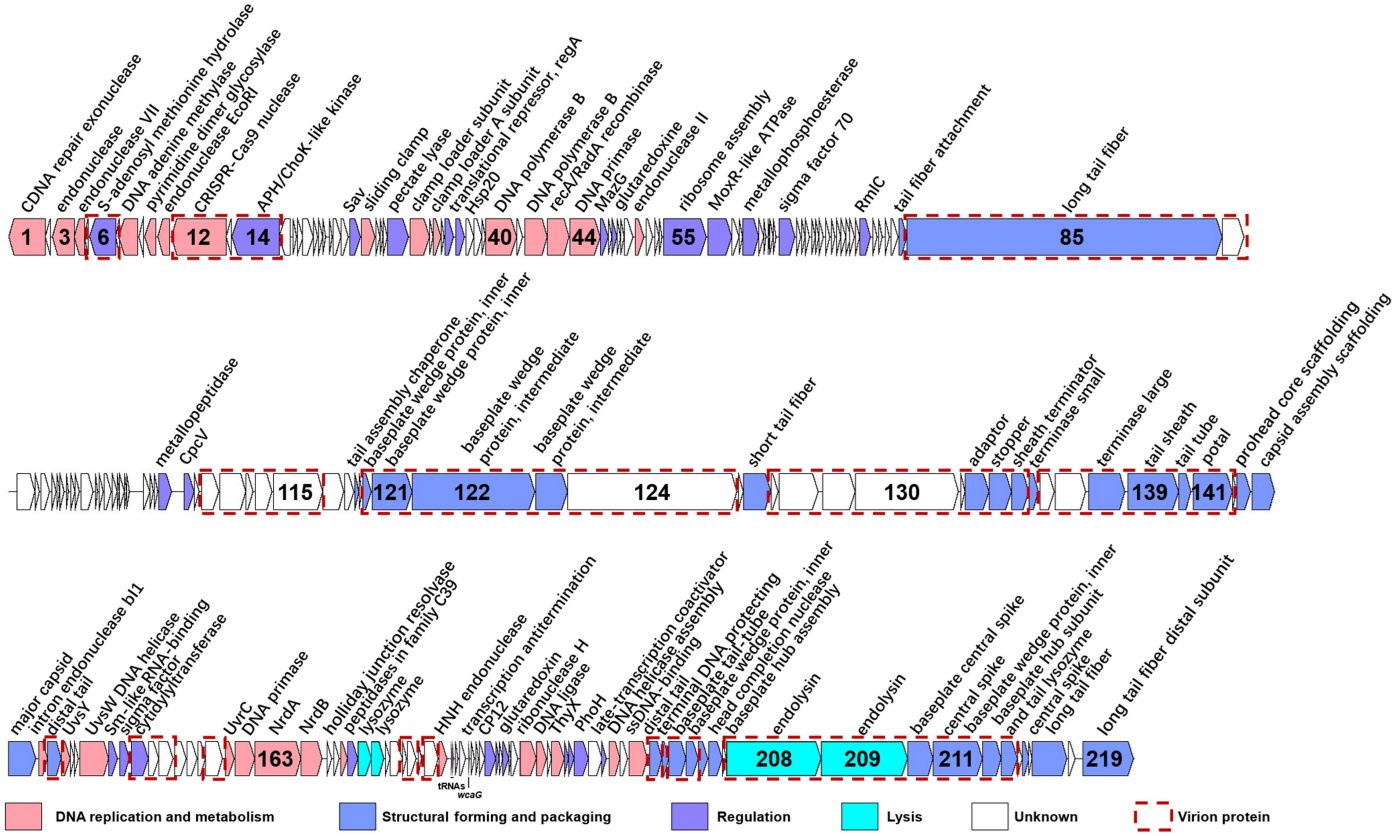
Genome organization of S-CREM2. ORFs with different functions are indicated by different colored arrows, and red dotted boxes represent virion proteins identified by mass spectrometry analysis. The number inside the arrow indicates the ORF number. ncRNA genes are labeled below the ORF bars.

The genome of S-CREM2 was assembled into a circularly permuted, double-stranded DNA molecule with a length of 174,876 bp and a G + C content of 47.92%. A total of 219 ORFs, two tRNA genes, and a *cis*-regulatory RNA gene were predicted in the S-CREM2 genome (Figure 2; Supplementary Table S2). Of the 219 ORFs, 92 were annotated with predicted functions and categorized into four categories, structural formation (32 ORFs), DNA replication and metabolism (29 ORFs), regulation (27 ORFs), and lysis (4 ORFs) (Figure 2; Supplementary Tables S2, S3). The remaining 127 ORFs had unknown functions, with 49 ORFs having no matches in the NR database. A total of 159 ORFs of S-CREM2 showed homology with those of T4-like cyanophages that infect *Prochlorococcus* and *Synechococcus*, which indicates that S-CREM2 is a member of the T4-like cyanophages (Supplementary Table S2).

**Figure 3:**
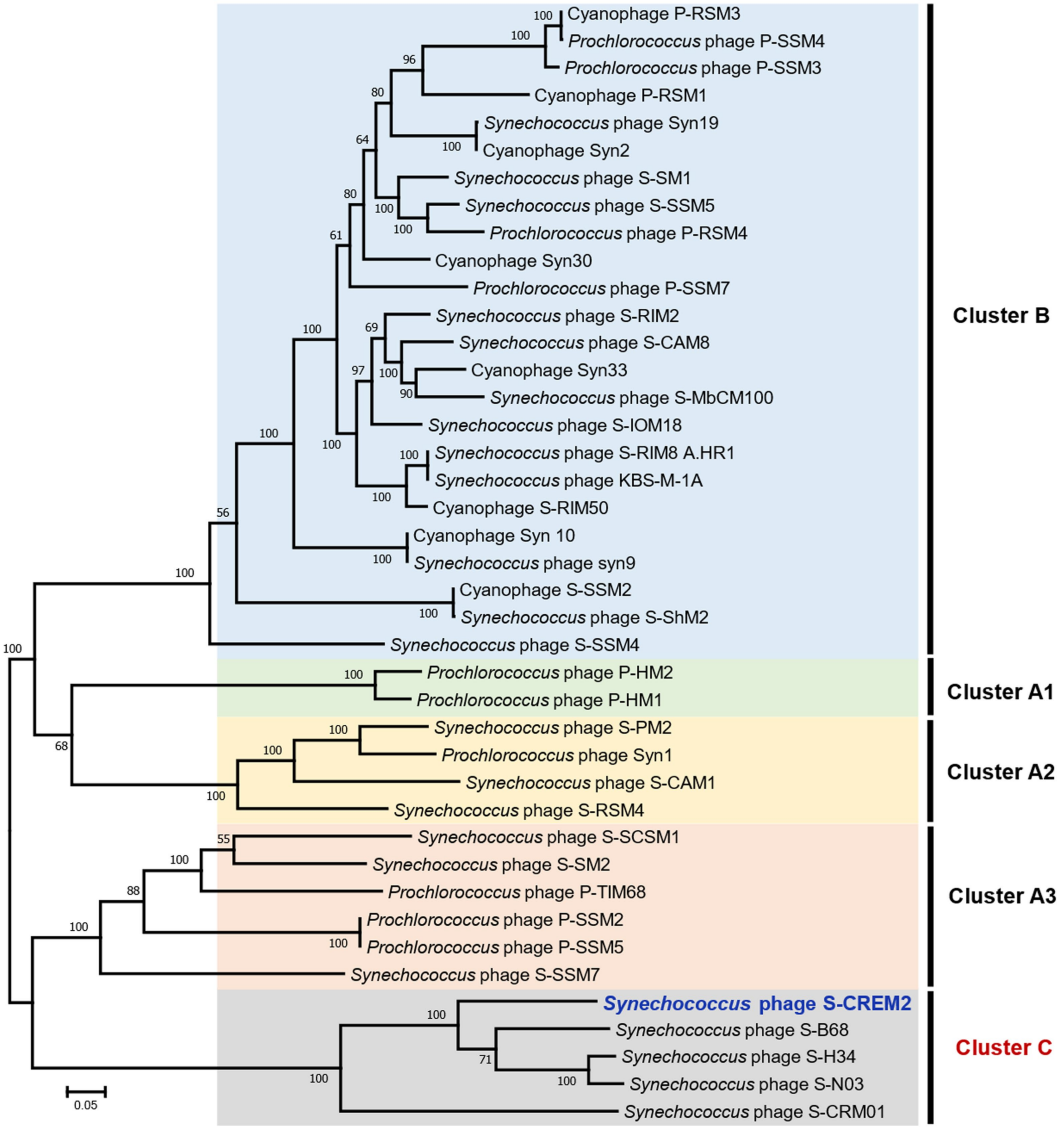
The maximum-likelihood phylogenomic tree based on the 31 core genes among S-CREM2 and 40 T4-like cyanophages. Bootstrap values are calculated based on 100 replicates. The 31 core genes contain 11 DNA replication-related genes, 13 structure-related genes, four AMGs, and three hypothetical genes.

To investigate the phylogenetic relationship between S-CREM2 and other T4-like cyanophages, a set of 31 core genes were identified in S-CREM2 (Supplementary Table S4) and 40 referenced T4-like cyanophages. Phylogenomic analysis based on these 31 core genes revealed that S-CREM2 clustered with *Synechococcus* phages S-B68, S-N03, S-H34, and S-CRM01 and formed a discrete clade, which is divergent from the well-characterized clusters A and B proposed by Ignacio-Espinoza and Sullivan (2012) (Figure 3). The new clade encompassing S-CREM2, S-B68, S-N03, S-H34, and S-CRM01 were named as cluster C, in which the marine phage strains S-CREM2, S-B68, S-N03, and S-H34 exhibit closer phylogenetic relationship with each other and are relatively distant from the freshwater strain S-CRM01. In addition, the G + C contents of marine strains in cluster C (47.9–51.7%) are much higher than that of the freshwater strain S-CRM01 (39.7%) (Table 2) and those of cluster A (37.8–43%) and B (36.7–42.2%) (Jiang et al., 2020). A total of 150 ORFs in S-CREM2 showed homology with the T4-like cluster C cyanophages and 46 of them were exclusively homologous to cluster C. Within cluster C, S-CREM2 shares the largest number of homologous genes (144) with S-H34. In addition, S-CREM2 has 137, 135, and 78 ORFs homologous with S-N03, S-B68, and S-CRM01, respectively (Supplementary Figure S2; Supplementary Table S2).

**Table 2:** Genomic and morphology features of the T4-like cyanophages in cluster C.

Cyanophage	Isolation location	Host strain used for isolation	Host phylogenetic clade	Genome size (kb)	G + C (%)	tRNA no.	*Cis*-regulatory RNA no.	Capsid size (nm)	Tail size (nm)	Accession no.	Reference
S-CREM2	Changjiang River estuary	*Synechococcus* CRE1902	5.1-VI	174.9	47.9	2	1	96	418	OR473000	This study
S-B68	Bohai Sea	*Synechococcus* WH7803	5.1-V	164	51.7	4	1	–	–	MK016664.1	Huang et al. (2020)
S-N03	Yellow Sea	*Synechococcus* MW02	5.1-IX	167.1	50.1	1	1	97	138	MT162466.1	Jiang et al. (2020)
S-H34	Yellow Sea	*Synechococcus* MW02	5.1-IX	167	50.1	5	2	98	129	MT162467.2	Jiang et al. (2020)
S-CRM01	Copco Reservoir on the Klamath River, Northern California	*Synechococcus* LC16	5.2-*Cyanobium gracile*	178.6	39.7	33	0	85–100	140–170	HQ615693.1	Dreher et al. (2011)

Five cyanophages in each T4-like cluster were selected as representatives to calculate nucleotide-based intergenomic similarities. The nucleotide similarities between cluster C cyanophages and representatives in clusters A and B (4.4–6.8%) are much lower than those within cluster C (11.4–68%) (Supplementary Figure S3). S-CREM2 showed nucleotide similarities of 11.4–32.8% with the other four cyanophages in cluster C, with the highest similarity observed with S-B68 and the lowest with S-CRM01. Following the genus-level classification criteria in phage taxonomy, the nucleotide similarity less than 50% of the whole genome is indicative of different genera (Adriaenssens and Brister, 2017). Thus, we propose classifying S-CREM2 as a representative of a new viral genus.

### DNA replication and metabolism genes of S-CREM2

A total of 29 ORFs encode genes related to DNA replication and metabolism in the S-CREM2 genome, including DNA polymerase, helicase, primase, ligase, various endonucleases, and enzymes involved in nucleotide metabolism and DNA damage repair. Nucleotide metabolism genes encoded in the S-CREM2 genome include *nrdA* (ORF163), *nrdB* (ORF164), *thyX* (ORF192), and DNA adenine methylase gene (ORF8). Specifically, *nrdA*, *nrdB*, and *thyX* can provide DNA monomers for viral replication (Myllykallio et al., 2002; Gon et al., 2006; Koehn and Kohen, 2010). DNA adenine methylase, which is involved in the process of nucleotide methylation, plays an important role in enhancing DNA stability (Miller et al., 2003). Among the DNA damage repair genes, the product of ORF10 (putative pyrimidine dimer DNA glycosylase) may function as a base-cutting repair protein, thereby reducing the occurrence of pyrimidine dimer formation caused by UV damage (Grafstrom et al., 1982; Walker et al., 2006), while the S-CREM2 putative UvsY (ORF1) and CDNA repair exonuclease SbcCD ATPase subunit (ORF148) may be able to remove mutated bases and nucleotide fragments (Wilson and Murray, 1991). The expression of these genes may be crucial in maintaining accurate transcriptional translation when the virus or host is subjected to external damage (Kemp and Hu, 2017). Additionally, a CRISPR-Cas9 nuclease (ORF12) gene is predicted in the S-CREM2 genome. Bacterial CRISPR-Cas9 nuclease is associated with chromosome rearrangement and genotoxicity, and it functions as a component of the adaptive immune system, which serves to defend against viral infection by degrading DNA originating from invading viruses or other foreign sources (Cui et al., 2020). The S-CREM2 CRISPR-Cas9 nuclease gene may be acquired from cyanobacterial hosts through horizontal gene transfer.

Among the 29 DNA replication and metabolism ORFs predicted in the S-CREM2 genome, 27 ORFs are homologous to those predicted in other T4-like cluster C cyanophages, with the highest amino acid identity for each gene ranging from 32.7 to 94.6% and averaged at 73.1%. Additionally, 28 out of the 29 DNA replication and metabolism ORFs showed homology with genes predicted in T4-like clusters A and B cyanophages, with the highest amino acid identity of each gene ranging from 29.3 to 81.4% and averaged at 54.6% (Supplementary Figure S4A).

### Structural genes of S-CREM2

**Figure 4:**
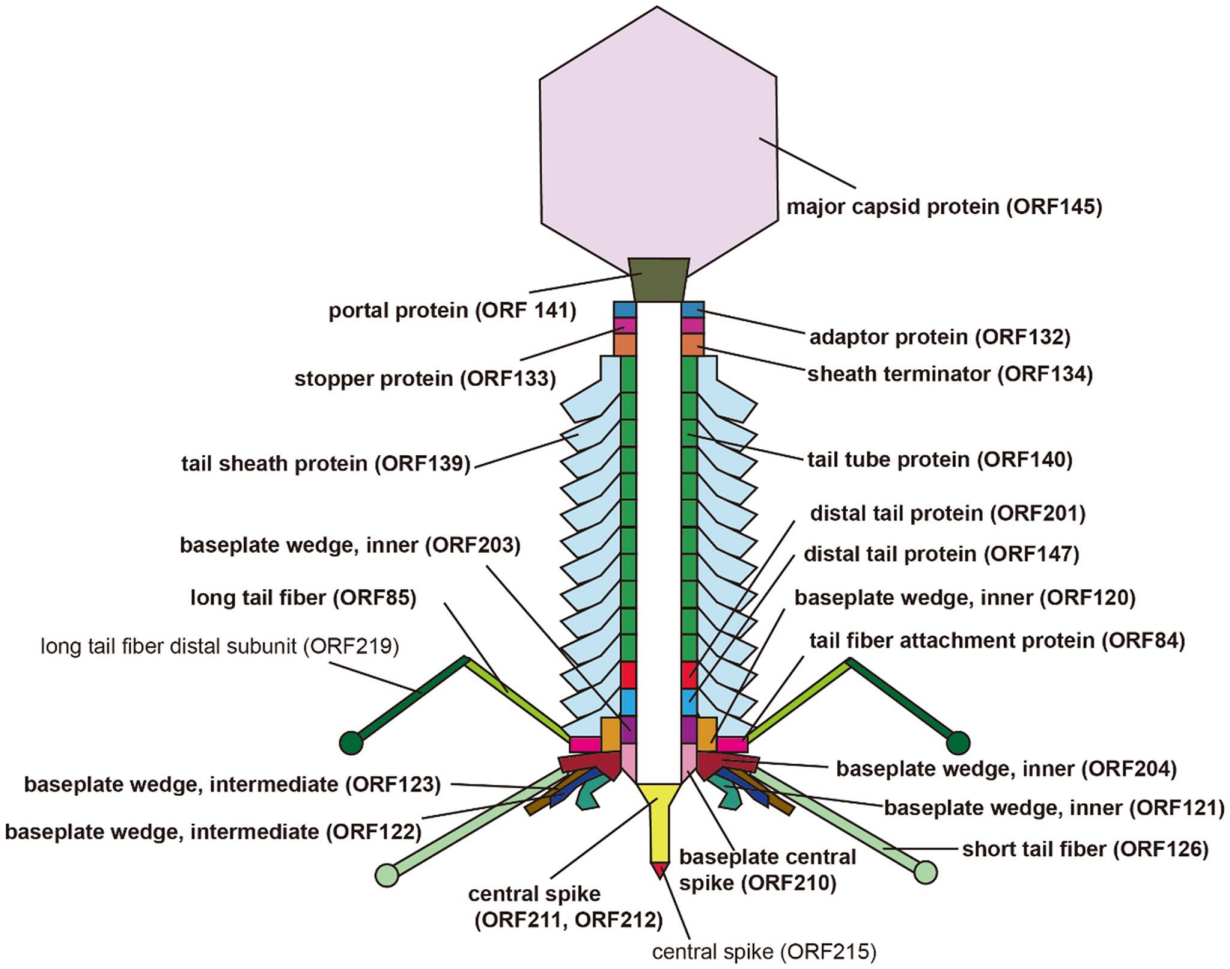
Predicted architecture of the S-CREM2 virion. Bold fonts indicate structural proteins detected by mass spectrometry analysis.

A total of 32 ORFs were predicted to encode structural proteins in the S-CREM2 genome, including terminase large subunit, terminase small subunit, portal protein, adaptor, stopper, sheath terminator, capsid-related proteins, and tail-related proteins (Figure 2; Supplementary Tables S2, S3), 24 of which were detected in the virion proteome by mass spectrometry analysis (Table 3). Of the 32 structural ORFs, 31 show homology to ORFs predicted in other cluster C cyanophages, with the highest amino acid identity for each gene ranging from 30.6 to 92.9% and averaged at 64.6% (Supplementary Figure S4B). The S-CREM2 ORF219, encoding a long tail fiber distal subunit, shows no homology with any cyanophages, but is homologous to genes predicted in heterotrophic bacteria, other bacteriophages, and *Ostreococcus lucimarinus* viruses, with amino acid identities ranging from 26.5 to 67% (Supplementary Table S2). Of the 32 S-CREM2 structure-related ORFs, 25 are homologous with those predicted in the T4-like cluster A and B cyanophages, with the highest amino acid identity of each gene ranging from 26.8 to 70.5% and averaged at 46.9% (Supplementary Figure S4B). It is worth noting that the S-CREM2 ORFs involved in structure formation exhibit a lower degree of conservation compared to ORFs related to DNA replication and metabolism (Supplementary Figure S4). Notably, six tail-related ORFs of S-CREM2 are exclusively homologous to genes predicted in the T4-like cluster C cyanophages. By comparing the S-CREM2 structural proteins involved in the virion formation with those of the T4 phage, the overall architecture of the S-CREM2 virion was predicted (Figure 4). Twenty-three proteins were mapped to the virion structure, with 21 detected in the virion proteome (Figure 4). Most of the structural proteins of S-CREM2 highly resemble those of the T4 phage. However, the adopter and the long tail fiber are different from those of the T4 phage and show homology with those of *Escherichia* phage vB_EcoP_SU10 and *Escherichia* phage K1F, respectively (Supplementary Table S5).

**Table 3:** The virion proteome of S-CREM2.

ORF	Strand	Left	Right	Putative function	No. of unique peptides	Sequence coverage (%)
**Structural protein**						
84	+	42165	42554	Tail fiber attachment	4	21.7
85	+	42559	57474	Long tail fiber	286	29.2
120	+	75282	75689	Baseplate wedge protein, inner	11	36.3
121	+	75723	77597	Baseplate wedge protein, inner	113	42
122	+	77594	83407	Baseplate wedge protein, intermediate	91	27.2
123	+	83440	84939	Baseplate wedge protein, intermediate	55	38.9
126	+	93309	94577	Short tail fiber	6	10.7
132	+	103792	104919	Adaptor	20	21.1
133	+	104922	105965	Stopper	9	22.8
134	+	105994	106821	Sheath terminator	28	36
139	+	111494	113878	Tail sheath protein	256	39.9
140	+	113911	114510	Tail tube protein	73	46.7
141	+	114599	116422	Portal protein	53	34.3
143	+	116656	117297	Prohead core protein protease	4	9.9
144	+	117378	118436	Capsid assembly scaffolding protein	7	16.2
145	+	118481	119851	Major capsid protein	165	38.8
147	+	120464	121129	Distal tail protein	15	36.7
201	+	150585	151256	Distal tail protein	16	29.6
203	+	151597	152421	Baseplate wedge protein, inner	14	32.5
204	+	152421	153053	Baseplate wedge protein, inner	11	31.9
210	+	163557	164837	Baseplate central spike	25	43.9
211	+	164834	167272	Central spike	40	33
212	+	167269	168228	Baseplate wedge protein, inner	14	29.2
213	+	168230	169045	Baseplate hub subunit and tail lysozyme	2	7.8
**Regulatory protein**						
6	–	5081	3837	*S*-adenosyl methionine hydrolase	19	26.3
14	–	12846	10579	APH/ChoK-like kinase	21	23.6
154	+	124608	125468	Cytidylyltransferase	3	11.2
**DNA replication and metabolism protein**						
12	–	10313	7704	CRISPR-Cas9 nuclease	28	27.4
**Lysis protein**						
208	+	154483	159180	Endolysin	73	24.9
209	+	159184	163554	Endolysin	71	29.5
**Hypothetical protein**						
13	–	10546	10310	Hypothetical protein	1	11.5
15	–	13331	12843	Hypothetical protein	4	14.8
17	+	13692	13934	Hypothetical protein	1	7.5
86	+	57474	58481	Hypothetical protein	1	3
111	+	67610	68431	Hypothetical protein	8	19.1
112	+	68500	69726	Hypothetical protein	76	33.1
114	+	70167	70994	Hypothetical protein	7	15.6
115	+	71020	73407	Hypothetical protein	56	30.2
116	+	73407	74360	Hypothetical protein	9	16.1
124	+	84976	92982	Hypothetical protein	450	46
127	+	94606	94944	Hypothetical protein	1	8
128	+	94985	96814	Hypothetical protein	93	31.5
129	+	97032	98582	Hypothetical protein	19	22.9
130	+	98589	103511	Hypothetical protein	24	8.7
136	+	107301	108014	Hypothetical protein	8	24.1
137	+	108049	109521	Hypothetical protein	4	4.1
155	+	125491	126012	Hypothetical protein	5	14.5
156	+	126012	126725	Hypothetical protein	6	15.2
160	+	128342	129235	Hypothetical protein	7	13.1
165	+	134445	134699	Hypothetical protein	1	8.3
174	+	138460	138900	Hypothetical protein	7	33.6
176	+	139308	140018	Hypothetical protein	20	39.4

### Virion-associated proteins of S-CREM2

A total of 52 S-CREM2-encoded proteins were detected in the virion proteome by mass spectrometry analysis, including 24 structural proteins, six non-structural proteins, and 22 proteins with unknown function (Figure 2; Table 3). Notably, among the 22 protein genes with unknown function, 13 are located in the genome region (ORF84–145) primarily associated with structural genes and may also encode structural proteins, indicating distinctive proteins contributing to the formation of the unique virion, which has an extraordinarily long tail. The six non-structural proteins include S-adenosyl methionine (SAM) hydrolase (ORF6), APH/ChoK-like kinase (ORF14), cytidylyltransferase (ORF154), CRISPR-Cas9 nuclease (ORF12), and two endolysins (ORF208, 209) and may be encapsulated within the capsid as virion-associated proteins.

SAM hydrolase is essential for the degradation of S-adenosine methionine (SAM) (Jerlström Hultqvist et al., 2018). SAM serves as a crucial methyl donor for methyltransferases that function on nucleic acids, proteins, and lipids in bacteria cells (Loenen, 2006). As a defense mechanism, bacteria employ SAM to differentiate their own DNA from that of foreign invaders (Wilson and Murray, 1991). It is reported that the phage-encoded SAM hydrolase can degrade SAM, switching off the bacterial defense (Jerlström et al., 2018; Guo et al., 2021). The entry of the phage-encoded SAM hydrolase into the host cell upon infection may protect the phage genomic DNA from attacks of the host restriction-modification systems. VAPKs are common in enveloped viruses infecting animals and plants but are rarely discovered in phages (Hui, 2002). Recently, protein kinase-like proteins have been continuously detected in the cyanophage virion proteomes (Dreher et al., 2011; Sabehi et al., 2012; Xu et al., 2018) and are speculated to regulate host bioprocesses by phosphorylating specific substrates like serine, threonine, or tyrosine residues of proteins, aminoglycosides, and choline. The putative APH/ChoK-like kinase detected in the S-CREM2 virions is homologous and shares an amino acid identity of 32% with the putative protein kinase detected in the S-CRM01 virions. In prokaryotes, APHs phosphorylate and inactivate aminoglycoside antibiotics (Wright and Thompson, 1999), ChoKs facilitate the formation of phosphorylcholine and play an important role in phosphorylcholine-associated lipopolysaccharide modifications on cell surface and cell stress (Thomsen et al., 2003). The S-CREM2 APH/ChoK-like kinase may influence antibiotic resistance and the stress tolerance of the host cells (Wright and Thompson, 1999; Thomsen et al., 2003). Cytidyltransferase is a homolog of NMNAT. Previous studies have speculated that phage-encoded NMNAT may regulate host metabolism by affecting NAD^+^ levels in the cell and promote the production of phage progeny (Raffaelli et al., 1997, 1999, 2001; Wang et al., 2022). The frequent detection of protein or small molecule kinases and cytidyltransferases in the virion proteomes of cyanophages suggests that these proteins may be carried by the virion and able to enter the host cells to create an optimized intracellular environment that fosters phage replication at the initial stage of phage infection. However, it is also possible that these phage-encoded regulatory proteins are highly expressed during phage infection and were not separated from virions in the CsCl purification. Further efforts are needed to verify their presence in the phage virions.

### Limited and unique AMGs in T4-like cluster C cyanophages

**Figure 5:**
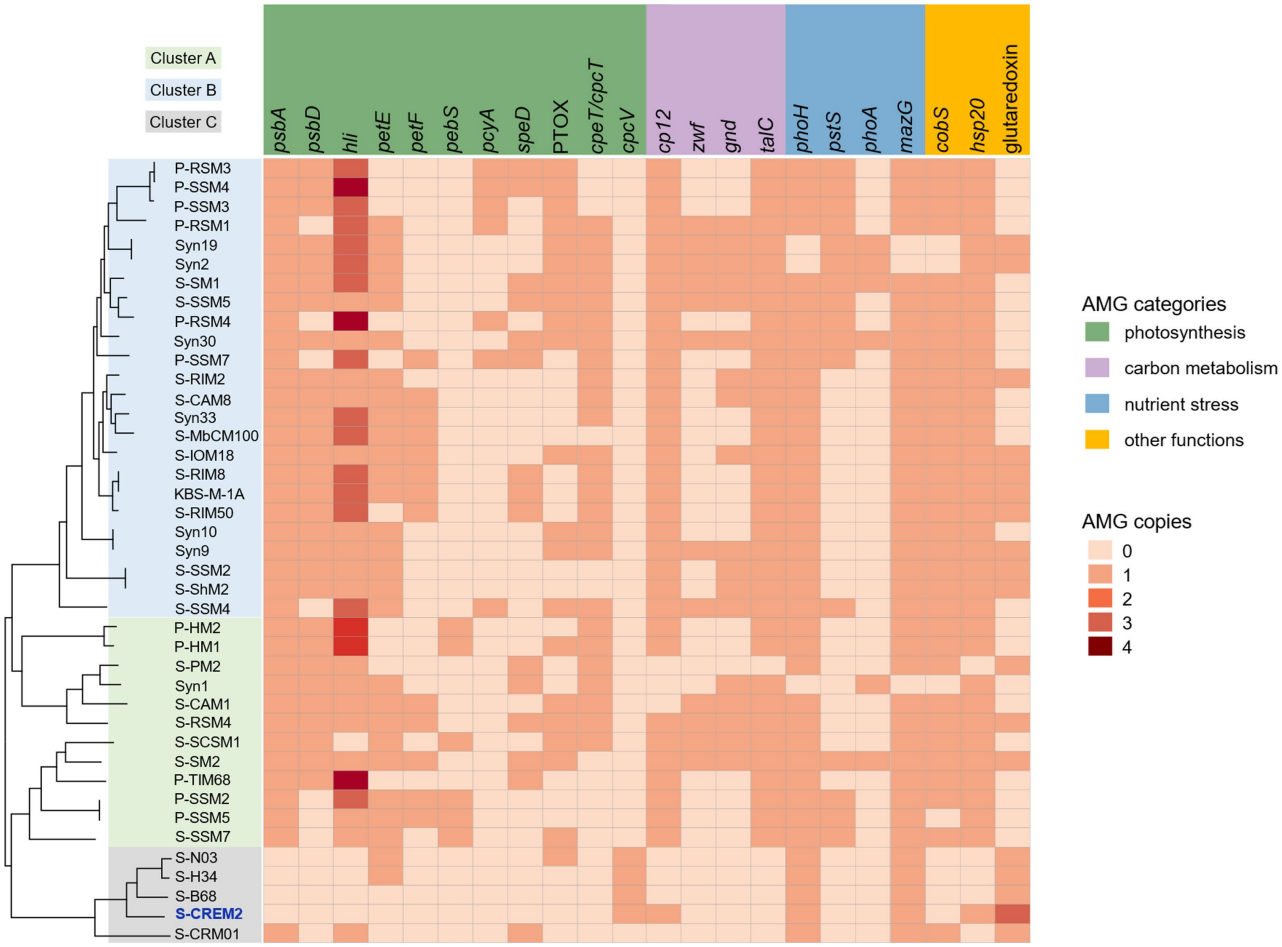
Comparative analysis of the AMGs in different T4-like cyanomyovirus clusters. Phylogenomic tree of S-CREM2 and 40 T4-like cyanophage based on 31 core genes. Colored boxes on the left signify T4-like cyanomyovirus clusters.

In contrast to the numerous and diverse AMGs identified in T4-like cyanophages of clusters A and B, only a limited number of AMGs were predicted in the T4-like cluster C cyanophages (Figure 5). Only the freshwater strain, S-CRM01, contains the six most commonly found AMGs, *psbA*, *hli*, *phoH*, *mazG*, *cobS*, and *hsp20,* in T4-like cyanophages of clusters A and B (Ignacio-Espinoza and Sullivan, 2012; Jiang et al., 2020). While, four marine strains in cluster C, S-CREM2, S-H34, S-N03, and S-B68, only encode *phoH*, *mazG*, and *hsp20* (Figure 5).

**Figure 6:**
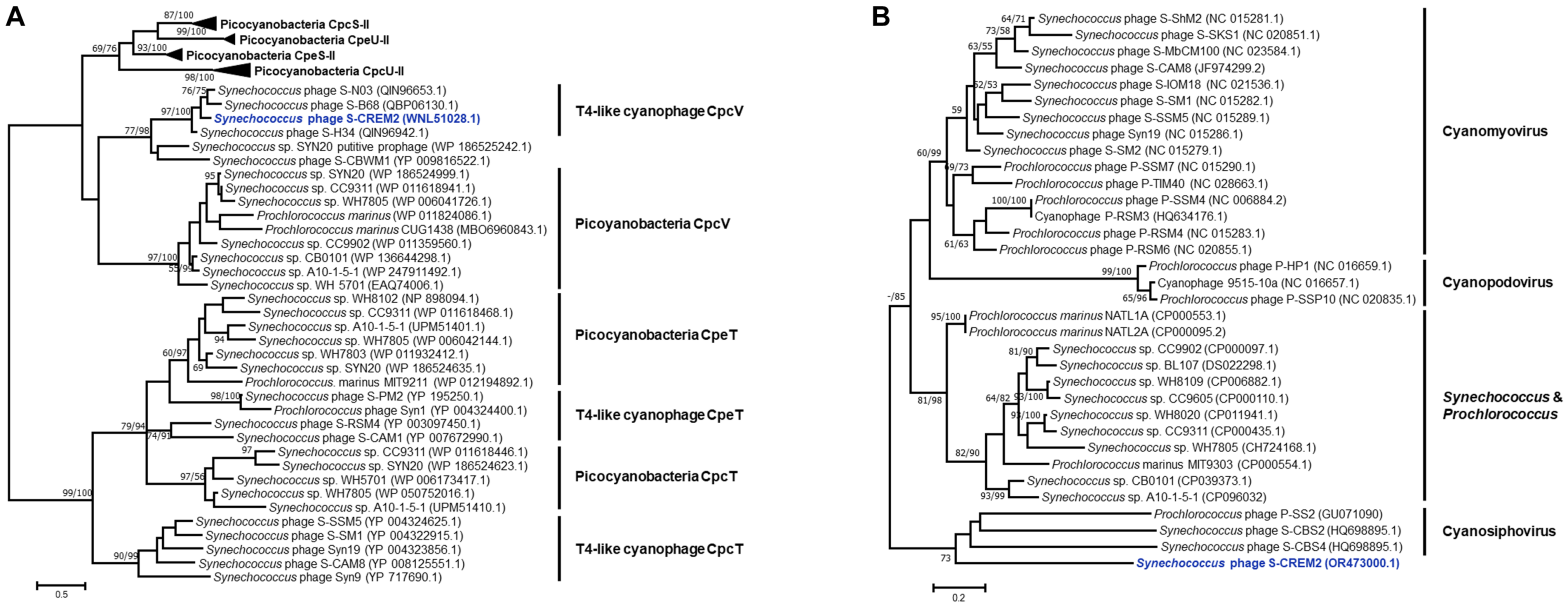
Maximum-likelihood phylogenetic trees of phycobilin lyase genes **(A)** and *cp12*s **(B)** from picocyanobacteria and cyanophages. The phylogenetic analyses of the phycobilin lyase genes were performed based on the amino acid sequences, while the *cp12* trees were constructed using nucleotide sequences. Numbers near each branch node represent the bootstrap values (maximum-likelihood/neighbor-joining, ML/NJ) of ≥50%. The bootstrap replicates = 1,000.

Tough lacking the commonly found photosynthesis genes *psbA* and *hli*, four marine cyanophage strains in cluster C encode an S-type phycobilin lyase gene, *cpcV* (Figure 5). Phycobilin lyases catalyze the covalent ligation between phycobilin chromophores and phycobiliproteins at specific binding sites, facilitating the synthesis of phycobilisome (Bretaudeau et al., 2013). The expression of phage *cpcV*s may assist the light absorption in infected host cells, providing energy for phage replication (Six et al., 2007; Xu et al., 2018). Phycobilin lyase genes are common in T4-like cyanophages of clusters A and B. However, all of the phycobilin lyase genes in clusters A and B are T-type, *cpeT* or *cpcT*. The *cpcV* is only found in T4-like cluster C cyanophages and S-CBWM1 (Xu et al., 2018). Phylogenetic analysis revealed that *cpcT* and *cpeT* of cyanophages in clusters A and B grouped into a stable branch with those of picocyanobacteria. The *cpcV* of cluster C cyanophages formed an individual clade with those of S-CBWM1 and a putative prophage of *Synechococcus* sp. SYN20, but did not cluster with any host-derived *cpcV* (Figure 6A). The discovery of more *cpcV* homologs from both cyanophage and cyanobacteria would facilitate the illustration of the evolutionary source and trajectory of the cyanophage *cpcV*s in future studies.

In addition, the S-CREM2 genome carries a *cp12* that is related to carbon metabolism. As a Calvin cycle inhibitor, the phage-encoded CP12 was proposed to redirect the host carbon flow from the Calvin cycle to the pentose phosphate pathway, resulting in ATP, NADPH, and pentose accumulation that are favorable for phage dNTP biosynthesis (Thompson et al., 2011). The phylogeny of *cp12* revealed that *cp12* homologs from marine T4-like cyanophages of clusters A and B and cyanopodoviruses both grouped with those of marine picocyanobacteria, indicating that cyanophages may acquire *cp12* from their hosts. However, the S-CREM2 *cp12* clustered with those of three cyanosiphoviruses and formed a very deep branch (Figure 6B), which suggests that the S-CREM2 *cp12* evolves from a different origin or has experienced a divergent evolutionary trajectory from those of T4-like cyanophages in clusters A and B.

### Distinct ncRNA profiles between the marine and freshwater phage strains in cluster C

Three ncRNA genes were identified in the S-CREM2 genome, including two tRNA genes and a *cis*-regulatory RNA gene (*wcaG*) (Figure 2). The numbers of tRNA genes vary greatly among cyanophages in T4-like cluster C (Table 2). The marine phages in cluster C contain no more than five tRNA genes, while the freshwater strain, S-CRM01, contains 33 tRNA genes which cover all 20-amino-acid specificities. Notably, the G + C content of S-CRM01 (39.7%) is much lower than those of the marine phage strains in cluster C (47.9–51.7%). While, the G + C contents of their hosts are the opposite. The G + C contents of *Synechococcus* CRE1902 and WH7803, which are hosts of S-CREM2 and S-B68, are 57.4 and 60.2%. The G + C content of the S-CRM01 host, *Synechococcus* LC16, is not available. *Synechococcus* LC16 is a member of the *Cyanobium gracile* cluster. Since the G + C contents of cyanobacteria in the same phylogenetic clade are usually similar, the G + C content of *Synechococcus* LC16 can be estimated from that of the type strain in the *Cyanobium gracile* cluster, *Synechococcus* PCC6307, which is 68.5% and much higher than those of *Synechococcus* CRE1902 and WH7803. It is speculated that phages carry tRNAs to overcome the codon usage difference from its hosts (Enav et al., 2012). The large difference in tRNA number between the S-CRM01 and the marine phages in cluster C can be illustrated by the larger discrepancy of G + C contents between S-CRM01 and its host than those between the marine phage strains and their hosts. A *cis*-regulatory RNA gene, *wcaG,* was also predicted in genomes of the other three marine phages of cluster C. S-H34 also contains an extra *glnA.* However, no *cis*-regulatory elements were found in the S-CRM01 genome (Table 2). In prokaryotes, the *cis*-regulatory RNA *wcaG* acts as a regulator of exopolysaccharide production-related genes, *glnA* regulates gene expressions related to nitrogen metabolism (Weinberg et al., 2010). Phage *cis*-regulatory RNAs may also play similar roles in altering host metabolisms during infection. Different compositions of tRNA and *cis*-regulatory RNA genes between the marine and freshwater phages in cluster C may reflect their different modes of coping with their hosts and habitats.

### Ecological distribution of cluster C cyanophages

**Figure 7:**
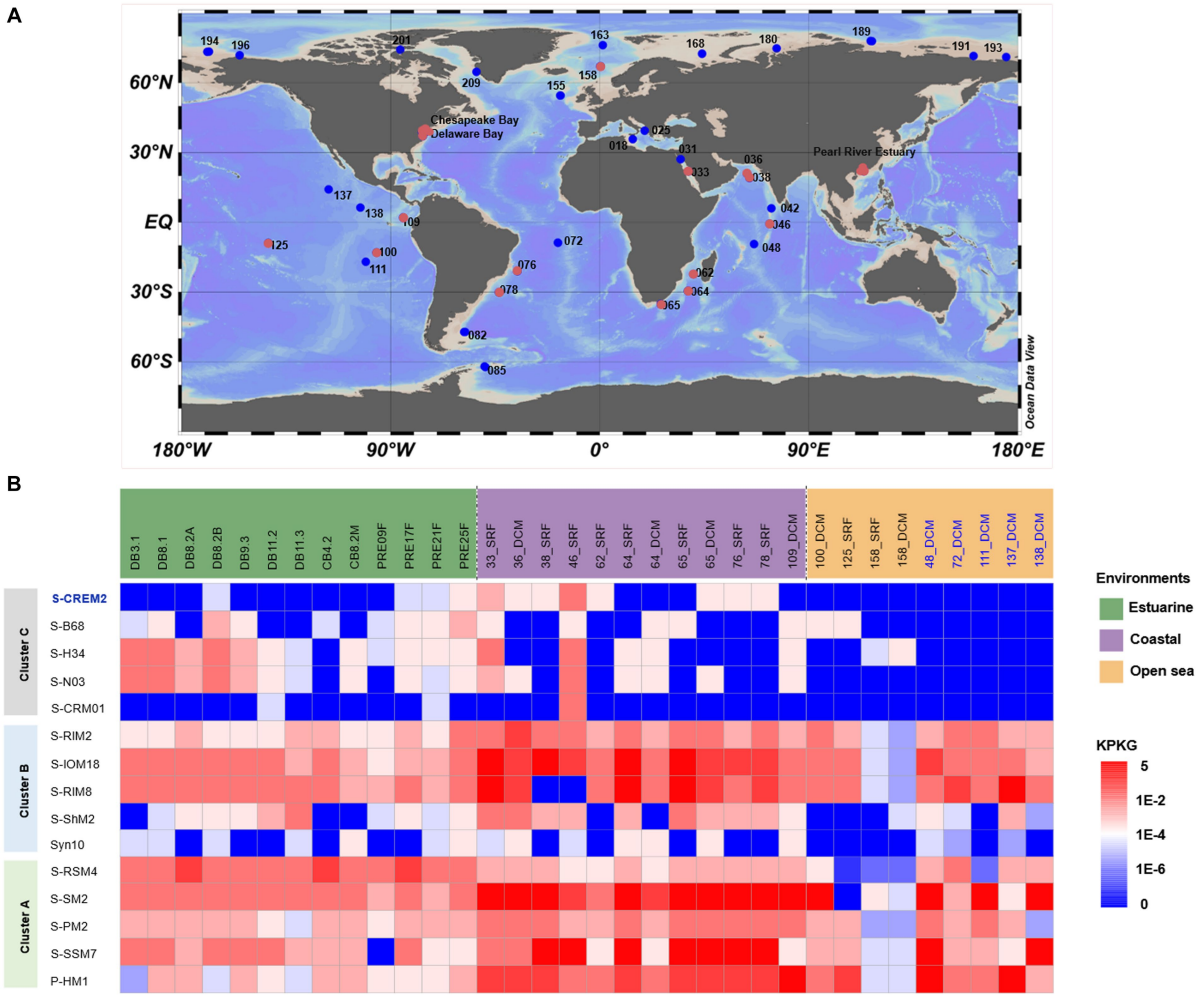
Comparison of environmental distribution of T4-like clusters A, B, and C-like cyanomyoviruses. **(A)** Location of publicly available viriomes used for the distributional analyses of the T4-like cyanophages. Red and blue dots indicate the presence and absence of the cluster C-like cyanophages; **(B)** The relative abundances of five representative strains of cyanophages in each cluster in metagenome databases. The results for 29 viromes containing cluster C-like cyanophages were shown, while those for additional five open sea viromes were also exhibited to better demonstrate the distributional pattern of cyanophages in clusters A and B in the open ocean. Relative abundance was normalized by KPKG.

The distribution and relative abundance of the T4-like cluster C cyanophages in the marine environment were investigated by metagenomic fragment recruitment analyses and compared with those of T4-like cluster A and B cyanophages. Among the 120 viromes employed for recruitment analyses, the cluster C-like cyanophages were detected in 29 viromes retrieved from various ecosystems, including temperate and subtropic estuaries, diverse coastal regions, and open oceans in both tropic and polar regions (Figure 7A). The five cluster C-like cyanophages are widespread in estuarine and coastal regions. Specially, the S-CREM2-like cyanophages are more abundant in coastal environments, the S-H34, S-N03, and S-B68-like cyanophages are more prevalent in estuarine environments. Whereas, ORF homologs of the freshwater strain, S-CRM01, are rarely detected in marine ecosystems (Figure 7B). However, the residence of these five cluster C-like cyanophages in the open sea is quite limited. Only four out of 18 open sea viromes used in this study exhibit the presence of cluster C-like cyanophages (Figure 7). The distributional pattern of the cluster C-like cyanophages is congruent with those of their hosts, *Synechococcus* subcluster 5.1 clade V, VI, and IX (Table 2), which also thrive in the estuarine and coastal environments but are rarely observed in the open sea (Xia et al., 2015; Sohm et al., 2016). The cluster A and B-like cyanophages are prevalent across various marine ecosystems. Despite consistently lower relative abundance compared to specific cluster A and B-like members in various marine ecosystems, specific members of cluster C-like cyanophages exhibit comparable or even higher relative abundances in certain estuarine regions (Figure 7B). This suggests that cluster C-like cyanophages play important ecological roles in the estuarine environment, which has been previously overlooked due to a lack of awareness regarding their existence.

## Conclusion

Cyanophage S-CREM2 represents a new viral genus. The discovery of S-CREM2 refreshes our knowledge of the tail length of cyanomyoviruses and leads to the establishment of a new T4-like cyanophage clade, cluster C. Much less and unique AMGs and various virion-associated regulatory proteins of S-CREM2 may drive different phage–host interactions from those of clusters A and B cyanophages. The T4-like cluster C cyanophages are widespread in the estuarine and coastal environment. Specific members of this cluster may play important roles in certain estuarine ecosystems due to their equivalent or even higher relative abundance compared to cyanophages of clusters A and B. The isolation of S-CREM2 and establishment of the T4-like cluster C cyanophages provide new insights into the phage diversity, evolution, and phage–host interactions in the marine environment.

## Data availability statement

The datasets presented in this study can be found in online repositories. The names of the repository/repositories and accession number(s) can be found in the article/Supplementary material.

## Author contributions

YL: Data curation, Formal analysis, Visualization, Writing – original draft. XM: Formal analysis, Methodology, Writing – original draft. HZ: Data curation, Methodology, Writing – review & editing. LC: Writing – review & editing. SW: Writing – original draft, Visualization. MH: Writing – original draft, Visualization. JH: Writing – original draft, Visualization. YH: Writing – original draft, Visualization. CG: Writing – original draft, Visualization. JL: Supervision, Writing – review & editing. FC: Funding acquisition, Supervision, Writing – review & editing. YX: Funding acquisition, Supervision, Writing – review & editing.

## Footnotes

^1^http://exon.gatech.edu/GeneMark/

^2^http://rast.nmpdr.org/

^3^http://metagene.nig.ac.jp/

^4^https://toolkit.tuebingen.mpg.de/tools/hhpred

^5^https://rfam.xfam.org/

^6^http://rhea.icbm.uni-oldenburg.de/VIRIDIC/

^7^http://data.imicrobe.us/

^8^https://www.ncbi.nlm.nih.gov/sra/

^9^https://www.biosino.org/node/

## References

[ref1] Adriaenssens E. M. Brister J. R. (2017). How to name and classify your phage: an informal guide. Viruses 9:70. doi: 10.3390/v9040070, PMID: 28368359 PMC5408676

[ref2] Besemer J. Borodovsky M. (2005). GeneMark: web software for gene finding in prokaryotes, eukaryotes and viruses. Nucleic Acids Res. 33, W451–W454. doi: 10.1093/nar/gki487, PMID: 15980510 PMC1160247

[ref3] Bretaudeau A. Coste F. Humily F. Garczarek L. le Corguillé G. Six C.. (2013). CyanoLyase: a database of phycobilin lyase sequences, motifs and functions. Nucleic Acids Res. 41, D396–D401. doi: 10.1093/nar/gks1091, PMID: 23175607 PMC3531064

[ref4] Brettin T. Davis J. J. Disz T. Edwards R. A. Gerdes S. Olsen G. J.. (2015). RASTtk: a modular and extensible implementation of the RAST algorithm for building custom annotation pipelines and annotating batches of genomes. Sci. Rep. 5:8365. doi: 10.1038/srep08365, PMID: 25666585 PMC4322359

[ref5] Capella-Gutiérrez S. Silla-Martínez J. M. Gabaldón T. (2009). trimAl: a tool for automated alignment trimming in large-scale phylogenetic analyses. Bioinformatics 25, 1972–1973. doi: 10.1093/bioinformatics/btp348, PMID: 19505945 PMC2712344

[ref6] Chan P. P. Lowe T. M. (2019). tRNAscan-SE: searching for tRNA genes in genomic sequences. Methods Mol. Biol. 1962, 1–14. doi: 10.1007/978-1-4939-9173-0_1, PMID: 31020551 PMC6768409

[ref7] Chen F. Wang K. Stewart J. Belas R. (2006). Induction of multiple prophages from a marine bacterium: a genomic approach. Appl. Environ. Microbiol. 72, 4995–5001. doi: 10.1128/AEM.00056-06, PMID: 16820498 PMC1489376

[ref9] Clokie M. R. J. Thalassinos K. Boulanger P. Slade S. E. Stoilova-McPhie S. Cane M.. (2008). A proteomic approach to the identification of the major virion structural proteins of the marine cyanomyovirus S-PM2. Microbiology 154, 1775–1782. doi: 10.1099/mic.0.2007/016261-0 18524932

[ref10] Cui Y. R. Wang S. J. Chen J. Li J. Chen W. Wang S.. (2020). Allosteric inhibition of CRISPR-Cas9 by bacteriophage-derived peptides. Genome Biol. 21:51. doi: 10.1186/s13059-020-01956-x, PMID: 32102684 PMC7045643

[ref11] Dreher T. W. Brown N. Bozarth C. S. Schwartz A. D. Riscoe E. Thrash C.. (2011). A freshwater cyanophage whose genome indicates close relationships to photosynthetic marine cyanomyophages. Environ. Microbiol. 13, 1858–1874. doi: 10.1111/j.1462-2920.2011.02502.x, PMID: 21605306 PMC4185292

[ref12] Emms D. M. Kelly S. (2015). OrthoFinder: solving fundamental biases in whole genome comparisons dramatically improves orthogroup inference accuracy. Genome Biol. 16:157. doi: 10.1186/s13059-015-0721-2, PMID: 26243257 PMC4531804

[ref13] Enav H. Béjà O. Mandel-Gutfreund Y. (2012). Cyanophage tRNAs may have a role in cross-infectivity of oceanic *Prochlorococcus* and *Synechococcus* hosts. ISME J. 6, 619–628. doi: 10.1038/ismej.2011.146, PMID: 22011720 PMC3280135

[ref14] Falco S. C. Zehring W. Rothman-Denes L. B. (1980). DNA-dependent RNA polymerase from bacteriophage N4 virions. Purification and characterization. J. Biol. Chem. 255, 4339–4347. doi: 10.1016/S0021-9258(19)85670-3 6989837

[ref15] Gon S. Faulkner M. J. Beckwith J. (2006). In vivo requirement for glutaredoxins and thioredoxins in the reduction of the ribonucleotide reductases of *Escherichia coli*. Antioxid. Redox Signal. 8, 735–742. doi: 10.1089/ars.2006.8.735 16771665

[ref16] Grafstrom R. H. Park L. Grossman L. (1982). Enzymatic repair of pyrimidine dimer-containing DNA. A 50 dimer DNA glycosylase: 30-apyrimidinic endonuclease mechanism from *Micrococcus luteus*. J. Biol. Chem. 257, 13465–13474. doi: 10.1016/S0021-9258(18)33472-0, PMID: 7142160

[ref17] Gregory A. C. Zayed A. A. Conceição-Neto N. Temperton B. Bolduc B. Alberti A.. (2019). Marine DNA viral macro- and microdiversity from pole to pole. Cells 177, 1109–1123.e14. doi: 10.1016/j.cell.2019.03.040, PMID: 31031001 PMC6525058

[ref18] Guo X. Söderholm A. Kanchugal P. S. Isaksen G. V. Warsi O. Eckhard U.. (2021). Structure and mechanism of a phage-encoded SAM lyase revises catalytic function of enzyme family. eLife 10:e61818. doi: 10.7554/eLife.61818, PMID: 33567250 PMC7877911

[ref19] Huang L. Liu Q. Liu X. Wang Q. Zhao Q. Wang M.. (2020). Isolation and complete genome sequence of a novel cyanophage S-B68. Curr. Microbiol. 77, 2385–2390. doi: 10.1007/s00284-020-02045-9, PMID: 32451684

[ref20] Huang S. Zhang S. Jiao N. Chen F. (2015). Marine cyanophages demonstrate biogeographic patterns throughout the global ocean. Appl. Environ. Microbiol. 81, 441–452. doi: 10.1128/aem.02483-14, PMID: 25362060 PMC4272729

[ref21] Hui E. K. (2002). Virion-associated protein kinases. Cell. Mol. Life Sci. 59, 920–931. doi: 10.1007/s00018-002-8479-6 12169022 PMC11337488

[ref22] Ignacio-Espinoza J. C. Sullivan M. B. (2012). Phylogenomics of T4 cyanophages: lateral gene transfer in the 'core' and origins of host genes. Environ. Microbiol. 14, 2113–2126. doi: 10.1111/j.1462-2920.2012.02704.x 22348436

[ref23] Jerlström Hultqvist J. Warsi O. Söderholm A. Knopp M. Eckhard U. Vorontsov E.. (2018). A bacteriophage enzyme induces bacterial metabolic perturbation that confers a novel promiscuous function. Nat. Ecol. Evol. 2, 1321–1330. doi: 10.1038/s41559-018-0568-5, PMID: 29807996

[ref24] Jiang T. Guo C. Wang M. Wang M. Zhang X. Liu Y.. (2020). Genome analysis of two novel *Synechococcus* phages that lack common auxiliary metabolic genes: possible reasons and ecological insights by comparative analysis of cyanomyoviruses. Viruses 12:800. doi: 10.3390/v12080800, PMID: 32722486 PMC7472177

[ref25] Katoh K. Asimenos G. Toh H. (2009). Multiple alignment of DNA sequences with MAFFT. Methods Mol. Biol. 537, 39–64. doi: 10.1007/978-1-59745-251-9_3 19378139

[ref26] Kazmierczak K. M. Davydova E. K. Mustaev A. A. Rothman-Denes L. B. (2002). The phage N4 virion RNA polymerase catalytic domain is related to single-subunit RNA polymerases. EMBO J. 21, 5815–5823. doi: 10.1093/emboj/cdf584, PMID: 12411499 PMC131081

[ref27] Kemp M. G. Hu J. (2017). Postexcision events in human nucleotide excision repair. Photochem. Photobiol. 93, 178–191. doi: 10.1111/php.12641, PMID: 27645806 PMC5315629

[ref28] Koehn E. M. Kohen A. (2010). Flavin-dependent thymidylate synthase: a novel pathway towards thymine. Arch. Biochem. Biophys. 493, 96–102. doi: 10.1016/j.abb.2009.07.016, PMID: 19643076 PMC2812616

[ref29] Kumar S. Stecher G. Tamura K. (2016). MEGA7: molecular evolutionary genetics analysis version 7.0 for bigger datasets. Mol. Biol. Evol. 33, 1870–1874. doi: 10.1093/molbev/msw054, PMID: 27004904 PMC8210823

[ref30] Li D. Luo R. Liu C. M. Leung C. M. Ting H. F. Sadakane K.. (2016). MEGAHIT v1.0: a fast and scalable metagenome assembler driven by advanced methodologies and community practices. Methods 102, 3–11. doi: 10.1016/j.ymeth.2016.02.020, PMID: 27012178

[ref31] Loenen W. A. (2006). S-adenosylmethionine: jack of all trades and master of everything? Biochem. Soc. Trans. 34, 330–333. doi: 10.1042/BST20060330, PMID: 16545107

[ref32] Mann N. H. Clokie M. R. J. (2012). “Cyanophages, p 535–557” in Ecology of cyanobacteria II: Their diversity in space and time. ed. Whitton B. A. (Netherlands: Springer, Dordrecht), 535–557.

[ref33] Marchler-Bauer A. Lu S. Anderson J. B. Chitsaz F. Derbyshire M. K. DeWeese-Scott C.. (2011). CDD: a conserved domain database for the functional annotation of proteins. Nucleic Acids Res. 39, D225–D229. doi: 10.1093/nar/gkq1189, PMID: 21109532 PMC3013737

[ref34] Martinez-Hernandez F. Fornas O. Lluesma Gomez M. Bolduc B. de la Cruz Peña M. J. Martínez J. M.. (2017). Single-virus genomics reveals hidden cosmopolitan and abundant viruses. Nat. Commun. 8:15892. doi: 10.1038/ncomms15892 28643787 PMC5490008

[ref35] Michalski A. Damoc E. Hauschild J. P. Lange O. Wieghaus A. Makarov A.. (2011). Mass spectrometry-based proteomics using Q Exactive, a high-performance benchtop quadrupole Orbitrap mass spectrometer. Mol. Cell. Proteomics 10:M111.011015. doi: 10.1074/mcp.M111.011015, PMID: 21642640 PMC3284220

[ref36] Miller E. S. Kutter E. Mosig G. Arisaka F. Kunisawa T. Rüger W. (2003). Bacteriophage T4 genome. Microbiol. Mol. Biol. Rev. 67, 86–156. doi: 10.1128/MMBR.67.1.86-156.2003, PMID: 12626685 PMC150520

[ref37] Mizuno C. M. Ghai R. Saghaï A. López-García P. Rodriguez-Valera F. (2016). Genomes of abundant and widespread viruses from the deep ocean. MBio 7, e00805–e00816. doi: 10.1128/mBio.00805-16, PMID: 27460793 PMC4981710

[ref38] Moraru C. Varsani A. Kropinski A. M. (2020). VIRIDIC-A novel tool to calculate the intergenomic similarities of prokaryote-infecting viruses. Viruses 12:1268. doi: 10.3390/v12111268, PMID: 33172115 PMC7694805

[ref39] Myllykallio H. Lipowski G. Leduc D. Filee J. Forterre P. Liebl U. (2002). An alternative flavin-dependent mechanism for thymidylate synthesis. Science 297, 105–107. doi: 10.1126/science.1072113, PMID: 12029065

[ref40] Narasimhan V. Danecek P. Scally A. Xue Y. Tyler-Smith C. Durbin R. (2016). BCFtools/RoH: a hidden Markov model approach for detecting autozygosity from next-generation sequencing data. Bioinformatics 32, 1749–1751. doi: 10.1093/bioinformatics/btw044, PMID: 26826718 PMC4892413

[ref41] Noguchi H. Taniguchi T. Itoh T. (2008). MetaGeneAnnotator: detecting species-specific patterns of ribosomal binding site for precise gene prediction in anonymous prokaryotic and phage genomes. DNA Res. 15, 387–396. doi: 10.1093/dnares/dsn027, PMID: 18940874 PMC2608843

[ref42] Peng Y. Leung H. C. Yiu S. M. Chin F. Y. (2012). IDBA-UD: a de novo assembler for single-cell and metagenomic sequencing data with highly uneven depth. Bioinformatics 28, 1420–1428. doi: 10.1093/bioinformatics/bts174 22495754

[ref43] Pruitt K. D. Tatusova T. Maglott D. R. (2007). NCBI reference sequences (RefSeq): a curated non-redundant sequence database of genomes, transcripts and proteins. Nucleic Acids Res. 35, D61–D65. doi: 10.1093/nar/gkl842, PMID: 17130148 PMC1716718

[ref44] Puxty R. J. Millard A. D. Evans D. J. Scanlan D. J. (2016). Viruses inhibit CO_2_ fixation in the most abundant phototrophs on earth. Curr. Biol. 26, 1585–1589. doi: 10.1016/j.cub.2016.04.036, PMID: 27291056

[ref45] Raffaelli N. Emanuelli M. Pisani F. M. Amici A. Lorenzi T. Ruggueru S.. (1999). Identification of the archaeal NMN adenylytransferase gene. Mol. Cell. Biochem. 193, 99–102. doi: 10.1023/A:1006968328186 10331644

[ref46] Raffaelli N. Pisani F. M. Lorenzi T. Emanuelli M. Amici A. Ruggieri S.. (1997). Characterization of nicotinamide mononucleotide adenylyltransferase from thermophilic archaea. J. Bacteriol. 179, 7718–7723. doi: 10.1128/jb.179.24.7718-7723.1997, PMID: 9401030 PMC179734

[ref47] Raffaelli N. Pisani F. M. Lorenzi T. Emanuelli M. Amici A. Ruggieri S.. (2001). Nicotinamide-mononucleotide adenylyltransferase from *Methanococcus jannaschii*. Methods Enzymol. 331, 292–298. doi: 10.1016/s0076-6879(01)31066-2 11265471

[ref48] Rong C. Zhou K. Li S. Xiao K. Xu Y. Zhang R.. (2022). Isolation and characterization of a novel cyanophage encoding multiple auxiliary metabolic genes. Viruses 14:887. doi: 10.3390/v14050887 35632629 PMC9146016

[ref49] Sabehi G. Shaulov L. Silver D. H. Yanai I. Harel A. Lindell D. (2012). A novel lineage of myoviruses infecting cyanobacteria is widespread in the oceans. Proc. Natl. Acad. Sci. U. S. A. 109, 2037–2042. doi: 10.1073/pnas.1115467109, PMID: 22308387 PMC3277518

[ref50] Six C. Thomas J. C. Garczarek L. Ostrowski M. Dufresne A. Blot N.. (2007). Diversity and evolution of phycobilisomes in marine *Synechococcus* spp.: a comparative genomics study. Genome Biol. 8:R259. doi: 10.1186/gb-2007-8-12-r259, PMID: 18062815 PMC2246261

[ref51] Söding J. Biegert A. Lupas A. N. (2005). The HHpred interactive server for protein homology detection and structure prediction. Nucleic Acids Res. 33, W244–W248. doi: 10.1093/nar/gki408, PMID: 15980461 PMC1160169

[ref52] Sohm J. A. Ahlgren N. A. Thomson Z. J. Williams C. Moffett J. W. Saito M. A.. (2016). Co-occurring *Synechococcus* ecotypes occupy four major oceanic regimes defined by temperature, macronutrients and iron. ISME J. 10, 333–345. doi: 10.1038/ismej.2015.115, PMID: 26208139 PMC4737926

[ref54] Stamatakis A. (2014). RAxML version 8: a tool for phylogenetic analysis and post-analysis of large phylogenies. Bioinformatics 30, 1312–1313. doi: 10.1093/bioinformatics/btu033, PMID: 24451623 PMC3998144

[ref55] Sullivan M. B. Coleman M. L. Quinlivan V. Rosenkrantz J. E. DeFrancesco A. S. Tan G.. (2008). Portal protein diversity and phage ecology. Environ. Microbiol. 10, 2810–2823. doi: 10.1111/j.1462-2920.2008.01702.x, PMID: 18673386 PMC2657995

[ref56] Sullivan M. B. Coleman M. L. Weigele P. Rohwer F. Chisholm S. W. (2005). Three *Prochlorococcus* cyanophage genomes: signature features and ecological interpretations. PLoS Biol. 3:e144. doi: 10.1371/journal.pbio.0030144, PMID: 15828858 PMC1079782

[ref57] Sullivan M. B. Huang K. H. Ignacio-Espinoza J. C. Berlin A. M. Kelly L. Weigele P. R.. (2010). Genomic analysis of oceanic cyanobacterial myoviruses compared with T4-like myoviruses from diverse hosts and environments. Environ. Microbiol. 12, 3035–3056. doi: 10.1111/j.1462-2920.2010.02280.x, PMID: 20662890 PMC3037559

[ref58] Sullivan M. B. Krastins B. Hughes J. L. Kelly L. Chase M. Sarracino D.. (2009). The genome and structural proteome of an ocean siphovirus: a new window into the cyanobacterial 'mobilome'. Environ. Microbiol. 11, 2935–2951. doi: 10.1111/j.1462-2920.2009.02081.x, PMID: 19840100 PMC2784084

[ref59] Sullivan M. J. Petty N. K. Beatson S. A. (2011). Easyfig: a genome comparison visualizer. Bioinformatics 27, 1009–1010. doi: 10.1093/bioinformatics/btr039, PMID: 21278367 PMC3065679

[ref60] Sullivan M. B. Waterbury J. B. Chisholm S. W. (2003). Cyanophages infecting the oceanic cyanobacterium *Prochlorococcus*. Nature 424, 1047–1051. doi: 10.1038/nature01929, PMID: 12944965

[ref61] Sun M. Zhan Y. Marsan D. Páez-Espino D. Cai L. Chen F. (2021). Uncultivated viral populations dominate estuarine viromes on the spatiotemporal scale. mSystems. 6:e01020. doi: 10.1128/mSystems.01020-20, PMID: 33727395 PMC8546989

[ref62] Suttle C. A. Chen F. (1992). Mechanisms and rates of decay of marine viruses in seawater. Appl. Environ. Microbiol. 58, 3721–3729. doi: 10.1128/aem.58.11.3721-3729.1992, PMID: 16348812 PMC183166

[ref63] Thompson L. R. Zeng Q. Kelly L. Huang K. H. Singer A. U. Stubbe J. A.. (2011). Phage auxiliary metabolic genes and the redirection of cyanobacterial host carbon metabolism. Proc. Natl. Acad. Sci. U. S. A. 108, E757–E764. doi: 10.1073/pnas.1102164108 21844365 PMC3182688

[ref64] Thomsen L. E. Chadfield M. S. Bispham J. Wallis T. S. Olsen J. E. Ingmer H. (2003). Reduced amounts of LPS affect both stress tolerance and virulence of *Salmonella enterica* serovar Dublin. FEMS Microbiol. Lett. 228, 225–231. doi: 10.1016/S0378-1097(03)00762-6, PMID: 14638428

[ref65] Walker R. K. Mccullough A. K. Lloyd R. S. (2006). Uncoupling of nucleotide flipping and DNA bending by the T4 pyrimidine dimer DNA glycosylase. Biochemistry 45, 14192–14200. doi: 10.1021/bi060802s, PMID: 17115714 PMC2673921

[ref66] Wang Q. Cai L. Zhang R. Wei S. Li F. Liu Y.. (2022). A unique set of auxiliary metabolic genes found in an isolated cyanophage sheds new light on marine phage-host interactions. Microbiol. Spectr. 10:e0236722. doi: 10.1128/spectrum.02367-22, PMID: 36190421 PMC9602691

[ref67] Wang Y. Ferrinho S. Connaris H. Goss R. J. M. (2023). The impact of viral infection on the chemistries of the earth’s most abundant photosynthesizes: metabolically talented aquatic cyanobacteria. Biomol. Ther. 13:1218. doi: 10.3390/biom13081218, PMID: 37627283 PMC10452541

[ref68] Waterbury J. B. Willey J. M. (1988). “Isolation and growth of marine planktonic cyanobacteria” in Methods in enzymology—cyanobacteria. eds. Packer L. Glazer A. N., vol. 167 (San Diego, CA: Academic Press), 100–105.

[ref69] Weinberg Z. Wang J. X. Bogue J. Yang J. Corbino K. Moy R. H.. (2010). Comparative genomics reveals 104 candidate structured RNAs from bacteria, archaea, and their metagenomes. Genome Biol. 11:R31. doi: 10.1186/gb-2010-11-3-r31, PMID: 20230605 PMC2864571

[ref70] Wilson G. G. Murray N. E. (1991). Restriction and modification systems. Annu. Rev. Genet. 25, 585–627. doi: 10.1146/annurev.ge.25.120191.003101 1812816

[ref71] Wiśniewski J. R. Zougman A. Nagaraj N. Mann M. (2009). Universal sample preparation method for proteome analysis. Nat. Methods 6, 359–362. doi: 10.1038/nmeth.1322 19377485

[ref72] Wright G. D. Thompson P. R. (1999). Aminoglycoside phosphotransferases: proteins, structure, and mechanism. Front. Biosci. 4, D9–D21. doi: 10.2741/wright 9872733

[ref73] Xia X. Vidyarathna N. K. Palenik B. Lee P. Liu H. (2015). Comparison of the seasonal variations of *Synechococcus* assemblage structures in estuarine waters and coastal waters of Hong Kong. Appl. Environ. Microbiol. 81, 7644–7655. doi: 10.1128/AEM.01895-15, PMID: 26319880 PMC4592875

[ref74] Xu B. Li F. Cai L. Zhang R. Fan L. Zhang C. (2022). A holistic genome dataset of bacteria, archaea and viruses of the Pearl River estuary. Sci Data. 9:49. doi: 10.1038/s41597-022-01153-4, PMID: 35165305 PMC8844013

[ref75] Xu Y. Zhang R. Jiao N. (2015). Complete genome sequence of *Paracoccus marcusii* phage vB_PmaS-R3 isolated from the South China Sea. Stand. Genomic Sci. 10:94. doi: 10.1186/s40793-015-0089-7 26561517 PMC4641407

[ref76] Xu Y. Zhang R. Wang N. Cai L. Tong Y. Sun Q.. (2018). Novel phage-host interactions and evolution as revealed by a cyanomyovirus isolated from an estuarine environment. Environ. Microbiol. 20, 2974–2989. doi: 10.1111/1462-2920.14326, PMID: 30051557

[ref77] Yao Z. Barrick J. Weinberg Z. Neph S. Breaker R. Tompa M.. (2007). A computational pipeline for high- throughput discovery of *cis*-regulatory noncoding RNA in prokaryotes. PLoS Comput. Biol. 3:e126. doi: 10.1371/journal.pcbi.0030126, PMID: 17616982 PMC1913097

[ref78] Zheng H. Liu Y. Zhou R. Liu J. Xu Y. Chen F. (2023). An estuarine cyanophage S-CREM1 encodes three distinct antitoxin genes and a large number of non-coding RNA genes. Viruses 15:380. doi: 10.3390/v15020380, PMID: 36851594 PMC9964418

