# Supplementary material for: A novel long-tailed myovirus represents a new T4-like cyanophage cluster
Source: Front Microbiol. 2023 Nov 9;14:1293846. doi: 10.3389/fmicb.2023.1293846 (PMC10665884; doi:10.3389/fmicb.2023.1293846)
Supplement: Supplementary file 1 [file Data_Sheet_1.docx]

Supplementary Material


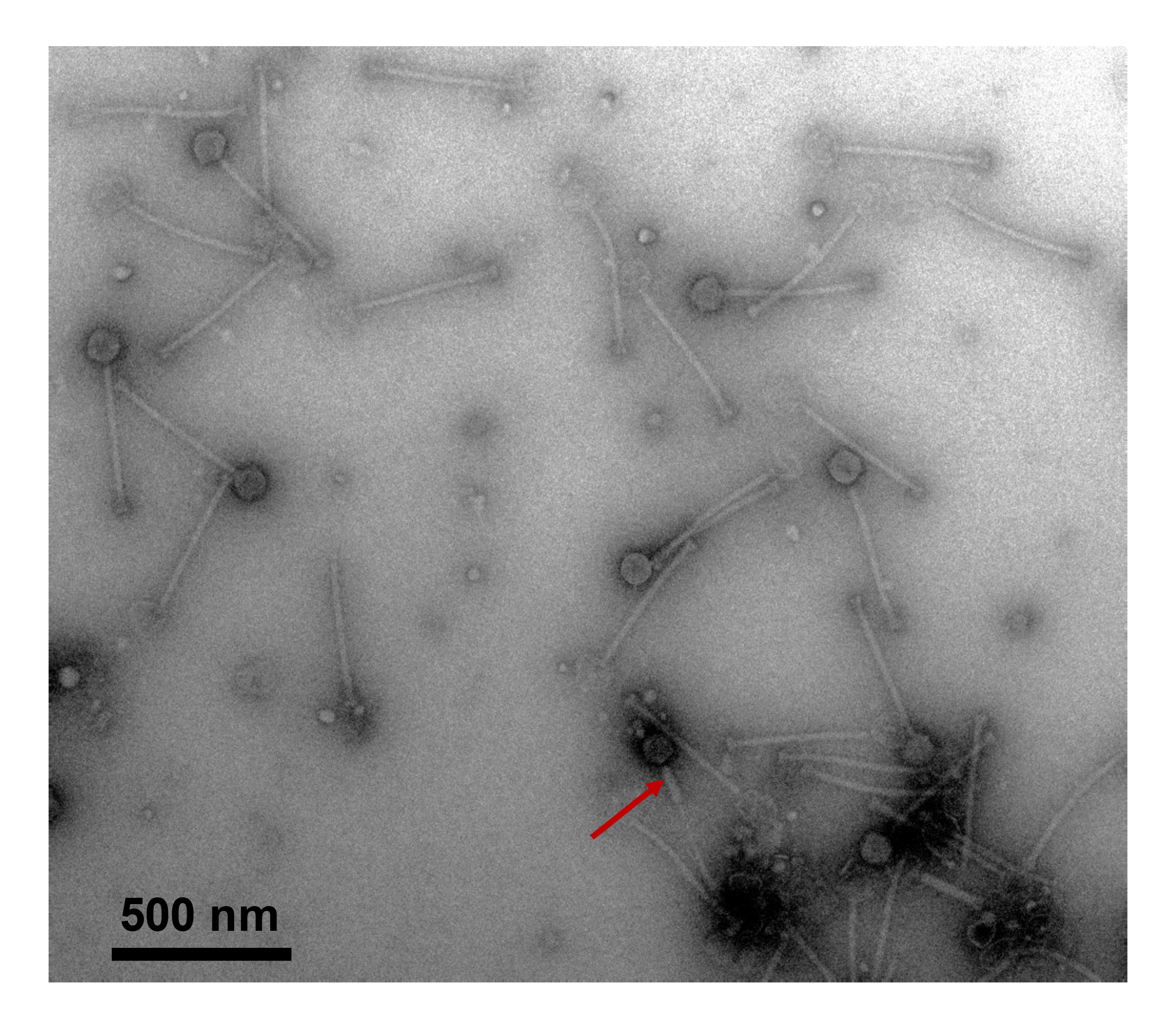
Supplementary Figure S1. Transmission electron microscopy image of *Synechococcus* phage S-CREM2. The red arrow indicates the contractile tail sheath.


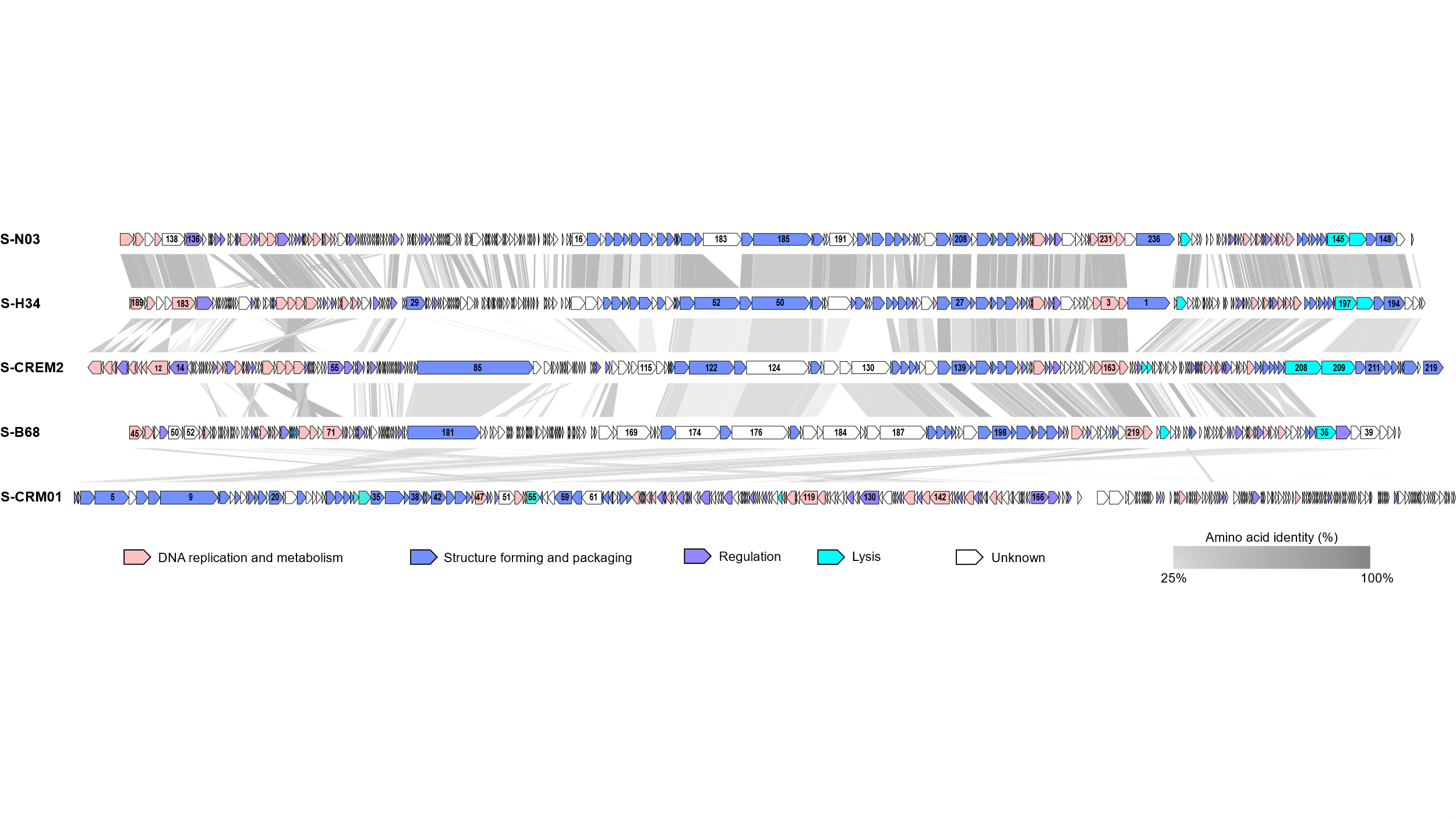
Supplementary Figure S2. Comparative genomic analyses of the T4-like cluster C cyanophages. Predicted ORFs are represented by arrows, with the left or right arrow indicating the transcription direction. The number inside each arrow indicates the ORF number. ORFs with different functions are indicated by different colored arrows. The shading color that connects homologous genes between cyanophages indicates the degree of shared amino acid identity between genes. The genomes of S-N03, S-H34, and S-B68 were rearranged for easy comparison.


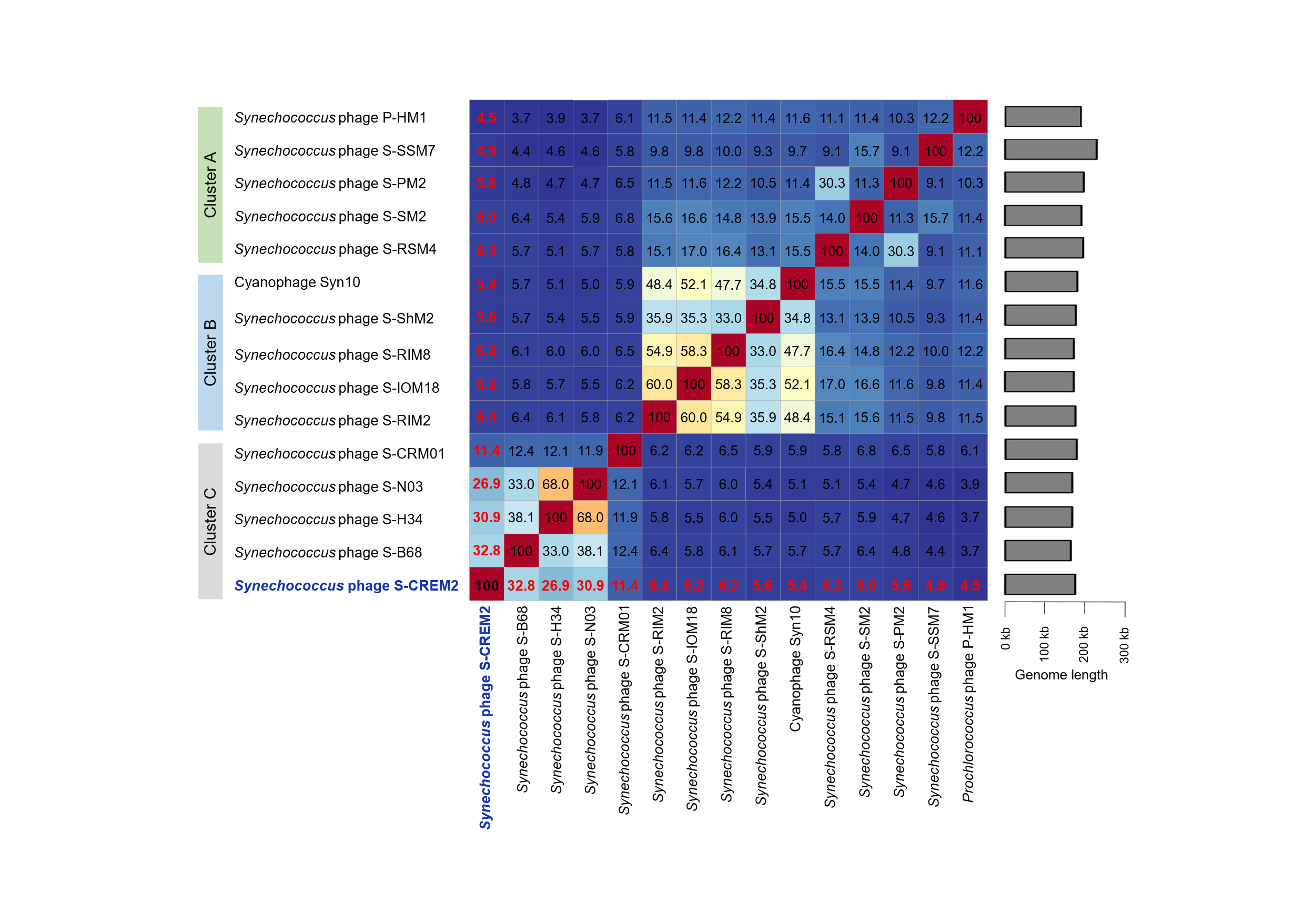
Supplementary Figure S3. Intergenomic similarities among S-CREM2 and 14 T4-like cyanophages calculated by VIRIDIC. Genomic similarities between S-CREM2 and the 14 cyanophages are displayed in red and bold fonts.


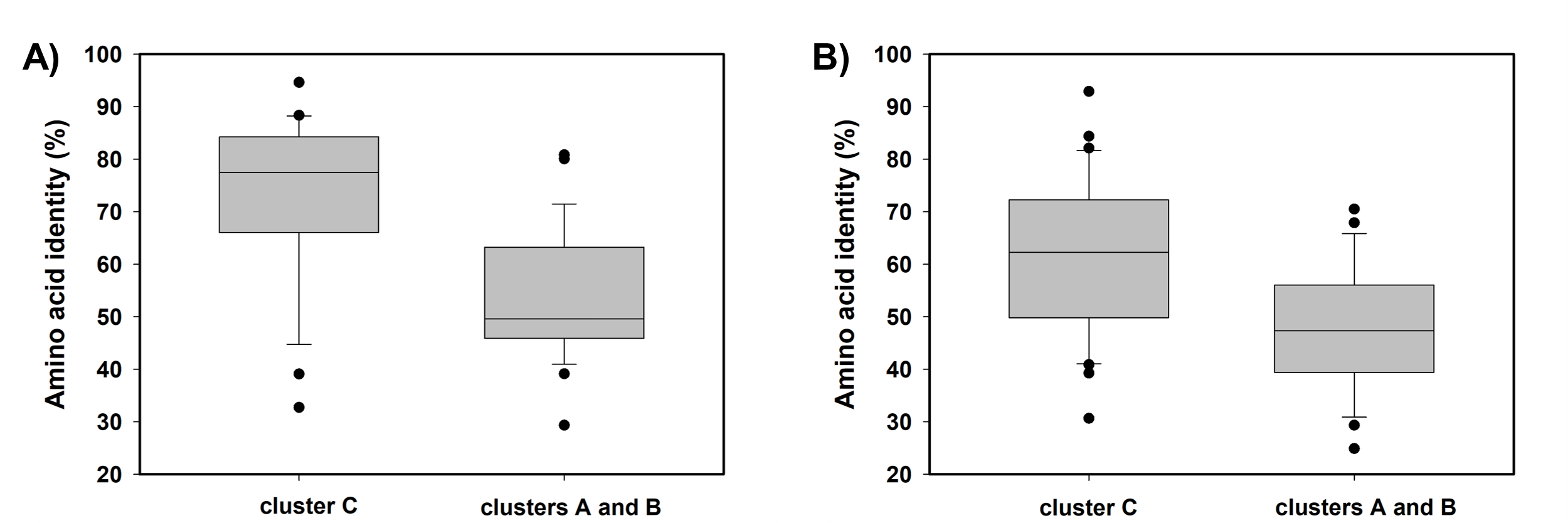


Supplementary Figure S4. Highest amino acids identities between S-CREM2 and T4-like cluster C cyanophages and between S-CREM2 and T4-like clusters A and B cyanophages for each DNA replication and metabolism gene (A) and each structural gene (B).

| Supplementary Table S1. Information of metagenome datasets. | | | |
| --- | --- | --- | --- |
| Metagenome dataset | Source | Project code/Accession no. | Number of datasets used |
| Delmarva Estuarine Virome (DEV) | NCBI Short Read Archive (SRA) | PRJNA365444 | 42 |
| Pearl River Estuary (PRE) | NCBI Short Read Archive (SRA) | SRR15927516-SRR15927530 | 15 |
| Global Oceans Viromes (GOV 2.0) dataset | European Nucleotide Archive | ERR594379-ERR594383, ERR594399-ERR594401, ERR594410-ERR594412, ERR594357, ERR594359, ERR594361, ERR594364, ERR594374, ERR594375, ERR594386, ERR594387, ERR594396, ERR594414, ERR599337, ERR599339, ERR599340, ERR599342, ERR599344, ERR599345, ERR599350, ERR599355, ERR599357, ERR599359, ERR599363, ERR599369, ERR599374, ERR599375, ERR2762104-ERR2762107, ERR2762116-ERR2762120, ERR2762124-ERR2762125, ERR2762146-ERR2762166, ERR2762180-ERR2762182, ERR2762134-ERR2762137, ERR2752143, ERR2752144, ERR2752156, ERR2762100, ERR2762101, ERR2762110, ERR2762111, ERR2762142, ERR2762143, ERR2752158 | 63 |

| Supplementary Table S2. Predicted ORFs in the S-CREM2 genome that have homologs in the non-redundant database. | | | | | | | | | |
| --- | --- | --- | --- | --- | --- | --- | --- | --- | --- |
| Gene | Strand | Left | Right | aa length | Significant hits in NR database | ^a^Putative function | aa identity | E-value | ^b^Conserved domain (e-value) |
| 1 | - | 1740 | 19 | 574 | recombination-related endonuclease [*Synechococcus* phage S-B68] | CDNA repair exonuclease SbcCD ATPase subunit | 69.2% | 0 | COG0419 (1.6E-21) |
|  |  |  |  |  | recombination-related endonuclease [*Synechococcus* phage S-N03] |  | 65.7% | 0 |  |
|  |  |  |  |  | recombination-related endonuclease [*Synechococcus* phage S-H34] |  | 65.6% | 0 |  |
|  |  |  |  |  | SbcC-like subunit of palindrome specific endonuclease [*Synechococcus* phage S-CRM01] |  | 50.6% | 0 |  |
|  |  |  |  |  | exonuclease [*Synechococcus* phage S-SCSM1] |  | 41.5% | 1.6E-148 |  |
| 2 | - | 2015 | 1737 | 93 | hypothetical protein [*Synechococcus* phage S-B68] |  | 55.9% | 1.9E-23 |  |
|  |  |  |  |  | hypothetical cyanophage protein [*Synechococcus* phage S-CRM01] |  | 44.1% | 2.3E-22 |  |
|  |  |  |  |  | hypothetical protein PQC09_gp144 [*Synechococcus* phage S-N03] |  | 55% | 5.8E-20 |  |
|  |  |  |  |  | hypothetical protein PQC15_gp188 [*Synechococcus* phage S-H34] |  | 52.5% | 1.7E-17 |  |
| 3 | - | 3123 | 2077 | 349 | recombination-related endonuclease [*Synechococcus* phage S-B68] | exonuclease subunit I | 79.8% | 0 | PHA02546 (1.0E-121) |
|  |  |  |  |  | SbcD-like subunit of palindrome specific endonuclease [*Synechococcus* phage S-H34] |  | 75.1% | 0 |  |
|  |  |  |  |  | SbcD-like subunit of palindrome specific endonuclease [*Synechococcus* phage S-N03] |  | 73.9% | 0 |  |
|  |  |  |  |  | SbcD-like subunit of palindrome specific endonuclease [*Synechococcus* phage S-CRM01] |  | 63.3% | 3.4E-166 |  |
|  |  |  |  |  | SbcD-like subunit of palindrome specific endonuclease [*Synechococcus* phage S-SRM01] |  | 49.6% | 2.3E-116 |  |
| 4 | - | 3589 | 3104 | 162 | hypothetical protein GCM10018777_31540 [*Streptomyces* viridodiastaticus] | recombination endonuclease VII | 57.1% | 5.8E-25 | pfam02945 (4.4E-28) |
|  |  |  |  |  | endonuclease VII [*Synechococcus* phage S-CBS2] |  | 43% | 1.1E-18 |  |
| 6 | - | 5081 | 3837 | 415 | hypothetical protein PQC15_gp185 [*Synechococcus* phage S-H34] |  | 73.3% | 1.1E-131 |  |
|  |  |  |  |  | hypothetical protein PQC09_gp141 [*Synechococcus* phage S-N03] |  | 56.3% | 6.2E-93 |  |
|  |  |  |  |  | hypothetical protein AVU42_gp123 [*Prochlorococcus* phage P-TIM68] |  | 51.5% | 1.4E-22 |  |
| 7 | - | 5248 | 5102 | 49 | hypothetical protein PQC15_gp184 [*Synechococcus* phage S-H34] |  | 58.5% | 8.1E-09 |  |
|  |  |  |  |  | hypothetical protein PQC09_gp140 [*Synechococcus* phage S-N03] |  | 51.2% | 6.7E-07 |  |
| 8 | - | 6135 | 5245 | 297 | DNA adenine methylase [*Synechococcus* phage S-H34] | DNA adenine methylase | 84.8% | 0 | TIGR00571 (9.9E-26) |
|  |  |  |  |  | DNA adenine methylase [*Synechococcus* phage S-N03] |  | 84.8% | 0 |  |
|  |  |  |  |  | DNA adenine methylase [*Synechococcus* phage ACG-2014f] |  | 72.3% | 1.9E-158 |  |
| 10 | - | 6983 | 6477 | 169 | hypothetical protein PQC15_gp102 [*Synechococcus* phage S-H34] | pyrimidine dimer DNA glycosylase | 82.4% | 6.2E-99 | pfam03013 (7.9E-10) |
|  |  |  |  |  | hypothetical protein PQC09_gp055 [*Synechococcus* phage S-N03] |  | 79% | 6.4E-97 |  |
|  |  |  |  |  | hypothetical protein [*Synechococcus* phage S-B68] |  | 77.6% | 5.4E-90 |  |
|  |  |  |  |  | DNA binding protein [*Prochlorococcus* phage P-SSM7] |  | 47.7% | 3.3E-28 |  |
| 11 | - | 7623 | 7054 | 190 | hypothetical protein NJ_05_1013_058 [*Synechococcus* phage S-RIM2] |  | 67.9% | 1.1E-84 |  |
|  |  |  |  |  | hypothetical protein SBM1_00185 [*Synechococcus* phage S-BM1] |  | 67.9% | 1.5E-84 |  |
| 12 | - | 10313 | 7704 | 870 | hypothetical protein PQC15_gp182 [*Synechococcus* phage S-H34] |  | 63.5% | 9.4E-36 |  |
|  |  |  |  |  | hypothetical protein PQC09_gp138 [*Synechococcus* phage S-N03] |  | 50.5% | 2.0E-32 |  |
|  |  |  |  |  | hypothetical protein Syn7803C17_183 [*Synechococcus* phage ACG-2014f] |  | 28% | 4.3E-53 |  |
|  |  |  |  |  | hypothetical protein [*Synechococcus* phage S-B68] |  | 61.9% | 3.3E-47 |  |
|  |  |  |  |  | hypothetical protein SCRM01_128c [*Synechococcus* phage S-CRM01] |  | 45.6% | 4.5E-31 |  |
| 13 | - | 10546 | 10310 | 79 | hypothetical protein [*Synechococcus* phage S-B68] |  | 57.7% | 1.9E-18 |  |
|  |  |  |  |  | hypothetical protein PQC15_gp181 [*Synechococcus* phage S-H34] |  | 49.4% | 1.3E-13 |  |
|  |  |  |  |  | hypothetical protein PQC09_gp137 [*Synechococcus* phage S-N03] |  | 49.4% | 3.6E-11 |  |
| 14 | - | 12846 | 10579 | 756 | serine/threonine kinase [*Synechococcus* phage S-H34] | APH/ChoK-like kinase | 36.8% | 1.4E-127 | cd05120 (4.6E-05) |
|  |  |  |  |  | hypothetical protein [*Synechococcus* phage S-B68] |  | 36.4% | 1.6E-115 |  |
|  |  |  |  |  | serine/threonine kinase [*Synechococcus* phage S-N03] |  | 42.2% | 6.4E-98 |  |
|  |  |  |  |  | serine/threonine kinase [*Synechococcus* phage S-N03] |  | 42.7% | 5.5E-30 |  |
|  |  |  |  |  | serine/threonine kinase PKN8 [*Synechococcus* phage S-CRM01] |  | 32% | 1.2E-93 |  |
| 15 | - | 13331 | 12843 | 163 | hypothetical protein [*Synechococcus* phage S-B68] |  | 43% | 2.1E-29 |  |
|  |  |  |  |  | hypothetical protein PQC09_gp135 [*Synechococcus* phage S-N03] |  | 37.6% | 1.1E-24 |  |
|  |  |  |  |  | hypothetical protein PQC15_gp179 [*Synechococcus* phage S-H34] |  | 38.6% | 5.6E-24 |  |
|  |  |  |  |  | hypothetical protein SCRM01_131c [*Synechococcus* phage S-CRM01] |  | 36.9% | 6.8E-18 |  |
| 17 | + | 13692 | 13934 | 81 | hypothetical protein PQC09_gp095 [*Synechococcus* phage S-N03] |  | 69.6% | 6.8E-33 |  |
|  |  |  |  |  | hypothetical protein PQC15_gp141 [*Synechococcus* phage S-H34] |  | 60.8% | 1.5E-27 |  |
|  |  |  |  |  | hypothetical protein BOW87_gp047 [*Synechococcus* phage S-CAM3] |  | 51.3% | 2.3E-16 |  |
|  |  |  |  |  | hypothetical protein [*Synechococcus* phage S-B68] |  | 49.3% | 7.2E-11 |  |
| 18 | + | 13931 | 14413 | 161 | hypothetical protein [*Synechococcus* sp. UW140] |  | 38% | 3.8E-17 |  |
| 19 | + | 14416 | 14646 | 77 | hypothetical protein PQC15_gp139 [*Synechococcus* phage S-H34] |  | 69.1% | 2.5E-26 |  |
|  |  |  |  |  | hypothetical protein PQC09_gp093 [*Synechococcus* phage S-N03] |  | 54.4% | 7.7E-13 |  |
|  |  |  |  |  | hypothetical protein [*Synechococcus* phage S-B68] |  | 55.7% | 1.8E-12 |  |
|  |  |  |  |  | hypothetical protein SWZG_00226 [*Synechococcus* phage S-SKS1] |  | 46.5% | 1.9E-09 |  |
| 20 | + | 14665 | 14919 | 85 | hypothetical protein PQC15_gp136 [*Synechococcus* phage S-H34] |  | 54.8% | 2.1E-25 |  |
|  |  |  |  |  | hypothetical protein [*Synechococcus* phage S-B68] |  | 35.7% | 5.8E-06 |  |
|  |  |  |  |  | hypothetical protein PQC09_gp090 [*Synechococcus* phage S-N03] |  | 28.8% | 6.3E-06 |  |
| 22 | + | 15183 | 15350 | 56 | hypothetical protein PQC15_gp135 [*Synechococcus* phage S-H34] |  | 84.9% | 3.6E-27 |  |
|  |  |  |  |  | hypothetical protein PQC09_gp088 [*Synechococcus* phage S-N03] |  | 86.5% | 1.0E-26 |  |
|  |  |  |  |  | hypothetical protein [*Synechococcus* phage S-B68] |  | 70.8% | 2.4E-13 |  |
|  |  |  |  |  | GDSL family lipase [*Synechococcus* phage S-CRM01] |  | 58.5% | 8.3E-13 |  |
| 23 | + | 15393 | 15755 | 121 | hypothetical protein PQC09_gp087 [*Synechococcus* phage S-N03] |  | 37.4% | 5.6E-08 |  |
|  |  |  |  |  | hypothetical protein PQC15_gp134 [*Synechococcus* phage S-H34] |  | 35.3% | 2.3E-07 |  |
| 25 | + | 16152 | 16655 | 168 | nucleotide kinase [*Synechococcus* phage S-H34] |  | 42.1% | 1.3E-33 |  |
|  |  |  |  |  | nucleotide kinase [*Synechococcus* phage S-N03] |  | 42.4% | 3.7E-31 |  |
| 26 | + | 16703 | 17368 | 222 | sliding clamp DNA polymerase accessory protein [*Synechococcus* phage S-H34] | sliding clamp | 76.5% | 1.9E-119 | PHA02545 (1.3E-67) |
|  |  |  |  |  | sliding clamp DNA polymerase accessory protein [*Synechococcus* phage S-N03] |  | 76% | 1.4E-117 |  |
|  |  |  |  |  | sliding clamp DNA polymerase accessory protein [*Synechococcus* phage S-B68] |  | 75.6% | 1.0E-116 |  |
|  |  |  |  |  | DNA polymerase processivity factor [*Synechococcus* phage S-CRM01] |  | 61.6% | 8.0E-88 |  |
|  |  |  |  |  | putative sliding clamp DNA polymerase accessory protein [*Synechococcus* phage S-B05] |  | 56.8% | 3.5E-79 |  |
| 27 | + | 17409 | 17591 | 61 | hypothetical protein [*Synechococcus* phage S-B68] |  | 77.6% | 1.4E-23 |  |
|  |  |  |  |  | hypothetical protein PQC15_gp125 [*Synechococcus* phage S-H34] |  | 49.1% | 6.0E-11 |  |
|  |  |  |  |  | hypothetical protein PQC09_gp077 [*Synechococcus* phage S-N03] |  | 46% | 2.0E-09 |  |
|  |  |  |  |  | hypothetical protein C440309_056 [*Synechococcus* phage S-CAM4] |  | 38.2% | 7.0E-06 |  |
| 28 | + | 17588 | 17719 | 44 | hypothetical protein PQC09_gp107 [*Synechococcus* phage S-N03] |  | 62.5% | 1.5E-06 |  |
| 29 | + | 17725 | 17946 | 74 | hypothetical protein [*Synechococcus* phage S-B68] |  | 39.7% | 3.3E-08 |  |
| 30 | + | 17943 | 18947 | 335 | pectate lyase [*Gammaproteobacteria* bacterium] | pectate lyase | 38.3% | 2.9E-54 | TIGR02474 (1.9E-57) |
| 31 | + | 19015 | 19941 | 309 | replication factor C small subunit / DNA polymerase clamp loader subunit [*Synechococcus* phage S-N03] | clamp loader subunit | 67.2% | 3.6E-154 | PHA02544 (2.8E-100) |
|  |  |  |  |  | DNA polymerase clamp loader subunit [*Synechococcus* phage S-H34] |  | 66.9% | 3.5E-153 |  |
|  |  |  |  |  | replication factor C small subunit / DNA polymerase clamp loader subunit [*Synechococcus* phage S-B68] |  | 63.6% | 6.2E-150 |  |
|  |  |  |  |  | clamp loader of DNA polymerase [*Synechococcus* phage S-CRM01] |  | 46.3% | 1.9E-90 |  |
|  |  |  |  |  | sliding clamp loader [*Synechococcus* phage S-SZBM1] |  | 42.2% | 3.0E-84 |  |
| 33 | + | 20111 | 20485 | 125 | clamp loader subunit [*Synechococcus* phage S-N03] | clamp loader A subunit | 63.1% | 3.8E-48 | PHA02593 (1.6E-11) |
|  |  |  |  |  | DNA polymerase clamp loader subunit [*Synechococcus* phage S-H34] |  | 64.8% | 4.1E-48 |  |
|  |  |  |  |  | hypothetical protein [*Synechococcus* phage S-B68] |  | 63.3% | 5.1E-46 |  |
|  |  |  |  |  | clamp loader of DNA polymerase [*Synechococcus* phage S-CRM01] |  | 42.9% | 5.7E-25 |  |
|  |  |  |  |  | clamp loader of DNA polymerase [*Synechococcus* phage DSL-LC03] |  | 40.8% | 3.2E-23 |  |
|  |  |  |  |  | clamp loader of DNA polymerase [*Synechococcus* phage S-RIM2 R1_1999] |  | 43% | 3.8E-23 |  |
| 34 | + | 20482 | 20631 | 50 | hypothetical protein PQC10_gp109 [*Synechococcus* phage S-H9-2] |  | 59.1% | 9.5E-11 |  |
|  |  |  |  |  | hypothetical protein [*Synechococcus* phage S-B68] |  | 49% | 8.8E-07 |  |
| 35 | + | 20678 | 21073 | 132 | endoribonuclease translational repressor of early genes [*Synechococcus* phage S-B68] | translational repressor of early genes, regA | 75.2% | 1.6E-66 | pfam01818 (1.7E-46) |
|  |  |  |  |  | translation repressor [*Synechococcus* phage S-N03] |  | 67.9% | 2.6E-62 |  |
|  |  |  |  |  | translation repressor [*Synechococcus* phage S-CRM01] |  | 68.7% | 1.3E-61 |  |
|  |  |  |  |  | translation repressor [*Synechococcus* phage S-H34] |  | 65.9% | 1.1E-60 |  |
| 36 | + | 21185 | 21589 | 135 | Hsp20 heat shock protein [*Synechococcus* phage S-N03] | heat shock protein (*hsp20*) | 67.9% | 2.9E-62 | PRK10743 (4.2E-25) |
|  |  |  |  |  | Hsp20 heat shock protein [*Synechococcus* phage S-H34] |  | 66.4% | 3.8E-61 |  |
|  |  |  |  |  | hypothetical protein [*Synechococcus* phage S-B68] |  | 63.4% | 3.0E-54 |  |
|  |  |  |  |  | Hsp20 heat shock protein [*Synechococcus* phage S-CRM01] |  | 46.3% | 4.8E-35 |  |
| 37 | + | 21651 | 22055 | 135 | hypothetical protein [*Synechococcus* phage S-B68] |  | 44.8% | 1.7E-19 |  |
|  |  |  |  |  | hypothetical protein PQC09_gp116 [*Synechococcus* phage S-N03] |  | 38.5% | 5.5E-15 |  |
|  |  |  |  |  | hypothetical protein PQC15_gp162 [*Synechococcus* phage S-H34] |  | 38.5% | 6.2E-15 |  |
| 38 | + | 22055 | 22417 | 121 | hypothetical protein PQC15_gp163 [*Synechococcus* phage S-H34] |  | 54% | 2.0E-37 |  |
|  |  |  |  |  | hypothetical protein PQC09_gp117 [*Synechococcus* phage S-N03] |  | 52% | 7.3E-37 |  |
|  |  |  |  |  | hypothetical cyanophage protein [*Synechococcus* phage S-CRM01] |  | 34.6% | 3.5E-12 |  |
|  |  |  |  |  | hypothetical protein PHM1_152 [*Prochlorococcus* phage P-HM1] |  | 40.8% | 1.2E-10 |  |
| 40 | + | 22592 | 24055 | 488 | adhesin [*Synechococcus* phage S-N03] | DNA polymerase subunit A | 82.7% | 0 | PHA02524 (1.4E-77) |
|  |  |  |  |  | DNA polymerase [*Synechococcus* phage S-H34] |  | 82.1% | 0 |  |
|  |  |  |  |  | DNA polymerase [*Synechococcus* phage S-B68] |  | 82% | 0 |  |
|  |  |  |  |  | DNA polymerase [*Synechococcus* phage S-CRM01] |  | 56.5% | 0 |  |
|  |  |  |  |  | DNA polymerase [*Synechococcus* phage DSL-LC02] |  | 55.2% | 0 |  |
| 42 | + | 24435 | 25535 | 367 | DNA polymerase [*Synechococcus* phage S-H34] | DNA polymerase subunit B | 82.8% | 0 | PHA02523 (1.5E-74) |
|  |  |  |  |  | DNA polymerase [*Synechococcus* phage S-N03] |  | 80.6% | 0 |  |
|  |  |  |  |  | DNA polymerase [*Synechococcus* phage S-B68] |  | 79.9% | 0 |  |
|  |  |  |  |  | DNA polymerase [*Synechococcus* phage S-CRM01] |  | 63.3% | 9.5E-159 |  |
|  |  |  |  |  | DNA polymerase [*Synechococcus* phage DSL-LC02] |  | 59.5% | 3.3E-151 |  |
| 43 | + | 25541 | 26548 | 336 | recombinase [*Synechococcus* phage S-N03] | recA/radA recombinase | 88.4% | 0 | COG0468 (7.6E-28) |
|  |  |  |  |  | recombinase [*Synechococcus* phage S-H34] |  | 87.8% | 0 |  |
|  |  |  |  |  | RecA-like protein [*Synechococcus* phage S-B68] |  | 84.8% | 0 |  |
|  |  |  |  |  | DNA repair protein [*Synechococcus* phage S-CAM7] |  | 78.4% | 0 |  |
|  |  |  |  |  | UvsX [*Synechococcus* phage S-CRM01] |  | 71% | 3.5E-172 |  |
| 44 | + | 26613 | 28007 | 465 | DnaB-like replicative helicase [*Synechococcus* phage S-H34] | DNA primase | 78.2% | 0 | PHA02542 (0) |
|  |  |  |  |  | DnaB-like replicative helicase [*Synechococcus* phage S-N03] |  | 78% | 0 |  |
|  |  |  |  |  | DNA primase/helicase [*Synechococcus* phage S-B68] |  | 76.7% | 0 |  |
|  |  |  |  |  | DNA primase-helicase [*Synechococcus* phage S-SSM7] |  | 63.4% | 0 |  |
|  |  |  |  |  | DNA primase / helicase [*Synechococcus* phage S-CRM01] |  | 60.9% | 0 |  |
| 45 | + | 28010 | 28420 | 137 | MazG-like pyrophosphatase [*Synechococcus* phage S-N03] | *mazG* | 90.4% | 1.3E-84 | cd11541 (7.6E-21) |
|  |  |  |  |  | MazG-like pyrophosphatase [*Synechococcus* phage S-H34] |  | 88.2% | 1.2E-83 |  |
|  |  |  |  |  | pyrophosphatase [*Synechococcus* phage S-B68] |  | 87.5% | 1.6E-82 |  |
|  |  |  |  |  | MazG-like pyrophosphatase [*Synechococcus* phage S-CRM01] |  | 72.1% | 1.7E-67 |  |
|  |  |  |  |  | MazG-like pyrophosphatase [*Prochlorococcus* phage P-SSM2] |  | 63.8% | 2.1E-61 |  |
| 46 | + | 28417 | 28587 | 57 | hypothetical protein BOW86_gp184 [*Synechococcus* phage S-CAM7] |  | 78.9% | 1.7E-23 |  |
|  |  |  |  |  | hypothetical protein [*Synechococcus* phage S-B68] |  | 62.8% | 1.7E-13 |  |
|  |  |  |  |  | hypothetical protein PQC15_gp246 [*Synechococcus* phage S-H34] |  | 62.5% | 1.1E-10 |  |
| 47 | + | 28580 | 28822 | 81 | hypothetical protein SWPG_00072 [*Synechococcus* phage S-CBM2] | glutaredoxin | 61.5% | 6.7E-30 | cd02066 (9.0E-05) |
|  |  |  |  |  | hypothetical protein SWZG_00233 [*Synechococcus* phage S-SKS1] |  | 51.3% | 2.4E-24 |  |
| 48 | + | 28819 | 29004 | 62 | hypothetical protein PQC15_gp171 [*Synechococcus* phage S-H34] |  | 81.7% | 6.9E-27 |  |
|  |  |  |  |  | hypothetical protein CPRG_00048 [*Synechococcus* phage Syn30] |  | 72.1% | 7.1E-22 |  |
|  |  |  |  |  | hypothetical protein PQC09_gp126 [*Synechococcus* phage S-N03] |  | 61.7% | 4.1E-20 |  |
|  |  |  |  |  | hypothetical protein [*Synechococcus* phage S-B68] |  | 65% | 5.0E-20 |  |
| 50 | + | 29167 | 29565 | 133 | hypothetical protein PQC15_gp173 [*Synechococcus* phage S-H34] |  | 73.2% | 1.7E-46 |  |
|  |  |  |  |  | hypothetical protein PQC09_gp128 [*Synechococcus* phage S-N03] |  | 72.2% | 9.3E-46 |  |
|  |  |  |  |  | hypothetical protein CC030809_00168 [*Synechococcus* phage S-CAM7] |  | 62.9% | 3.1E-36 |  |
|  |  |  |  |  | hypothetical protein [*Synechococcus* phage S-B68] |  | 61.2% | 2.6E-33 |  |
| 51 | + | 29672 | 30094 | 141 | hypothetical protein [*Synechococcus* phage S-B68] |  | 32.7% | 5.9E-07 |  |
| 52 | + | 30206 | 30514 | 103 | hypothetical protein PQC09_gp129 [*Synechococcus* phage S-N03] |  | 63.5% | 7.0E-17 |  |
|  |  |  |  |  | hypothetical protein PQC15_gp174 [*Synechococcus* phage S-H34] |  | 61.5% | 1.0E-15 |  |
| 53 | + | 30587 | 30817 | 77 | hypothetical protein [*Synechococcus* sp. WH 8016] |  | 42.7% | 6.3E-11 |  |
|  |  |  |  |  | hypothetical protein BOW86_gp029 [*Synechococcus* phage S-CAM7] |  | 53.1% | 3.9E-06 |  |
| 55 | + | 31017 | 33062 | 682 | peptidase [*Synechococcus* phage S-CRM01] |  | 35.6% | 2.1E-132 |  |
|  |  |  |  |  | peptidase [Cyanophage P-TIM40] |  | 34.2% | 2.5E-115 |  |
| 56 | + | 33141 | 34271 | 377 | porphyrin biosynthesis protein [*Brevundimonas* sp.] | MoxR-like ATPase | 61% | 2.7E-157 | COG0714 (1.6E-19) |
|  |  |  |  |  | ATPase [*Synechococcus* phage S-SCSM1] |  | 63.1% | 3.1E-157 |  |
|  |  |  |  |  | porphyrin biosynthesis [*Synechococcus* phage S-CRM01] |  | 61.8% | 1.2E-155 |  |
| 57 | + | 34271 | 34582 | 104 | hypothetical protein [*Synechococcus* phage S-B68] |  | 52.9% | 1.1E-22 |  |
|  |  |  |  |  | hypothetical protein PQC15_gp146 [*Synechococcus* phage S-H34] |  | 51% | 1.4E-21 |  |
|  |  |  |  |  | hypothetical protein PQC09_gp099 [*Synechococcus* phage S-N03] |  | 50.5% | 8.5E-21 |  |
| 59 | + | 34778 | 35560 | 261 | metallo-phosphoesterase [*Synechococcus* phage S-H34] | metallophosphoesterase | 47.5% | 2.6E-81 | pfam00149 (4.7E-08) |
|  |  |  |  |  | metallo-phosphoesterase [*Synechococcus* phage S-N03] |  | 45.4% | 1.5E-72 |  |
|  |  |  |  |  | metallo-phosphoesterase [*Synechococcus* phage S-CRM01] |  | 41.8% | 4.3E-60 |  |
|  |  |  |  |  | Ser/Thr protein phosphatase family protein [*Synechococcus* phage B3] |  | 39.9% | 3.7E-53 |  |
|  |  |  |  |  | Ser/Thr protein phosphatase family protein [*Synechococcus* phage S-B68] |  | 38.8% | 4.2E-53 |  |
| 60 | + | 35544 | 35774 | 77 | hypothetical protein PQC09_gp101 [*Synechococcus* phage S-N03] |  | 69.7% | 7.8E-32 |  |
|  |  |  |  |  | hypothetical protein [*Synechococcus* phage S-B68] |  | 57.9% | 8.1E-23 |  |
|  |  |  |  |  | hypothetical protein PQC15_gp149 [*Synechococcus* phage S-H34] |  | 71.1% | 4.3E-10 |  |
|  |  |  |  |  | hypothetical protein SShM2_022 [*Synechococcus* phage S-ShM2] |  | 40.9% | 6.0E-09 |  |
| 64 | + | 36212 | 36376 | 55 | hypothetical protein [*Synechococcus* phage S-B68] |  | 78.1% | 8.9E-13 |  |
| 65 | + | 36480 | 37244 | 255 | hypothetical protein [*Synechococcus* phage S-B68] | RNA polymerase sigma factor 70 | 71.5% | 1.5E-137 | COG0568 (1.7E-46) |
|  |  |  |  |  | RNA polymerase sigma factor [*Synechococcus* phage S-H34] |  | 71.2% | 2.0E-134 |  |
|  |  |  |  |  | subfamily RNA polymerase sigma-70 subunit [*Synechococcus* phage S-N03] |  | 71.2% | 2.2E-133 |  |
| 67 | + | 37570 | 37722 | 51 | hypothetical protein PQC15_gp128 [*Synechococcus* phage S-H34] |  | 78% | 9.0E-19 |  |
|  |  |  |  |  | hypothetical protein [*Synechococcus* phage S-B68] |  | 62.8% | 8.6E-11 |  |
|  |  |  |  |  | hypothetical protein PQC09_gp080 [*Synechococcus* phage S-N03] |  | 75% | 3.7E-10 |  |
| 72 | + | 38680 | 38886 | 69 | hypothetical protein [*Synechococcus* phage S-B68] |  | 70.2% | 1.1E-25 |  |
|  |  |  |  |  | hypothetical protein PQC15_gp125 [*Synechococcus* phage S-H34] |  | 62.1% | 7.5E-21 |  |
|  |  |  |  |  | hypothetical protein PQC09_gp077 [*Synechococcus* phage S-N03] |  | 62.3% | 1.5E-20 |  |
|  |  |  |  |  | hypothetical protein [*Synechococcus* phage S-B68] |  | 50.9% | 9.0E-13 |  |
| 75 | + | 39417 | 39683 | 89 | hypothetical protein PQC09_gp075 [*Synechococcus* phage S-N03] |  | 46.1% | 1.3E-14 |  |
|  |  |  |  |  | hypothetical protein PQC15_gp123 [*Synechococcus* phage S-H34] |  | 44.1% | 3.2E-11 |  |
| 76 | + | 39683 | 39868 | 62 | hypothetical protein PQC15_gp077 [*Synechococcus* phage S-H34] |  | 48.3% | 1.2E-08 |  |
|  |  |  |  |  | hypothetical protein PQC09_gp025 [*Synechococcus* phage S-N03] |  | 43.1% | 1.2E-06 |  |
| 78 | + | 40085 | 40294 | 70 | hypothetical protein [*Synechococcus* phage S-B68] |  | 63% | 3.7E-15 |  |
|  |  |  |  |  | hypothetical protein PQC09_gp073 [*Synechococcus* phage S-N03] |  | 58.2% | 9.7E-15 |  |
|  |  |  |  |  | hypothetical protein PQC15_gp121 [*Synechococcus* phage S-H34] |  | 50% | 2.2E-13 |  |
| 79 | + | 40296 | 40835 | 180 | hemagglutinin [*Synechococcus* phage S-H34] |  | 64.1% | 1.7E-71 |  |
|  |  |  |  |  | hemagglutinin [*Synechococcus* phage S-N03] |  | 61.1% | 9.3E-69 |  |
|  |  |  |  |  | hemagglutinin domain-containing protein [*Synechococcus* phage S-B68] |  | 58.7% | 3.6E-64 |  |
|  |  |  |  |  | hemagglutinin [*Synechococcus* phage S-CBS2] |  | 50.9% | 4.4E-43 |  |
| 80 | + | 40907 | 41140 | 78 | hypothetical protein PQC11_gp030 [*Synechococcus* phage S-H9-1] |  | 51.3% | 2.7E-20 |  |
|  |  |  |  |  | hypothetical protein BJD26_gp185 [Cyanophage S-RIM32] |  | 51.9% | 1.5E-19 |  |
| 81 | + | 41211 | 41387 | 59 | hypothetical protein BOW85_gp201 [*Synechococcus* phage S-CAM9] |  | 54.6% | 6.7E-13 |  |
|  |  |  |  |  | hypothetical protein [*Synechococcus* phage S-H25] |  | 51.9% | 4.7E-12 |  |
|  |  |  |  |  | hypothetical protein PQC15_gp079 [*Synechococcus* phage S-H34] |  | 44.4% | 8.4E-10 |  |
| 83 | + | 41765 | 42115 | 117 | two-component sensor histidine kinase [*Synechococcus* phage ACG-2014f_Syn7803C7] |  | 45.3% | 1.5E-25 |  |
|  |  |  |  |  | hypothetical protein PQC09_gp132 [*Synechococcus* phage S-N03] |  | 32.6% | 4.4E-09 |  |
|  |  |  |  |  | hypothetical protein PQC15_gp175 [*Synechococcus* phage S-H34] |  | 32.6% | 5.7E-09 |  |
| 84 | + | 42165 | 42554 | 130 | hypothetical protein PQC15_gp046 [*Synechococcus* phage S-H34] |  | 47.5% | 1.3E-28 |  |
|  |  |  |  |  | hypothetical protein PQC09_gp190 [*Synechococcus* phage S-N03] |  | 42.5% | 2.5E-24 |  |
|  |  |  |  |  | hypothetical protein [*Synechococcus* phage S-B68] |  | 43.6% | 8.4E-21 |  |
|  |  |  |  |  | hypothetical cyanophage protein [*Synechococcus* phage S-CRM01] |  | 38.7% | 7.7E-16 |  |
|  |  |  |  |  | unknown function [*Synechococcus* phage S-SRM01] |  | 31% | 8.2E-08 |  |
| 85 | + | 42559 | 57474 | 4972 | tail fiber protein [*Synechococcus* phage S-B68] | long tail fiber protein | 61.2% | 2.5E-127 | PHA02584 ( 5.2E-06) |
|  |  |  |  |  | tail fiber protein [*Synechococcus* phage S-H34] |  | 72.4% | 6.5E-68 |  |
| 86 | + | 57474 | 58481 | 336 | hypothetical protein HWB27_gp50 [*Lentibacter* phage vB_LenP_ICBM1] |  | 60% | 2.6E-09 |  |
| 87 | + | 58890 | 59777 | 296 | hypothetical protein PQC15_gp108 [*Synechococcus* phage S-H34] |  | 54.6% | 6.0E-99 |  |
|  |  |  |  |  | hypothetical protein PQC09_gp061 [*Synechococcus* phage S-N03] |  | 54.6% | 1.6E-98 |  |
|  |  |  |  |  | hypothetical protein [*Synechococcus* phage S-B68] |  | 49.7% | 1.9E-89 |  |
|  |  |  |  |  | hypothetical protein [*Synechococcus* phage S-B05] |  | 48.1% | 1.8E-87 |  |
| 88 | + | 59777 | 59980 | 68 | hypothetical protein [uncultured Mediterranean phage uvMED] |  | 65.5% | 1.2E-17 |  |
|  |  |  |  |  | hypothetical protein CYVG_00141 [Cyanophage S-SSM6a] |  | 60.4% | 1.0E-15 |  |
|  |  |  |  |  | hypothetical protein PQC15_gp228 [*Synechococcus* phage S-H34] |  | 52.1% | 8.6E-10 |  |
| 96 | + | 61903 | 62496 | 198 | hypothetical protein [*Synechococcus* phage S-B68] |  | 60% | 3.8E-23 |  |
| 97 | + | 62580 | 62744 | 55 | hypothetical protein HOQ62_gp097 [*Synechococcus* phage ACG-2014f_Syn7803C8] |  | 41.6% | 4.8E-07 |  |
| 105 | + | 64886 | 65161 | 92 | hypothetical protein [*Kofleriaceae* bacterium] |  | 51% | 3.6E-08 |  |
| 108 | + | 65598 | 66203 | 202 | hypothetical protein PQC15_gp117 [*Synechococcus* phage S-H34] |  | 43.1% | 3.4E-38 |  |
|  |  |  |  |  | hypothetical protein PQC09_gp069 [*Synechococcus* phage S-N03] |  | 41.9% | 1.7E-26 |  |
|  |  |  |  |  | hypothetical protein [*Synechococcus* phage S-B68] |  | 35.2% | 2.2E-18 |  |
|  |  |  |  |  | hypothetical protein [*Synechococcus* phage S-B43] |  | 34.7% | 2.9E-17 |  |
| 109 | + | 66816 | 67268 | 151 | hypothetical protein PQC09_gp018 [*Synechococcus* phage S-N03] | S-type phycobilin lyase (*cpcV*) | 73.6% | 1.8E-71 | cd16339 (1E-05) |
|  |  |  |  |  | hypothetical protein PQC15_gp070 [*Synechococcus* phage S-H34] |  | 72.2% | 6.2E-67 |  |
|  |  |  |  |  | hypothetical protein [*Synechococcus* phage S-B68] |  | 66.9% | 4.6E-63 |  |
| 111 | + | 67610 | 68431 | 274 | hypothetical protein [*Synechococcus* phage S-B68] |  | 71.4% | 6.6E-15 |  |
|  |  |  |  |  | hypothetical protein SBM1_00083 [*Synechococcus* phage S-BM1] |  | 66.1% | 3.6E-11 |  |
|  |  |  |  |  | hypothetical protein PQC15_gp068 [*Synechococcus* phage S-H34] |  | 44.6% | 3.2E-10 |  |
| 112 | + | 68500 | 69726 | 409 | hypothetical protein PQC09_gp191 [*Synechococcus* phage S-N03] |  | 45.6% | 2.7E-16 |  |
|  |  |  |  |  | hypothetical protein PQC15_gp045 [*Synechococcus* phage S-H34] |  | 43.3% | 6.8E-15 |  |
|  |  |  |  |  | virion structural protein [*Synechococcus* phage S-CAM4] |  | 31.3% | 9.0E-09 |  |
| 113 | + | 69756 | 70094 | 113 | hypothetical protein [*Alteromonadales* bacterium] |  | 50.7% | 3.2E-10 |  |
| 114 | + | 70167 | 70994 | 276 | hypothetical protein [*Bacteroidota* bacterium] |  | 64.6% | 4.5E-09 |  |
| 115 | + | 71020 | 73407 | 796 | hypothetical protein [*Rhizorhabdus* sp.] |  | 49.6% | 1.7E-22 |  |
|  |  |  |  |  | hypothetical protein PQC09_gp191 [*Synechococcus* phage S-N03] |  | 44.8% | 1.9E-16 |  |
|  |  |  |  |  | hypothetical protein PQC15_gp045 [*Synechococcus* phage S-H34] |  | 45.8% | 3.6E-16 |  |
|  |  |  |  |  | hypothetical protein [*Synechococcus* phage S-B68] |  | 34.1% | 1.1E-08 |  |
| 117 | + | 74401 | 74859 | 153 | hypothetical protein [*Bacteroidota* bacterium] |  | 46.8% | 1.1E-35 |  |
| 118 | + | 74911 | 75108 | 66 | hypothetical protein PQC09_gp004 [*Synechococcus* phage S-N03] | tail assembly chaperone | 76.9% | 1.6E-30 | pfam16778 (3.7E-12) |
|  |  |  |  |  | hypothetical protein [*Synechococcus* phage S-B68] |  | 78.1% | 2.1E-30 |  |
|  |  |  |  |  | hypothetical protein PQC15_gp056 [*Synechococcus* phage S-H34] |  | 76.6% | 8.1E-30 |  |
|  |  |  |  |  | hypothetical protein SCRM01_293 [*Synechococcus* phage S-CRM01] |  | 52.5% | 1.1E-14 |  |
| 120 | + | 75282 | 75689 | 136 | baseplate wedge subunit [*Synechococcus* phage S-N03] | baseplate wedge protein, inner | 60.2% | 4.0E-53 | PHA00415 (1.8E-28) |
|  |  |  |  |  | baseplate wedge subunit [*Synechococcus* phage S-H34] |  | 58.7% | 4.7E-50 |  |
|  |  |  |  |  | hypothetical protein [*Synechococcus* phage S-B68] |  | 56.5% | 2.0E-46 |  |
|  |  |  |  |  | baseplate wedge subunit [*Synechococcus* phage S-CRM01] |  | 57.3% | 1.2E-44 |  |
|  |  |  |  |  | baseplate wedge subunit [*Synechococcus* phage S-SRM01] |  | 47.4% | 3.1E-30 |  |
| 121 | + | 75723 | 77597 | 625 | baseplate wedge subunit [*Synechococcus* phage S-B68] | baseplate wedge protein, inner | 54.7% | 0 | PHA02553 (5.3E-118) |
|  |  |  |  |  | baseplate wedge subunit [*Synechococcus* phage S-H34] |  | 56% | 0 |  |
|  |  |  |  |  | baseplate wedge subunit [*Synechococcus* phage S-N03] |  | 55.5% | 0 |  |
|  |  |  |  |  | baseplate wedge subunit [*Synechococcus* phage S-CRM01] |  | 51.7% | 0 |  |
|  |  |  |  |  | baseplate wedge [*Synechococcus* phage S-BM3] |  | 38.2% | 2.2E-127 |  |
| 122 | + | 77594 | 83407 | 1938 | hypothetical protein [*Synechococcus* phage S-B68] | baseplate wedge protein, intermediate | 40.9% | 0 | pfam14240 (6.4E-18) |
|  |  |  |  |  | baseplate wedge subunit [*Synechococcus* phage S-H34] |  | 40% | 0 |  |
|  |  |  |  |  | virion structural protein [*Synechococcus* phage S-PM2] |  | 27% | 6.6E-88 |  |
| 123 | + | 83440 | 84939 | 500 | baseplate wedge subunit [*Synechococcus* phage S-H34] | baseplate wedge protein, intermediate | 41.2% | 7.8E-111 | PRK15315 (4.5E-04) |
|  |  |  |  |  | baseplate wedge subunit [*Synechococcus* phage S-N03] |  | 39.4% | 6.8E-105 |  |
|  |  |  |  |  | baseplate wedge subunit [*Synechococcus* phage S-B68] |  | 38.6% | 1.3E-103 |  |
|  |  |  |  |  | baseplate wedge subunit [*Synechococcus* phage S-CRM01] |  | 35.2% | 2.1E-91 |  |
|  |  |  |  |  | baseplate wedge subunit [*Synechococcus* phage S-H38] |  | 28.5% | 2.5E-40 |  |
| 124 | + | 84976 | 92982 | 2669 | hypothetical protein [*Synechococcus* phage S-B68] |  | 46.4% | 0 |  |
|  |  |  |  |  | structural protein [*Synechococcus* phage S-H34] |  | 49.1% | 0 |  |
|  |  |  |  |  | structural protein [*Synechococcus* phage S-N03] |  | 47.9% | 0 |  |
|  |  |  |  |  | structural protein [*Synechococcus* phage S-CRM01] |  | 43.1% | 0 |  |
|  |  |  |  |  | virulence associated protein [*Synechococcus* phage S-BM3] |  | 31.8% | 0 |  |
| 125 | + | 93020 | 93286 | 89 | hypothetical protein [*Synechococcus* phage S-B68] |  | 45.6% | 2.6E-12 |  |
|  |  |  |  |  | virion structural protein [*Synechococcus* phage S-CRM01] |  | 38.8% | 2.6E-08 |  |
|  |  |  |  |  | virion structural protein [*Synechococcus* phage S-N03] |  | 41.8% | 5.6E-06 |  |
| 126 | + | 93309 | 94577 | 423 | tail fiber protein [*Synechococcus* phage S-B68] | short tail fiber protein | 52.5% | 5.6E-143 | pfam07484 (1.8E-22) |
|  |  |  |  |  | tail fiber protein; Ig-domain containing [*Synechococcus* phage S-N03] |  | 49.4% | 1.9E-133 |  |
|  |  |  |  |  | tail fiber protein; Ig-domain containing [*Synechococcus* phage S-H34] |  | 47.7% | 6.5E-131 |  |
|  |  |  |  |  | tail collar fiber protein [*Synechococcus* phage S-CRM01] |  | 39.3% | 1.1E-88 |  |
| 127 | + | 94606 | 94944 | 113 | hypothetical protein [*Synechococcus* phage S-B68] |  | 37.5% | 9.9E-12 |  |
|  |  |  |  |  | hypothetical protein PQC15_gp047 [*Synechococcus* phage S-H34] |  | 36.2% | 3.2E-06 |  |
|  |  |  |  |  | hypothetical protein PQC09_gp189 [*Synechococcus* phage S-N03] |  | 34.6% | 7.1E-06 |  |
| 128 | + | 94985 | 96814 | 610 | hypothetical protein PQC15_gp045 [*Synechococcus* phage S-H34] |  | 39.3% | 1.9E-38 |  |
|  |  |  |  |  | hypothetical protein PQC09_gp191 [*Synechococcus* phage S-N03] |  | 38.3% | 1.0E-36 |  |
|  |  |  |  |  | hypothetical protein [*Synechococcus* phage S-B68] |  | 30.9% | 3.7E-36 |  |
| 129 | + | 97032 | 98582 | 517 | hypothetical protein [*Synechococcus* phage S-B68] |  | 47.8% | 7.1E-138 |  |
|  |  |  |  |  | hypothetical protein PQC15_gp002 [*Synechococcus* phage S-H34] |  | 53.6% | 5.3E-130 |  |
| 130 | + | 98589 | 103511 | 1641 | hypothetical protein [*Synechococcus* phage S-B68] |  | 54.1% | 0 |  |
|  |  |  |  |  | tail protein [*Synechococcus* phage S-H34] |  | 51.9% | 0 |  |
|  |  |  |  |  | fiber Ig/hemolysin [Cyanophage PSS2] |  | 29.9% | 2.1E-91 |  |
| 131 | + | 103579 | 103773 | 65 | hypothetical protein [*Synechococcus* phage S-B68] |  | 74.6% | 1.5E-15 |  |
| 132 | + | 103792 | 104919 | 376 | head completion, neck hetero-dimeric protein [*Synechococcus* phage S-B68] | adaptor | 68.4% | 0 | PHA02554 (5.7E-33) |
|  |  |  |  |  | head-tail adaptor Ad2 [*Synechococcus* phage S-N03] |  | 60.3% | 1.1E-150 |  |
|  |  |  |  |  | neck protein [*Synechococcus* phage S-CRM01] |  | 49.2% | 4.3E-132 |  |
|  |  |  |  |  | head-tail adaptor Ad2 [*Synechococcus* phage S-H34] |  | 72.3% | 1.6E-29 |  |
|  |  |  |  |  | head-tail adaptor Ad2 [*Synechococcus* phage S-CAM9] |  | 29.4% | 2.8E-43 |  |
| 133 | + | 104922 | 105965 | 348 | head completion protein [*Synechococcus* phage S-B68] | stopper | 61.7% | 9.3E-154 | PHA02555 (2.8E-54) |
|  |  |  |  |  | head closure Hc2 [*Synechococcus* phage S-H34] |  | 60% | 5.7E-145 |  |
|  |  |  |  |  | head closure Hc2 [*Synechococcus* phage S-N03] |  | 60.4% | 1.8E-144 |  |
|  |  |  |  |  | head closure Hc2 [*Synechococcus* phage S-CRM01] |  | 47.6% | 4.1E-108 |  |
|  |  |  |  |  | head closure Hc2 [*Synechococcus* phage S-SSM4] |  | 33.4% | 5.7E-48 |  |
| 134 | + | 105994 | 106821 | 276 | tail completion protein [*Synechococcus* phage S-B68] | sheath terminator | 72% | 2.2E-151 | PHA02556 (3.9E-81) |
|  |  |  |  |  | tail sheath stabilizer [*Synechococcus* phage S-CRM01] |  | 66.4% | 6.4E-135 |  |
|  |  |  |  |  | tail sheath stabilizer [*Synechococcus* phage S-H34] |  | 64.9% | 5.0E-131 |  |
|  |  |  |  |  | tail sheath stabilizer [*Synechococcus* phage S-N03] |  | 65.2% | 5.1E-129 |  |
|  |  |  |  |  | tail sheath stabilizer [Cyanophage S-RIM50] |  | 49.2% | 1.2E-69 |  |
| 135 | + | 106821 | 107282 | 154 | terminase small subunit [*Synechococcus* phage S-B68] |  | 64.1% | 5.0E-60 |  |
|  |  |  |  |  | terminase small subunit [*Synechococcus* phage S-N03] |  | 66.7% | 8.8E-60 |  |
|  |  |  |  |  | terminase small subunit [*Synechococcus* phage S-H34] |  | 62.7% | 5.8E-58 |  |
|  |  |  |  |  | terminase small subunit [*Synechococcus* phage S-WAM2] |  | 42.9% | 3.9E-25 |  |
|  |  |  |  |  | terminase small subunit [*Synechococcus* phage S-CRM01] |  | 39.3% | 1.7E-20 |  |
| 137 | + | 108049 | 109521 | 491 | hypothetical protein PQC15_gp031 [*Synechococcus* phage S-H34] |  | 49.6% | 4.8E-160 |  |
|  |  |  |  |  | hypothetical protein PQC09_gp205 [*Synechococcus* phage S-N03] |  | 43.5% | 1.0E-134 |  |
|  |  |  |  |  | hypothetical protein [*Synechococcus* phage S-B68] |  | 42.4% | 3.6E-129 |  |
|  |  |  |  |  | hypothetical cyanophage protein [*Synechococcus* phage S-CRM01] |  | 37.2% | 2.6E-77 |  |
|  |  |  |  |  | hypothetical protein b3_0273 [*Synechococcus* phage B3] |  | 79.3% | 5.6E-37 |  |
| 138 | + | 109660 | 111345 | 562 | terminase large subunit [*Synechococcus* phage S-B68] | large terminase subunit | 86.8% | 0 | PHA02533 (0) |
|  |  |  |  |  | terminase large subunit [*Synechococcus* phage S-H34] |  | 85.9% | 0 |  |
|  |  |  |  |  | terminase large subunit [*Synechococcus* phage S-N03] |  | 83.8% | 0 |  |
|  |  |  |  |  | terminase large subunit [*Synechococcus* phage S-CRM01] |  | 75.5% | 0 |  |
|  |  |  |  |  | terminase large subunit [*Synechococcus* phage S-SM2] |  | 65.6% | 0 |  |
| 139 | + | 111494 | 113878 | 795 | tail sheath monomer [*Synechococcus* phage S-B68] | tail sheath protein | 65.2% | 0 | PHA02539 (0) |
|  |  |  |  |  | tail sheath [*Synechococcus* phage S-N03] |  | 61.6% | 0 |  |
|  |  |  |  |  | tail sheath [*Synechococcus* phage S-H34] |  | 61.1% | 0 |  |
|  |  |  |  |  | tail sheath [*Synechococcus* phage S-CRM01] |  | 57.7% | 0 |  |
|  |  |  |  |  | tail sheath monomer [*Synechococcus* phage S-BM3] |  | 41.8% | 0 |  |
| 140 | + | 113911 | 114510 | 200 | tail tube [*Synechococcus* phage S-H34] | tail tube protein | 67.8% | 6.4E-96 | PHA02551 (6.1E-57) |
|  |  |  |  |  | tail tube [*Synechococcus* phage S-N03] |  | 67.3% | 8.4E-95 |  |
|  |  |  |  |  | tail tube protein [*Synechococcus* phage S-B68] |  | 67.4% | 1.0E-88 |  |
|  |  |  |  |  | T4-like tail tube protein [*Synechococcus* phage S-CRM01] |  | 66.7% | 1.3E-88 |  |
|  |  |  |  |  | tail tube [*Synechococcus* phage S-SKS1] |  | 42.5% | 3.3E-48 |  |
| 141 | + | 114599 | 116422 | 608 | portal vertex of the head [*Synechococcus* phage S-B68] | portal protein | 75.6% | 0 | PHA02531 (0) |
|  |  |  |  |  | portal protein [*Synechococcus* phage S-H34] |  | 75.8% | 0 |  |
|  |  |  |  |  | portal protein [*Synechococcus* phage S-N03] |  | 74.7% | 0 |  |
|  |  |  |  |  | portal protein [*Synechococcus* phage S-CRM01] |  | 67.4% | 0 |  |
|  |  |  |  |  | T4gp20 homolog [Cyanophage S-BnM1] |  | 56% | 0 |  |
| 142 | + | 116454 | 116639 | 62 | hypothetical protein PQC15_gp024 [*Synechococcus* phage S-H34] |  | 82.5% | 4.2E-26 |  |
|  |  |  |  |  | hypothetical protein PQC09_gp211 [*Synechococcus* phage S-N03] |  | 80.7% | 3.3E-25 |  |
|  |  |  |  |  | hypothetical protein [*Synechococcus* phage S-B68] |  | 76.4% | 9.1E-22 |  |
|  |  |  |  |  | hypothetical protein SCRM01_040 [*Synechococcus* phage S-CRM01] |  | 64.2% | 1.2E-16 |  |
| 143 | + | 116656 | 117297 | 214 | head maturation protease [*Synechococcus* phage S-H34] | prohead core protein protease | 92.9% | 5.3E-144 | PHA00911 (2.2E-82) |
|  |  |  |  |  | head maturation protease [*Synechococcus* phage S-N03] |  | 92.5% | 1.6E-143 |  |
|  |  |  |  |  | prohead core protein [*Synechococcus* phage S-B68] |  | 90.6% | 5.7E-141 |  |
|  |  |  |  |  | head maturation protease [*Synechococcus* phage S-CRM01] |  | 72.9% | 2.1E-106 |  |
|  |  |  |  |  | head maturation protease [*Prochlorococcus* phage Syn33] |  | 65.3% | 8.2E-96 |  |
| 144 | + | 117378 | 118436 | 353 | head scaffolding protein [*Synechococcus* phage S-N03] | capsid assembly scaffolding protein | 80.8% | 0 | PHA02557 (1.8E-47) |
|  |  |  |  |  | prohead assembly (scaffolding) protein [*Synechococcus* phage S-B68] |  | 77.3% | 0 |  |
|  |  |  |  |  | head scaffolding protein [*Synechococcus* phage S-H34] |  | 81.4% | 8.0E-178 |  |
|  |  |  |  |  | head scaffolding protein [*Synechococcus* phage S-CRM01] |  | 58.6% | 2.1E-136 |  |
|  |  |  |  |  | scaffold prohead core protein [*Synechococcus* phage S-BM3] |  | 43.1% | 5.4E-78 |  |
| 145 | + | 118481 | 119851 | 457 | major capsid protein [*Synechococcus* phage S-B68] | major capsid protein | 77.6% | 0 | PHA02541 (0) |
|  |  |  |  |  | major head protein [*Synechococcus* phage S-H34] |  | 76.5% | 0 |  |
|  |  |  |  |  | major head protein [*Synechococcus* phage S-N03] |  | 75.6% | 0 |  |
|  |  |  |  |  | major head protein [*Synechococcus* phage S-CRM01] |  | 72.5% | 0 |  |
|  |  |  |  |  | major head protein [*Synechococcus* phage S-SCSM1] |  | 64.9% | 0 |  |
| 146 | + | 119959 | 120438 | 160 | homing endonuclease [*Synechococcus* phage S-H34] | intron-encoded endonuclease bI1 | 45.4% | 7.8E-42 | cd10445 (1.3E-16) |
|  |  |  |  |  | homing endonuclease [*Synechococcus* phage S-N03] |  | 35.2% | 5.3E-16 |  |
| 147 | + | 120464 | 121129 | 222 | tail completion protein [*Synechococcus* phage S-B68] | distal tail protein | 63.8% | 8.5E-94 | PHA02576 (1.6E-12) |
|  |  |  |  |  | tail tube [*Synechococcus* phage S-H34] |  | 57.3% | 1.4E-85 |  |
|  |  |  |  |  | tail completion and sheath stabilizer [*Synechococcus* phage S-CRM01] |  | 57.9% | 1.2E-84 |  |
|  |  |  |  |  | tail tube [*Synechococcus* phage S-N03] |  | 55.5% | 9.5E-84 |  |
|  |  |  |  |  | head-proximal tip of tail tube tail completion + sheath stabilizer protein [*Synechococcus* phage S-BM3] |  | 44.2% | 1.5E-46 |  |
| 148 | + | 121143 | 121568 | 142 | UvsY [*Synechococcus* phage S-B68] | recombination, repair and ssDNA binding protein UvsY | 66% | 6.9E-66 | pfam11056 (3.9E-29) |
|  |  |  |  |  | UvsY-like recombination mediator [*Synechococcus* phage S-H34] |  | 67.4% | 1.6E-65 |  |
|  |  |  |  |  | UvsY-like recombination mediator [*Synechococcus* phage S-N03] |  | 66% | 5.7E-65 |  |
|  |  |  |  |  | UvsY-like recombination mediator [*Synechococcus* phage S-CRM01] |  | 60% | 1.2E-56 |  |
|  |  |  |  |  | UvsY-like recombination mediator [*Synechococcus* phage S-ShM2] |  | 44.4% | 1.5E-34 |  |
| 149 | + | 121558 | 121785 | 76 | hypothetical protein PQC15_gp017 [*Synechococcus* phage S-H34] |  | 40.3% | 2.0E-06 |  |
| 150 | + | 121782 | 121979 | 66 | hypothetical protein PQC15_gp016 [*Synechococcus* phage S-H34] |  | 56.3% | 3.6E-10 |  |
|  |  |  |  |  | hypothetical protein PQC09_gp219 [*Synechococcus* phage S-N03] |  | 54.8% | 4.7E-07 |  |
| 151 | + | 122030 | 123517 | 496 | RNA-DNA + DNA-DNA helicase [*Synechococcus* phage S-N03] | UvsW DNA helicase | 88.2% | 0 | PHA02558 (0) |
|  |  |  |  |  | RNA-DNA + DNA-DNA helicase [*Synechococcus* phage S-H34] |  | 87.5% | 0 |  |
|  |  |  |  |  | RNA-DNA + DNA-DNA helicase [*Synechococcus* phage S-B68] |  | 84.6% | 0 |  |
|  |  |  |  |  | DNA helicase [*Synechococcus* phage S-CRM01] |  | 68.9% | 0 |  |
|  |  |  |  |  | DNA helicase [*Synechococcus* phage S-SSM7] |  | 53.7% | 0 |  |
| 152 | + | 123514 | 123930 | 139 | hypothetical protein [*Synechococcus* phage S-B68] | Sm-like RNA-binding protein | 51.5% | 1.3E-44 | pfam16243 (7.3E-06) |
|  |  |  |  |  | methylamine utilization [*Synechococcus* phage S-CRM01] |  | 49.3% | 8.2E-41 |  |
|  |  |  |  |  | methylamine utilization [*Synechococcus* phage S-H34] |  | 40.4% | 1.2E-30 |  |
|  |  |  |  |  | methylamine utilization [*Synechococcus* phage S-N03] |  | 43.2% | 2.1E-30 |  |
|  |  |  |  |  | hypothetical protein SBM3_00157 [*Synechococcus* phage S-BM3] |  | 29% | 7.9E-13 |  |
| 153 | + | 124051 | 124536 | 162 | RNA polymerase sigma factor for late transcription [*Synechococcus* phage S-B68] | RNA polymerase sigma factor | 85.5% | 6.6E-101 | PHA02547 (2.9E-52) |
|  |  |  |  |  | sigma factor for late transcription [*Synechococcus* phage S-N03] |  | 82.5% | 2.2E-97 |  |
|  |  |  |  |  | sigma factor for late transcription [*Synechococcus* phage S-H34] |  | 81.9% | 1.4E-94 |  |
|  |  |  |  |  | late sigma transcription factor [*Synechococcus* phage S-CRM01] |  | 68.2% | 1.7E-77 |  |
|  |  |  |  |  | sigma factor for late transcription [*Synechococcus* phage S-SZBM1] |  | 54.3% | 9.6E-58 |  |
| 154 | + | 124608 | 125468 | 287 | hypothetical protein [*Synechococcus* phage S-B68] | cytidylyltransferase | 68.6% | 5.5E-141 | cd02039 (7E-06) |
|  |  |  |  |  | cytidyltransferase [*Synechococcus* phage S-N03] |  | 65.9% | 1.8E-131 |  |
|  |  |  |  |  | cytidyltransferase [*Synechococcus* phage S-H34] |  | 65.8% | 3.4E-131 |  |
|  |  |  |  |  | cytidyltransferase [*Synechococcus* phage S-CRM01] |  | 52.7% | 1.7E-98 |  |
|  |  |  |  |  | cytidyltransferase [*Prochlorococcus* phage P-SSM2] |  | 38.3% | 4.8E-57 |  |
| 155 | + | 125491 | 126012 | 174 | hypothetical protein [*Synechococcus* phage S-B68] |  | 59.5% | 1.8E-07 |  |
| 156 | + | 126012 | 126725 | 238 | hypothetical protein PQC15_gp009 [*Synechococcus* phage S-H34] |  | 82.2% | 1.9E-137 |  |
|  |  |  |  |  | hypothetical protein PQC09_gp226 [*Synechococcus* phage S-N03] |  | 80.5% | 1.4E-135 |  |
|  |  |  |  |  | hypothetical protein [*Synechococcus* phage S-B68] |  | 78.1% | 5.8E-130 |  |
|  |  |  |  |  | hypothetical protein SCRM01_125c [*Synechococcus* phage S-CRM01] |  | 63.4% | 1.2E-99 |  |
|  |  |  |  |  | aminoglycoside nucleotidyltransferase [*Synechococcus* phage DSL-LC02] |  | 53.5% | 1.1E-76 |  |
| 157 | + | 126795 | 127385 | 197 | hypothetical protein [*Euryarchaeota* *archaeon*] |  | 36.9% | 6.8E-38 |  |
|  |  |  |  |  | hypothetical protein S-CBP2_0046 [*Synechococcus* phage S-CBP2] |  | 31.9% | 3.3E-32 |  |
| 158 | + | 127420 | 127950 | 177 | hypothetical protein PQC15_gp008 [*Synechococcus* phage S-H34] |  | 66.1% | 1.4E-69 |  |
|  |  |  |  |  | hypothetical protein PQC09_gp227 [*Synechococcus* phage S-N03] |  | 51.1% | 9.1E-18 |  |
|  |  |  |  |  | hypothetical protein [*Synechococcus* phage S-B68] |  | 50.8% | 8.0E-12 |  |
|  |  |  |  |  | S-layer domain protein [*Synechococcus* phage S-SM1] |  | 62.5% | 6.3E-06 |  |
| 159 | + | 127971 | 128342 | 124 | hypothetical protein [*Synechococcus* phage S-B68] |  | 74.8% | 1.4E-63 |  |
|  |  |  |  |  | hypothetical protein PQC09_gp228 [*Synechococcus* phage S-N03] |  | 70.7% | 2.8E-62 |  |
|  |  |  |  |  | hypothetical protein PQC15_gp007 [*Synechococcus* phage S-H34] |  | 69.9% | 1.6E-59 |  |
|  |  |  |  |  | hypothetical cyanophage protein [*Synechococcus* phage S-CRM01] |  | 68.9% | 5.9E-57 |  |
|  |  |  |  |  | hypothetical protein [*Synechococcus* phage S-H68] |  | 52.9% | 9.0E-43 |  |
| 160 | + | 128342 | 129235 | 298 | hypothetical protein PQC15_gp006 [*Synechococcus* phage S-H34] |  | 56.9% | 4.5E-94 |  |
|  |  |  |  |  | structural protein [*Synechococcus* phage S-B68] |  | 73.7% | 9.2E-69 |  |
|  |  |  |  |  | hypothetical protein SWZG_00153 [*Synechococcus* phage S-SKS1] |  | 66.3% | 6.9E-64 |  |
| 161 | + | 129354 | 129788 | 145 | hypothetical protein [*Flavobacteriaceae* bacterium] | UvrC | 52.7% | 2.0E-18 | cd10440 (1.8E-07) |
|  |  |  |  |  | hypothetical protein PQC13_gp035 [*Synechococcus* phage S-SRM01] |  | 43.4% | 3.5E-13 |  |
|  |  |  |  |  | hypothetical protein PQC09_gp201 [*Synechococcus* phage S-N03] |  | 39% | 1.3E-09 |  |
|  |  |  |  |  | hypothetical protein [*Synechococcus* phage S-B68] |  | 39.1% | 1.6E-08 |  |
| 162 | + | 129837 | 130832 | 332 | hypothetical protein [*Synechococcus* phage S-B68] | DNA primase | 68% | 9.2E-171 | PHA02540 (4.2E-127) |
|  |  |  |  |  | DNA primase [*Synechococcus* phage S-H34] |  | 68.5% | 5.9E-170 |  |
|  |  |  |  |  | DNA primase [*Synechococcus* phage S-N03] |  | 68.5% | 7.4E-168 |  |
|  |  |  |  |  | DNA primase [*Synechococcus* phage S-CRM01] |  | 55.9% | 1.0E-128 |  |
|  |  |  |  |  | DNA primase [*Synechococcus* phage S-WAM2] |  | 49.3% | 6.9E-106 |  |
| 163 | + | 130829 | 133126 | 766 | ribonucleotide reductase [*Synechococcus* phage S-N03] | ribonucleotide reductase of class Ia (aerobic), alpha subunit (*nrdA*） | 85.9% | 0 | PHA02572 (0) |
|  |  |  |  |  | ribonucleotide reductase [*Synechococcus* phage S-H34] |  | 86.8% | 0 |  |
|  |  |  |  |  | ribonucleotide reductase of class Ia (aerobic), alpha subunit [*Synechococcus* phage S-B68] |  | 82.2% | 0 |  |
|  |  |  |  |  | ribonucleotide reductase [*Synechococcus* phage S-SRM01] |  | 71.5% | 0 |  |
|  |  |  |  |  | ribonucleotide reductase large subunit [*Synechococcus* phage S-CRM01] |  | 68.7% | 0 |  |
| 164 | + | 133126 | 134205 | 360 | ribonucleotide reductase of class Ia (aerobic), beta subunit [*Synechococcus* phage S-B68] | ribonucleotide reductase of class Ia (aerobic), beta subunit (*nrdB*） | 86.1% | 0 | COG0208 (7.4E-84) |
|  |  |  |  |  | ribonucleotide reductase class Ia beta subunit [*Synechococcus* phage S-H34] |  | 84.4% | 0 |  |
|  |  |  |  |  | ribonucleotide reductase class Ia beta subunit [*Synechococcus* phage S-N03] |  | 83.8% | 0 |  |
|  |  |  |  |  | ribonucleoside diphosphate reductase small subunit [*Synechococcus* phage S-CRM01] |  | 72.4% | 0 |  |
| 166 | + | 134753 | 135070 | 106 | hypothetical protein [Cyanophage S-TIM54] |  | 65.7% | 2.3E-45 |  |
| 167 | + | 135102 | 135446 | 115 | RusA-like Holliday junction resolvase [*Synechococcus* phage S-H34] |  | 94.6% | 8.3E-72 |  |
|  |  |  |  |  | hypothetical protein [*Synechococcus* phage S-B68] |  | 81.4% | 1.1E-62 |  |
|  |  |  |  |  | RusA-like Holliday junction resolvase [*Synechococcus* phage S-N03] |  | 83% | 1.7E-60 |  |
|  |  |  |  |  | RusA-like Holliday junction resolvase [*Synechococcus* phage DSL-LC03] |  | 65.5% | 3.4E-47 |  |
| 168 | + | 135439 | 135969 | 177 | endolysin [*Synechococcus* phage S-B68] | C39 family peptidases | 61.5% | 3.5E-71 | cd02549 (1.1E-08) |
|  |  |  |  |  | endolysin [*Synechococcus* phage S-H34] |  | 59.7% | 1.9E-67 |  |
|  |  |  |  |  | endolysin [*Synechococcus* phage S-N03] |  | 59.7% | 4.5E-67 |  |
|  |  |  |  |  | C39 family peptidase [*Synechococcus* phage DSL-LC02] |  | 48.3% | 8.6E-42 |  |
| 169 | + | 135966 | 136643 | 226 | endolysin [*Synechococcus* phage S-B68] | lysozyme | 67.4% | 3.3E-100 | COG3179 (1.7E-27) |
|  |  |  |  |  | endolysin [*Synechococcus* phage S-B28] |  | 59.1% | 1.3E-81 |  |
|  |  |  |  |  | endolysin [*Synechococcus* phage S-N03] |  | 67.2% | 3.9E-80 |  |
|  |  |  |  |  | endolysin [*Synechococcus* phage S-H34] |  | 67.2% | 1.4E-79 |  |
| 170 | + | 136674 | 137315 | 214 | hypothetical protein PQC09_gp239 [*Synechococcus* phage S-N03] | lysozyme | 62.1% | 7.7E-82 | cd00736 (7.2E-04) |
|  |  |  |  |  | hypothetical protein PQC15_gp236 [*Synechococcus* phage S-H34] |  | 60.7% | 2.5E-80 |  |
|  |  |  |  |  | hypothetical protein [*Synechococcus* phage S-B68] |  | 56.5% | 4.1E-73 |  |
|  |  |  |  |  | structural protein [*Synechococcus* phage S-CBS2] |  | 48.6% | 4.1E-55 |  |
| 171 | + | 137334 | 137612 | 93 | hypothetical protein [bacterium] |  | 32.9% | 1.7E-08 |  |
| 172 | + | 137593 | 138195 | 201 | hypothetical protein PQC15_gp237 [*Synechococcus* phage S-H34] |  | 55.7% | 5.1E-12 |  |
|  |  |  |  |  | hypothetical protein PQC09_gp240 [*Synechococcus* phage S-N03] |  | 52.9% | 1.8E-10 |  |
| 173 | + | 138230 | 138436 | 69 | hypothetical protein PQC15_gp238 [*Synechococcus* phage S-H34] |  | 79.4% | 3.4E-28 |  |
|  |  |  |  |  | hypothetical protein [*Synechococcus* phage S-B68] |  | 74.2% | 3.8E-25 |  |
|  |  |  |  |  | hypothetical protein PQC09_gp241 [*Synechococcus* phage S-N03] |  | 63.5% | 1.2E-16 |  |
|  |  |  |  |  | hypothetical protein AU107_gp115 [Cyanophage P-TIM40] |  | 75% | 1.1E-14 |  |
| 174 | + | 138460 | 138900 | 147 | hypothetical protein [*Synechococcus* phage S-B68] |  | 62.1% | 4.6E-61 |  |
|  |  |  |  |  | hypothetical protein PQC15_gp239 [*Synechococcus* phage S-H34] |  | 49.3% | 4.9E-30 |  |
| 175 | + | 139029 | 139196 | 56 | hypothetical protein PQC09_gp242 [*Synechococcus* phage S-N03] |  | 50% | 3.2E-08 |  |
| 176 | + | 139308 | 140018 | 237 | tail fibers protein [*Synechococcus* phage S-B68] |  | 82.1% | 6.9E-139 |  |
|  |  |  |  |  | hypothetical protein SRSM4_124 [*Synechococcus* phage S-RSM4] |  | 57.3% | 9.1E-87 |  |
|  |  |  |  |  | hypothetical protein PQC15_gp245 [*Synechococcus* phage S-H34] |  | 31.9% | 4.4E-23 |  |
| 177 | + | 140059 | 140466 | 136 | HNH endonuclease [*Synechococcus* phage S-H34] | HNH endonuclease | 62.2% | 4.2E-58 | pfam14279 (1.3E-14) |
|  |  |  |  |  | HNH endonuclease [*Synechococcus* phage S-N03] |  | 60.7% | 1.7E-56 |  |
|  |  |  |  |  | hypothetical protein [*Synechococcus* phage S-B68] |  | 60.2% | 1.5E-49 |  |
| 179 | + | 141086 | 141379 | 98 | hypothetical protein PQC09_gp183 [*Synechococcus* phage S-N03] |  | 65% | 4.1E-39 |  |
|  |  |  |  |  | hypothetical protein PQC15_gp229 [*Synechococcus* phage S-H34] |  | 68.5% | 1.6E-38 |  |
|  |  |  |  |  | hypothetical protein Syn7803C14_48 [*Synechococcus* phage ACG-2014f] |  | 44.8% | 7.9E-19 |  |
|  |  |  |  |  | hypothetical protein BOW86_gp061 [*Synechococcus* phage S-CAM7] |  | 40.2% | 1.9E-13 |  |
| 180 | + | 141647 | 141808 | 54 | hypothetical protein PQC09_gp181 [*Synechococcus* phage S-N03] |  | 60.4% | 1.2E-11 |  |
|  |  |  |  |  | hypothetical protein HOT80_gp45 [*Synechococcus* T7-like phage S-TIP37] |  | 48% | 2.3E-10 |  |
|  |  |  |  |  | hypothetical protein PQC15_gp226 [*Synechococcus* phage S-H34] |  | 54.7% | 2.8E-10 |  |
| 182 | + | 142075 | 142326 | 84 | hypothetical protein PQC09_gp180 [*Synechococcus* phage S-N03] |  | 64.7% | 9.5E-24 |  |
|  |  |  |  |  | hypothetical protein PQC15_gp225 [*Synechococcus* phage S-H34] |  | 61.8% | 7.8E-22 |  |
|  |  |  |  |  | hypothetical protein [*Synechococcus* phage S-B68] |  | 48.5% | 1.3E-15 |  |
|  |  |  |  |  | hypothetical protein BOW86_gp237 [*Synechococcus* phage S-CAM7] |  | 45.5% | 2.9E-09 |  |
|  |  |  |  |  | hypothetical cyanophage protein [*Synechococcus* phage S-CRM01] |  | 41.3% | 2.0E-08 |  |
| 183 | + | 142326 | 142910 | 195 | hypothetical protein [*Synechococcus* phage S-B68] | transcription antitermination protein NusG | 68.4% | 1.2E-96 | PRK05609 (6.3E-26) |
|  |  |  |  |  | antitermination factor [*Synechococcus* phage S-N03] |  | 68.6% | 2.6E-95 |  |
|  |  |  |  |  | antitermination factor [*Synechococcus* phage S-H34] |  | 67.5% | 4.0E-95 |  |
|  |  |  |  |  | transcription antiterminator [*Synechococcus* phage S-CRM01] |  | 51% | 5.9E-60 |  |
| 185 | + | 143070 | 143258 | 63 | hypothetical protein PQC15_gp222 [*Synechococcus* phage S-H34] |  | 60.3% | 3.8E-18 |  |
|  |  |  |  |  | hypothetical protein [*Synechococcus* phage S-B68] |  | 63.5% | 8.8E-18 |  |
|  |  |  |  |  | hypothetical protein PQC09_gp177 [*Synechococcus* phage S-N03] |  | 58.7% | 3.1E-17 |  |
| 186 | + | 143258 | 143527 | 90 | glutaredoxin [*Synechococcus* phage S-B68] | glutaredoxin | 61.8% | 1.9E-25 | cd02066 (9.2E-11) |
|  |  |  |  |  | glutaredoxin [*Synechococcus* phage S-CRM01] |  | 51.4% | 4.9E-21 |  |
|  |  |  |  |  | glutaredoxin [*Synechococcus* phage S-N03] |  | 54.6% | 9.0E-20 |  |
|  |  |  |  |  | glutaredoxin [*Synechococcus* phage S-H34] |  | 52.6% | 3.5E-19 |  |
|  |  |  |  |  | glutaredoxin [*Synechococcus* phage Bellamy] |  | 46.8% | 4.8E-18 |  |
| 188 | + | 143689 | 144000 | 104 | hypothetical protein [*Synechococcus* phage S-B68] |  | 47.4% | 2.7E-19 |  |
|  |  |  |  |  | hypothetical protein PQC09_gp175 [*Synechococcus* phage S-N03] |  | 40.8% | 2.3E-11 |  |
|  |  |  |  |  | hypothetical protein PQC15_gp219 [*Synechococcus* phage S-H34] |  | 38.8% | 2.5E-10 |  |
|  |  |  |  |  | hypothetical cyanophage protein [*Synechococcus* phage S-CRM01] |  | 33.3% | 2.7E-06 |  |
| 189 | + | 144084 | 144926 | 281 | exonuclease [*Synechococcus* phage S-H34] | ribonuclease H | 76.4% | 1.1E-160 | PHA02567 (1.3E-83) |
|  |  |  |  |  | ribonuclease H [*Synechococcus* phage S-B68] |  | 76.1% | 1.1E-159 |  |
|  |  |  |  |  | exonuclease [*Synechococcus* phage S-N03] |  | 74.6% | 2.8E-158 |  |
|  |  |  |  |  | RNaseH ribonuclease [*Synechococcus* phage S-CRM01] |  | 70.7% | 2.0E-147 |  |
|  |  |  |  |  | exonuclease [*Synechococcus* phage S-H38] |  | 46.5% | 9.2E-84 |  |
| 190 | + | 144929 | 145432 | 168 | hypothetical protein [*Synechococcus* phage S-B68] |  | 81.4% | 2.1E-95 |  |
|  |  |  |  |  | hypothetical protein PQC09_gp173 [*Synechococcus* phage S-N03] |  | 77.1% | 9.9E-92 |  |
|  |  |  |  |  | hypothetical protein PQC15_gp217 [*Synechococcus* phage S-H34] |  | 76.5% | 1.0E-91 |  |
|  |  |  |  |  | hypothetical cyanophage protein [*Synechococcus* phage S-CRM01] |  | 75.5% | 4.2E-89 |  |
|  |  |  |  |  | hypothetical protein SBM3_00068 [*Synechococcus* phage S-BM3] |  | 43.2% | 1.1E-34 |  |
|  |  |  |  |  | DNA ligase [*Synechococcus* phage DSL-LC02] |  | 43.9% | 4.7E-34 |  |
| 192 | + | 145675 | 146334 | 220 | thymidylate synthase [*Synechococcus* phage S-H34] | thymidylate synthase (*thyX*) | 85% | 2.0E-136 | PRK00847 (1E-67) |
|  |  |  |  |  | thymidylate synthase [*Synechococcus* phage S-N03] |  | 83.1% | 3.8E-134 |  |
|  |  |  |  |  | thymidylate synthase [*Synechococcus* phage S-B68] |  | 78.9% | 3.1E-128 |  |
|  |  |  |  |  | thymidylate synthase [Cyanophage KBS-S-1A] |  | 60.4% | 5.1E-87 |  |
|  |  |  |  |  | hypothetical protein [*Pseudomonadota* bacterium] |  | 60.7% | 6.6E-18 |  |
| 193 | + | 146331 | 146531 | 67 | hypothetical protein PQC13_gp239 [*Synechococcus* phage S-SRM01] |  | 62.8% | 2.6E-15 |  |
|  |  |  |  |  | hypothetical protein [*Synechococcus* phage S-B68] |  | 53.2% | 9.4E-15 |  |
|  |  |  |  |  | hypothetical protein PQC15_gp214 [*Synechococcus* phage S-H34] |  | 54% | 2.1E-13 |  |
|  |  |  |  |  | hypothetical protein PQC09_gp170 [*Synechococcus* phage S-N03] |  | 54.7% | 2.7E-12 |  |
|  |  |  |  |  | hypothetical cyanophage protein [*Synechococcus* phage S-CRM01] |  | 52.1% | 1.4E-07 |  |
| 194 | + | 146555 | 146806 | 84 | hypothetical protein [*Synechococcus* phage S-B68] |  | 75.9% | 1.4E-38 |  |
|  |  |  |  |  | transcriptional regulator [*Synechococcus* phage S-CRM01] |  | 72.3% | 4.2E-34 |  |
|  |  |  |  |  | transcriptional regulator [*Synechococcus* phage S-N03] |  | 66.3% | 4.5E-32 |  |
|  |  |  |  |  | transcriptional regulator [*Synechococcus* phage S-H34] |  | 61.5% | 4.0E-29 |  |
|  |  |  |  |  | transcriptional regulator [*Synechococcus* phage ACG-2014f] |  | 58.5% | 6.0E-24 |  |
|  |  |  |  |  | hypothetical protein b23_0382 [*Synechococcus* phage B23] |  | 52.7% | 1.7E-19 |  |
|  |  |  |  |  | phosphate starvation-inducible protein [*Synechococcus* phage S-B68] |  | 71% | 6.0E-126 |  |
| 195 | + | 146806 | 147522 | 239 | PhoH-like phosphate starvation-inducible [*Synechococcus* phage S-H34] | phosphate starvation-inducible protein (*phoH*) | 71.5% | 7.8E-125 | COG1875 (5.2E-32) |
|  |  |  |  |  | PhoH-like phosphate starvation-inducible [*Synechococcus* phage S-N03] |  | 71.4% | 1.2E-124 |  |
|  |  |  |  |  | PhoH-like phosphate starvation-inducible [*Synechococcus* phage S-CRM01] |  | 63.3% | 4.1E-111 |  |
|  |  |  |  |  | P-starvation inducible protein [*Synechococcus* phage S-BM3] |  | 62.4% | 1.4E-110 |  |
| 196 | + | 147522 | 148211 | 230 | exonuclease A [Synechococcus phage S-B68] |  | 80.6% | 3.0E-134 |  |
|  |  |  |  |  | exonuclease [*Synechococcus* phage S-H34] |  | 65.1% | 6.8E-101 |  |
|  |  |  |  |  | exonuclease [*Synechococcus* phage S-N03] |  | 62.5% | 4.4E-97 |  |
|  |  |  |  |  | exonuclease [*Synechococcus* phage S-BM3] |  | 45.8% | 1.8E-59 |  |
|  |  |  |  |  | exonuclease [*Synechococcus* phage S-CRM01] |  | 51.8% | 4.2E-48 |  |
| 197 | + | 148204 | 148464 | 87 | late promoter transcriptional regulator [*Synechococcus* phage S-CRM01] | late-transcription coactivator | 71.1% | 7.5E-34 | pfam16805 (1.1E-23) |
|  |  |  |  |  | late promoter transcriptional accessory protein [*Synechococcus* phage S-B68] |  | 66.3% | 2.5E-31 |  |
|  |  |  |  |  | late promoter transcriptional regulator [*Synechococcus* phage S-H34] |  | 62.9% | 3.7E-30 |  |
|  |  |  |  |  | late promoter transcriptional regulator [*Synechococcus* phage S-N03] |  | 60.7% | 1.1E-28 |  |
|  |  |  |  |  | hypothetical protein CYVG_00184 [Cyanophage S-SSM6a] |  | 63% | 2.6E-28 |  |
| 198 | + | 148516 | 149127 | 204 | loader of DNA helicase [*Synechococcus* phage S-H34] | DNA helicase assembly protein | 65.7% | 1.5E-99 | PHA02559 (1.9E-43) |
|  |  |  |  |  | loader of DNA helicase [*Synechococcus* phage S-N03] |  | 65.7% | 2.1E-98 |  |
|  |  |  |  |  | hypothetical protein [*Synechococcus* phage S-B68] |  | 64.7% | 2.0E-97 |  |
|  |  |  |  |  | DNA helicase loader [*Synechococcus* phage S-CRM01] |  | 51.2% | 8.8E-76 |  |
|  |  |  |  |  | DNA helicase loader [*Synechococcus* phage S-CAM7] |  | 38.7% | 7.5E-52 |  |
| 199 | + | 149124 | 149501 | 126 | hypothetical protein PQC15_gp208 [*Synechococcus* phage S-H34] |  | 50% | 1.9E-31 |  |
|  |  |  |  |  | hypothetical protein PQC09_gp164 [*Synechococcus* phage S-N03] |  | 46.2% | 2.9E-31 |  |
| 200 | + | 149576 | 150523 | 316 | single stranded DNA-binding protein [*Synechococcus* phage S-B68] | single stranded DNA-binding protein | 76.8% | 4.7E-172 | PHA02550 (9.9E-119) |
|  |  |  |  |  | single-stranded DNA-binding protein [*Synechococcus* phage S-N03] |  | 76.5% | 4.9E-171 |  |
|  |  |  |  |  | single-stranded DNA-binding protein [*Synechococcus* phage S-H34] |  | 75.4% | 1.2E-170 |  |
|  |  |  |  |  | single strand DNA binding protein [*Synechococcus* phage S-CRM01] |  | 61.1% | 9.3E-126 |  |
|  |  |  |  |  | ssDNA binding protein [*Synechococcus* phage S-SCSM1] |  | 55% | 7.4E-110 |  |
| 201 | + | 150585 | 151256 | 224 | tail tube [*Synechococcus* phage S-N03] |  | 49.8% | 7.3E-66 |  |
|  |  |  |  |  | tail tube [*Synechococcus* phage S-H34] |  | 48.7% | 1.7E-59 |  |
|  |  |  |  |  | hypothetical protein [*Synechococcus* phage S-B68] |  | 49.3% | 5.7E-59 |  |
|  |  |  |  |  | hypothetical cyanophage protein [*Synechococcus* phage S-CRM01] |  | 39.5% | 1.6E-44 |  |
| 202 | + | 151256 | 151600 | 115 | hypothetical protein PQC09_gp158 [*Synechococcus* phage S-N03] |  | 62.3% | 2.1E-44 |  |
|  |  |  |  |  | hypothetical protein [*Synechococcus* phage S-B68] |  | 60.4% | 1.9E-42 |  |
|  |  |  |  |  | hypothetical protein PQC15_gp202 [*Synechococcus* phage S-H34] |  | 62.3% | 5.0E-38 |  |
|  |  |  |  |  | hypothetical cyanophage protein [*Synechococcus* phage S-CRM01] |  | 49.6% | 7.6E-33 |  |
|  |  |  |  |  | hypothetical protein PSSM2_011 [*Prochlorococcus* phage P-SSM2] |  | 43.2% | 1.1E-25 |  |
| 203 | + | 151597 | 152421 | 275 | hypothetical protein [*Synechococcus* phage S-B68] |  | 48.6% | 1.1E-82 |  |
|  |  |  |  |  | baseplate tail tube cap [*Synechococcus* phage S-N03] |  | 44.2% | 1.1E-69 |  |
|  |  |  |  |  | baseplate tail tube cap [*Synechococcus* phage S-H34] |  | 46% | 4.5E-69 |  |
|  |  |  |  |  | baseplate tail tube cap [*Synechococcus* phage S-CRM01] |  | 43.1% | 7.8E-67 |  |
|  |  |  |  |  | baseplate tail tube cap [*Synechococcus* phage Bellamy] |  | 31.9% | 2.5E-22 |  |
| 204 | + | 152421 | 153053 | 211 | hypothetical protein [*Synechococcus* phage S-B68] | baseplate wedge protein, inner | 47.1% | 3.1E-61 | PHA02578 (3.1E-10) |
|  |  |  |  |  | baseplate wedge subunit [*Synechococcus* phage S-CRM01] |  | 44.4% | 1.2E-56 |  |
|  |  |  |  |  | tail sheath [*Synechococcus* phage S-N03] |  | 43.5% | 8.0E-55 |  |
|  |  |  |  |  | baseplate wedge subunit [*Synechococcus* phage S-H34] |  | 43.1% | 1.9E-52 |  |
|  |  |  |  |  | baseplate wedge subunit [Cyanophage P-RSM6] |  | 37.4% | 1.3E-33 |  |
| 205 | + | 153050 | 153505 | 152 | head closure [*Synechococcus* phage S-H34] | head completion nuclease | 65.6% | 4.6E-72 | PHA02552 (5.3E-54) |
|  |  |  |  |  | head closure [*Synechococcus* phage S-N03] |  | 66.2% | 5.6E-71 |  |
|  |  |  |  |  | head completion protein [*Synechococcus* phage S-B68] |  | 71.3% | 9.5E-70 |  |
|  |  |  |  |  | head closure [*Synechococcus* phage S-SCSM1] |  | 56.4% | 3.5E-59 |  |
| 206 | + | 153547 | 154254 | 236 | baseplate hub [*Synechococcus* phage S-N03] | baseplate assembly chaperone | 67.2% | 8.2E-117 | pfam12322 (9.4E-15) |
|  |  |  |  |  | baseplate hub [*Synechococcus* phage S-H34] |  | 66.4% | 3.4E-115 |  |
|  |  |  |  |  | baseplate assembly chaperone [*Synechococcus* phage S-B68] |  | 66% | 1.1E-109 |  |
|  |  |  |  |  | baseplate hub subunit [*Synechococcus* phage S-CRM01] |  | 52.3% | 1.2E-87 |  |
|  |  |  |  |  | baseplate hub subunit [*Synechococcus* phage S-BM3] |  | 43.4% | 5.6E-64 |  |
| 207 | + | 154260 | 154457 | 66 | hypothetical protein [*Synechococcus* phage S-B68] |  | 70.5% | 2.9E-25 |  |
|  |  |  |  |  | baseplate hub assembly catalyst [*Synechococcus* phage S-H34] |  | 72.1% | 8.6E-25 |  |
|  |  |  |  |  | baseplate hub assembly catalyst [*Synechococcus* phage S-N03] |  | 72.1% | 1.5E-24 |  |
|  |  |  |  |  | baseplate hub assembly catalyst [*Synechococcus* phage S-CRM01] |  | 58.6% | 8.9E-19 |  |
|  |  |  |  |  | baseplate hub assembly catalyst [*Synechococcus* phage S-SSM5] |  | 43.3% | 4.4E-08 |  |
| 208 | + | 154483 | 159180 | 1566 | membrane-bound lytic murein transglycosylase D precursor [*Synechococcus* phage S-B68] |  | 53% | 1.7E-74 |  |
|  |  |  |  |  | chitinase [*Synechococcus* phage S-H34] |  | 42.8% | 9.9E-38 |  |
| 209 | + | 159184 | 163554 | 1457 | endolysin [*Synechococcus* phage S-N03] | endolysin | 58.8% | 1.2E-61 | cd00737 (2.4E-12) |
|  |  |  |  |  | endolysin [*Synechococcus* phage S-SSM4] |  | 70.1% | 1.0E-60 |  |
|  |  |  |  |  | TolA protein [*Synechococcus* phage S-B68] |  | 57.4% | 5.4E-60 |  |
|  |  |  |  |  | endolysin [*Synechococcus* phage S-H34] |  | 57.8% | 2.3E-56 |  |
|  |  |  |  |  | hypothetical protein SBM1_00143 [*Synechococcus* phage S-BM1] |  | 44.9% | 1.3E-24 |  |
| 210 | + | 163557 | 164837 | 427 | tail protein [*Synechococcus* phage S-N03] |  | 56% | 1.4E-174 |  |
|  |  |  |  |  | hypothetical protein [*Synechococcus* phage S-B68] |  | 55.3% | 1.9E-170 |  |
|  |  |  |  |  | tail protein [*Synechococcus* phage S-H34] |  | 54.9% | 2.4E-170 |  |
|  |  |  |  |  | tail protein [*Synechococcus* phage S-CRM01] |  | 46.7% | 1.7E-137 |  |
|  |  |  |  |  | tail protein [*Synechococcus* phage S-T4] |  | 24.9% | 1.9E-16 |  |
| 211 | + | 164834 | 167272 | 813 | hypothetical protein [*Synechococcus* phage S-B68] |  | 44.4% | 0 |  |
|  |  |  |  |  | baseplate hub subunit and tail lysozyme [*Synechococcus* phage S-CRM01] |  | 38.3% | 4.8E-174 |  |
|  |  |  |  |  | baseplate hub subunit and tail lysozyme [*Synechococcus* phage S-N03] |  | 49.8% | 8.6E-150 |  |
|  |  |  |  |  | baseplate hub subunit and tail lysozyme [*Synechococcus* phage S-H34] |  | 48.8% | 2.6E-147 |  |
|  |  |  |  |  | baseplate hub + tail lysozyme [*Synechococcus* phage S-BM3] |  | 28.1% | 2.7E-27 |  |
| 212 | + | 167269 | 168228 | 320 | hypothetical protein PQC15_gp193 [*Synechococcus* phage S-H34] |  | 55.4% | 3.6E-131 |  |
|  |  |  |  |  | hypothetical protein PQC09_gp149 [*Synechococcus* phage S-N03] |  | 55.7% | 2.0E-128 |  |
|  |  |  |  |  | hypothetical protein [*Synechococcus* phage S-B68] |  | 57.5% | 5.3E-116 |  |
|  |  |  |  |  | hypothetical protein SCRM01_058c [*Synechococcus* phage S-CRM01] |  | 50.3% | 2.7E-105 |  |
| 213 | + | 168230 | 169045 | 272 | hypothetical protein PQC09_gp148 [*Synechococcus* phage S-N03] | baseplate hub subunit and tail lysozyme | 28.6% | 1.1E-11 | PHA02596 (5E-16) |
|  |  |  |  |  | hypothetical protein PQC15_gp192 [*Synechococcus* phage S-H34] |  | 30.6% | 1.2E-09 |  |
| 214 | + | 169042 | 169296 | 85 | hypothetical protein PQC15_gp191 [*Synechococcus* phage S-H34] |  | 47% | 1.1E-12 |  |
|  |  |  |  |  | hypothetical protein PQC09_gp147 [*Synechococcus* phage S-N03] |  | 43.8% | 3.2E-09 |  |
| 215 | + | 169280 | 169624 | 115 | PAAR motif of membran proteins [*Synechococcus* phage S-H34] | central spike protein | 84.4% | 1.5E-48 | cd14737 (1.6E-17) |
|  |  |  |  |  | PAAR motif of membran proteins [*Synechococcus* phage S-N03] |  | 83.9% | 6.4E-47 |  |
|  |  |  |  |  | PAAR motif of membran proteins [*Prochlorococcus* phage P-HM2] |  | 70.5% | 4.0E-38 |  |
|  |  |  |  |  | PAAR protein [*Synechococcus* phage S-B68] |  | 81% | 4.0E-36 |  |
|  |  |  |  |  | PAAR motif of membran proteins [*Synechococcus* phage S-CRM01] |  | 69.9% | 2.0E-27 |  |
| 217 | + | 169774 | 171543 | 590 | hypothetical protein CC030809_00243 [*Synechococcus* phage S-CAM7] | long tail fiber protein | 38% | 4.2E-10 | PHA02584 (8.8E-05) |
| 218 | - | 171585 | 171932 | 116 | hypothetical protein DR97_1744 [*Pseudomonas* *aeruginosa*] |  | 39.2% | 5.0E-08 |  |
| 219 | + | 172309 | 174861 | 851 | hypothetical protein [uncultured Mediterranean phage uvMED] | long tail fiber distal subunit | 48.3% | 1.6E-26 | pfam13884 (1.1E-09) |
|  |  |  |  |  | hypothetical protein OlV7_gene68 [*Ostreococcus lucimarinus* virus 7] |  | 67% | 1.3E-25 |  |

^a^Putative functions are predicted based on the conserved domain search against the Conserved Domain Database.

^b^The best hit of each ORF in the Conserved Domain Database and its e-value.

| Supplementary Table S3. Predicted ORFs in the S-CREM2 genome that have distant homologs detected by HHpred search. | | | | | | | | |
| --- | --- | --- | --- | --- | --- | --- | --- | --- |
| Gene | Strand | Left | Right | aa length | ^a^Putative function | ^b^Hits | Probability | E-value |
| 6 | - | 5081 | 3837 | 415 | S-adenosyl methionine hydrolase | S-adenosyl methionine hydrolase [*Enterobacteria* phage T3] | 99.6% | 1.3E-13 |
| 11 | - | 7623 | 7054 | 190 | restriction endonuclease EcoRI | Restriction endonuclease EcoRI [*Escherichia* *coli*] | 100% | 2.0E-61 |
| 12 | - | 10313 | 7704 | 870 | CRISPR-associated endonuclease Cas9 | CRISPR-associated endonuclease Cas9/Csn1 [*Actinomyces* *naeslundii*] | 92.4% | 0.2 |
| 25 | + | 16152 | 16655 | 167 | SaV protein | SaV protein [*Lactococcus* phage p2] | 99.6% | 3.4E-15 |
| 51 | + | 29672 | 30094 | 140 | intron-associated endonuclease II | intron-associated endonuclease 2 [*Enterobacteria* phage T4] | 99.5% | 3.2E-14 |
| 55 | + | 31017 | 33062 | 681 | ribosome assembly protein | ribosome assembly protein [*Saccharomyces cerevisiae*] | 99% | 4.1E-09 |
| 79 | + | 40296 | 40835 | 179 | dTDP-4-dehydrorhamnose 3,5-epimerase (*rmlC*) | dTDP-4-dehydrorhamnose 3,5-epimerase RmlC [*Salmonella typhimurium*] | 99.9% | 3.8E-21 |
| 84 | + | 42165 | 42554 | 129 | tail fibe attachment protein | L-shaped tail fiber protein p132 [*Escherichia* phage T5] | 99.9% | 1.4E-20 |
| 108 | + | 65598 | 66203 | 201 | metallopeptidase | metallopeptidase [*Pyrococcus abyssi*] | 93.8% | 0.2 |
| 135 | + | 106821 | 107282 | 153 | terminase small subunit | terminase, small subunit [*Enterobacteria* phage T4] | 100% | 2.3-36 |
| 167 | + | 135102 | 135446 | 114 | restriction endonuclease | holliday-junction resolvase [*Sulfolobus* *solfataricus*] | 99.3% | 7.8E-11 |
| 184 | + | 142907 | 143080 | 57 | carbon metabolism regulator (*cp12*) | *cp12* polypeptide [*Thermosynechococcus* *elongatus* (strain BP-1)] | 99.8% | 1.7E-18 |
| 190 | + | 144929 | 145432 | 167 | DNA ligase | DNA ligase [*Enterobacteria* phage T4] | 100% | 6.1E-26 |
| 193 | + | 146331 | 146531 | 66 | rob transcription factor | rob transcription factor [*Escherichia* *coli*] | 96.1% | 1.2E-02 |
| 194 | + | 146555 | 146806 | 83 | RNA polymerase II | RNA polymerase II [Baker's yeast (*Saccharomyces* cerevisiae)] | 98.3% | 2.7E-06 |
| 201 | + | 150585 | 151256 | 223 | tail tube protein | tail tube protein gp19 [*Enterobacteria* phage T4] | 99.9% | 1.2E-24 |
| 202 | + | 151256 | 151600 | 114 | terminal DNA protecting protein | terminal DNA protecting protein [*Enterobacteria* phage T4] | 100% | 1.4E-30 |
| 203 | + | 151597 | 152421 | 274 | baseplate tail-tube protein | baseplate tail-tube junction protein gp48 [*Enterobacteria* phage T4] | 100% | 2.1E-40 |
| 208 | + | 154483 | 159180 | 1565 | endolysin | endolysin [*Mycobacterium* phage D29] | 99.3% | 1.6E-11 |
| 210 | + | 163557 | 164837 | 426 | baseplate central spike | baseplate central spike complex protein gp27 [*Enterobacteria* phage T4] | 100% | 2.3E-42 |
| 211 | + | 164834 | 167272 | 812 | central spike | pre-baseplate central spike protein gp5 [*Enterobacteria* phage T4] | 99.4% | 4.1E-13 |
| 212 | + | 167269 | 168228 | 319 | central spike | pre-baseplate central spike protein gp5 [*Enterobacteria* phage T4] | 99.4% | 2.1E-07 |
| ^a^Putative functions are predicted based on the functions of homologs using the HHpred search.  ^b^The top homologs in PDB, SCOPe, or Uniprot-SwissProt databases. | | | | | | | | |

| Supplementary Table S4. T4-like core genes in the S-CREM2 genome. | | | |
| --- | --- | --- | --- |
| ORF no. in S-CREM2 | Description | Function category | Gene size (bp) |
| 120 | baseplate wedge protein, inner | structure forming and packaging | 1875 |
| 121 | baseplate wedge protein, inner | structure forming and packaging | 1128 |
| 132 | adaptor | structure forming and packaging | 1044 |
| 133 | stopper | structure forming and packaging | 828 |
| 134 | sheath terminator | structure forming and packaging | 462 |
| 135 | terminase small subunit | structure forming and packaging | 600 |
| 140 | tail tube protein | structure forming and packaging | 1824 |
| 141 | portal protein | structure forming and packaging | 642 |
| 143 | prohead core protein protease | structure forming and packaging | 1059 |
| 144 | capsid assembly scaffolding protein | structure forming and packaging | 426 |
| 205 | head completion nuclease | structure forming and packaging | 456 |
| 206 | baseplate hub assembly protein | structure forming and packaging | 708 |
| 26 | sliding clamp gp45 | DNA replication and metabolism | 666 |
| 31 | clamp loader subunit | DNA replication and metabolism | 927 |
| 33 | clamp loader A subunit | DNA replication and metabolism | 396 |
| 44 | DNA primase/41 helicase | DNA replication and metabolism | 411 |
| 148 | recombination, repair and ssDNA binding protein UvsY | DNA replication and metabolism | 486 |
| 162 | DNA primase | DNA replication and metabolism | 996 |
| 163 | ribonucleotide reductase of class Ia (aerobic), alpha subunit (*nrdA*) | DNA replication and metabolism | 2298 |
| 164 | ribonucleotide reductase of class Ia (aerobic), beta subunit (*nrdB*) | DNA replication and metabolism | 1080 |
| 190 | DNA ligase | DNA replication and metabolism | 504 |
| 192 | thymidylate synthase (*thyX*) | DNA replication and metabolism | 660 |
| 200 | single stranded DNA-binding protein | DNA replication and metabolism | 948 |
| 35 | translational repressor of early genes, *regA* | regulation | 1395 |
| 45 | *mazG* | regulation | 888 |
| 153 | RNA polymerase sigma factor | regulation | 861 |
| 195 | phosphate starvation-inducible protein (*phoH*) | regulation | 717 |
| 197 | late-transcription coactivator | regulation | 261 |
| 2 | hypothetical protein | unknown | 279 |
| 87 | hypothetical protein | unknown | 408 |
| 159 | hypothetical protein | unknown | 372 |

| Supplementary Table S5. The virion structural proteins in the S-CREM2 genome that have homologs with T4-phage in the PDB. | | | | | | | | | | | |
| --- | --- | --- | --- | --- | --- | --- | --- | --- | --- | --- | --- |
| Gene | Strand | Left | Right | aa length | Discription | Hit_PDB | Hit | Probability | E-value | Score | Reference |
| 84 | + | 42165 | 42554 | 129 | tail fibe | 7QG9_M | L-shaped tail fiber protein [*Escherichia* phage T5] | 99.9% | 1.4E-20 | 124.7 | Zivanovic et al., 2014 |
| 85 | + | 42559 | 57474 | 4971 | long tail fiber | 4UW8_E | L-shaped tail fiber protein [*Escherichia* phage T5] | 97.8% | 7.5E-05 | 88.1 | Garcia-Doval et al., 2015 |
| 120 | + | 75282 | 75689 | 135 | baseplate wedge protein, inner | 5IW9_B | baseplate wedge protein gp25 [*Enterobacteria* phage T4] | 99.9% | 3.9E-21 | 124.4 | Taylor et al., 2016 |
| 121 | + | 75723 | 77597 | 624 | baseplate wedge protein, inner | 5HX2_E | baseplate wedge protein gp6 [*Enterobacteria* phage T4] | 100% | 3.1E-79 | 695 | Yap et al., 2016 |
| 122 | + | 77594 | 83407 | 1937 | baseplate wedge protein, intermediate | 5HX2_A | baseplate wedge protein gp7 [*Enterobacteria* phage T4] | 99.9% | 3.6E-24 | 273.2 | Yap et al., 2016 |
| 123 | + | 83440 | 84939 | 499 | baseplate wedge protein, intermediate | 1N7Z_D | baseplate structural protein gp8 [*Enterobacteria* phage T4] | 100% | 2.4E-54 | 442.9 | Leiman et al., 2003 |
| 126 | + | 93309 | 94577 | 422 | short tail fiber | 1PDI_M | short tail fiber protein [*Enterobacteria* phage T4] | 94.3% | 0.56 | 43.5 | Kostyuchenko et al., 2003 |
| 132 | + | 103792 | 104919 | 375 | adaptor | 7Z4A_A | adaptor protein [*Escherichia* phage vB_EcoP_SU10] | 93.4% | 15.2 | 31.9 | Liang et al., 2018 |
| 133 | + | 104922 | 105965 | 347 | stopper | 1K0H_A | neck protein gp14[*Enterobacteria* phage T4] | 100% | 2.7E-61 | 28.8 | Cardarelli et al., 2010 |
| 134 | + | 105994 | 106821 | 275 | sheath terminator | 4HUD_E | phage tail terminator protein, gp15 [*Enterobacteria* phage T4] | 100% | 3.2E-62 | 449 | Fokine et al., 2013 |
| 139 | + | 111494 | 113878 | 794 | tail sheath protein | 3J2M_U | tail sheath protein gp18 [*Enterobacteria* phage T4] | 100% | 3.2E-63 | 591.6 | Fokine et al., 2013 |
| 140 | + | 113911 | 114510 | 199 | tail tube protein | 5IV5_S | tail tube protein gp19 [*Enterobacteria* phage T4] | 100% | 4.4E-28 | 180.3 | Taylor et al., 2016 |
| 141 | + | 114599 | 116422 | 607 | portal protein | 6UZC_P | portal protein gp20 [*Enterobacteria* phage T4] | 100% | 1.8E-67 | 587.6 | Fang et al., 2020 |
| 145 | + | 118481 | 119851 | 456 | major capsid protein | 6UZC_k | major capsid protein gp23 [*Enterobacteria* phage T4] | 100% | 3.6E-83 | 681.9 | Fang et al., 2020 |
| 147 | + | 120464 | 121129 | 221 | distal tail protein | 5IV5_GC | baseplate tail-tube protein gp54 [*Enterobacteria* phage T4] | 99.9% | 3.2E-20 | 169.9 | Taylor et al., 2016 |
| 201 | + | 150585 | 151256 | 223 | distal tail protein | 5IV5_p | tail tube protein gp19 [*Enterobacteria* phage T4] | 99.9% | 1.2E-24 | 172.5 | Taylor et al., 2016 |
| 203 | + | 151597 | 152421 | 274 | baseplate wedge protein, inner | 5IV5_DH | baseplate tail-tube junction protein gp48 [*Enterobacteria* phage T4] | 100% | 2.1E-40 | 313.3 | Taylor et al., 2016 |
| 204 | + | 152421 | 153053 | 210 | baseplate wedge protein, inner | 5HX2_F | baseplate wedge protein gp53 [*Enterobacteria* phage T4] | 100% | 2.8E-42 | 287.2 | Yap et al., 2016 |
| 210 | + | 163557 | 164837 | 426 | baseplate central spike | 1WTH_D | baseplate central spike complex protein gp27 [*Enterobacteria* phage T4] | 100% | 2.3E-42 | 285.5 | Kanamaru et al., 2005 |
| 211 | + | 164834 | 167272 | 812 | central spike | 1WTH_A | tail-associated lysozyme gp5 [*Enterobacteria* phage T4] | 98.9% | 6.6E-09 | 122.5 | Kanamaru et al., 2005 |
| 212 | + | 167269 | 168228 | 319 | central spike | 1WTH_A | tail-associated lysozyme gp5 [Bacteriophage T4] | 99.4% | 6.7E-10 | 94.3 | Kanamaru et al., 2005 |
| 215 | + | 169280 | 169624 | 114 | central spike | 4JIW_D | PAAR-repeat central spike tip protein [*Enterobacteria* phage T4] | 98.7% | 1.7E-06 | 53.9 | Shneider et al., 2013 |
| 219 | + | 172309 | 174861 | 850 | long tail fiber distal subunit | 3GW6_F | tail spike protein [*Escherichia* phage K1F] | 99.5% | 2.1E-13 | 161.4 | Petter et al., 1993 |

**Reference**

Cardarelli L, Pell LG, Neudecker P, et al. Phages have adapted the same protein fold to fulfill multiple functions in virion assembly. *Proc* *Natl* *Acad* *Sci U S A*. 2010;107(32):14384-14389. doi:10.1073/pnas.1005822107

Fang Q, Tang WC, Tao P, et al. Structural morphing in a symmetry-mismatched viral vertex. *Nat Commun*. 2020;11(1):1713. doi:10.1038/s41467-020-15575-4

Fokine A, Zhang Z, Kanamaru S, et al. The molecular architecture of the bacteriophage T4 neck. *J Mol Biol*. 2013;425(10):1731-1744. doi:10.1016/j.jmb.2013.02.012

Garcia-Doval C, Castón JR, Luque D, et al. Structure of the receptor-binding carboxy-terminal domain of the bacteriophage T5 L-shaped tail fibre with and without its intra-molecular chaperone. *Viruses*. 2015;7(12):6424-6440. doi:10.3390/v7122946

Kanamaru S, Ishiwata Y, Suzuki T, et al. Control of bacteriophage T4 tail lysozyme activity during the infection process. *J Mol Biol*. 2005;346(4):1013-1020. doi:10.1016/j.jmb.2004.12.042

Kostyuchenko VA, Leiman PG, Chipman PR, et al. Three-dimensional structure of bacteriophage T4 baseplate. *Nat Struct Mol Biol*. 2003;10(9):688-693. doi:10.1038/nsb970

Leiman PG, Shneider MM, Kostyuchenko VA, et al. Structure and location of gene product 8 in the bacteriophage T4 baseplate. *J Mol Biol*. 2003;328(4):821-833. doi:10.1016/s0022-2836(03)00366-8

Liang L, Zhao H, An B, Tang L. High-resolution structure of podovirus tail adaptor suggests repositioning of an octad motif that mediates the sequential tail assembly. *Proc* *Natl* *Acad* *Sci U S A*. 2018;115(2):313-318. doi:10.1073/pnas.1706846115

Petter JG, Vimr ER. Complete nucleotide sequence of the bacteriophage K1F tail gene encoding endo-N-acylneuraminidase (endo-N) and comparison to an endo-N homolog in bacteriophage PK1E. *Journal of bacteriology*. 1993;175(14):4354-4363. doi:10.1128/jb.175.14.4354-4363.1993

Shneider MM, Buth SA, Ho BT, et al. PAAR-repeat proteins sharpen and diversify the type VI secretion system spike. *Nature*. 2013;500(7462):350-353. doi:10.1038/nature12453

Taylor NM, Prokhorov NS, Guerrero-Ferreira RC, et al. Structure of the T4 baseplate and its function in triggering sheath contraction. *Nature*. 2016;533(7603):346-352. doi:10.1038/nature17971

Yap ML, Klose T, Arisaka F, et al. Role of bacteriophage T4 baseplate in regulating assembly and infection. *Proc* *Natl* *Acad* *Sci U S A*. 2016;113(10):2654-2659. doi:10.1073/pnas.1601654113

Zivanovic Y, Confalonieri F, Ponchon L, et al. Insights into bacteriophage T5 structure from analysis of its morphogenesis genes and protein components. *J Virol*. 2014;88(2):1162-1174. doi:10.1128/JVI.02262-13
